# Supplementary material for: Factoring an integer with three oscillators and a qubit
Source: Nat Commun. 2025 Dec 26;17:227. doi: 10.1038/s41467-025-67694-5 (PMC12780048; doi:10.1038/s41467-025-67694-5)
Supplement: Supplementary file 1 — Supplementary Information [file 41467_2025_67694_MOESM1_ESM.pdf]

Supplementary Note

Factoring an integer with three oscillators and a qubit

Lukas Brenner[1,2,*,†], Libor Caha[1,2,*,‡], Xavier Coiteux-Roy[1,2,3,4,5*,§], and Robert Koenig[1,2,*,¶]

[1] School of Computation, Information and Technology, Technical University of Munich, Germany
[2] Munich Center for Quantum Science and Technology, Germany
[3] School of Natural Sciences, Technical University of Munich, Germany
[4] Department of Computer Science, University of Calgary, Canada
[5] Department of Physics and Astronomy, University of Calgary, Canada

Corresponding authors: †lukas.brenner@tum.de, ‡cahalibor@me.com, §xavier.coiteuxroy@ucalgary.ca, ¶robert.koenig@tum.de

*All authors contributed equally to this work.

As a warm-up we analyze the output distribution $p'_{\mathbb{R}}$ of the idealized circuit $\mathcal{Q}^{\mathrm{ideal}}_{a,N}$ (see Eq. (3) in the main text) and argue that it is (formally) uniform on the set $\{j/r \mid j \in \mathbb{Z}\}$.

Proof. We may without loss of generality assume that the Q-quadrature of the second system of the state $U^{\mathrm{ideal}}_{\mathbb{R},a,N}(|\mathsf{GKP}\rangle \otimes M_N\,|\mathsf{GKP}\rangle)$ (see Eq. (4) in the main text) is measured (instead of simply tracing out the system). Since r is the order of the element a in the multiplicative group $\mathbb{Z}_N^*$, the measurement outcome $m \in \mathbb{R}$ has the form $m = \ell \cdot N + a^k$ for some $\ell \in \mathbb{Z}$ and $k \in \{0, \ldots, r-1\}$. The corresponding post-measurement state of the first mode then is the state

$$\sum_{\substack{x\in\mathbb{Z}\\ a^x\equiv a^k \pmod N}} |x\rangle = \sum_{\substack{x\in\mathbb{Z}\\ x\equiv k \pmod r}} |x\rangle = \sum_{j\in\mathbb{Z}} |j\cdot r + k\rangle =: |Ш_{k,r}\rangle \ .$$

Because the state $|Ш_{k,r}\rangle = e^{-ikP}\,|Ш_{0,r}\rangle$ is the result of a translation in position applied to the state $|Ш_r\rangle := |Ш_{0,r}\rangle$, its Fourier transform $\widehat{Ш_{k,r}} = \mathrm{FT}_{\mathbb{R}} Ш_{k,r}$ differs from that of the Dirac comb

$$Ш_r(x) \propto \sum_{j\in\mathbb{Z}} \delta(x - jr)$$

(which is identical to $M_r\,|\mathsf{GKP}\rangle$) only by a p-dependent phase factor, i.e., $\widehat{Ш_{k,r}}(p) = e^{-ikp}\widehat{Ш_{0,r}}(p)$. In particular, this implies that when measuring the momentum of the first mode, the resulting distribution over outcomes $c \in \mathbb{R}$ is independent of k and equal to $p'_{\mathbb{R}}(c) \propto \left|\widehat{Ш_r}(c)\right|^2$. The claim then follows from the fact that the Fourier transform of the Dirac comb is $\widehat{Ш_r}(x) = \frac{1}{r}Ш_{\frac{1}{r}}(x)$, see e.g., [1]. □

In the remainder of this Supplementary Note, we consider the (physically) implementable circuit $\mathcal{Q}_{a,N}$ (see Figure 3 in the main text), which is the quantum subroutine used in our the factorization algorithm. We reproduce it here for the reader's convenience:

$\mathcal{Q}_{a,N}$:=

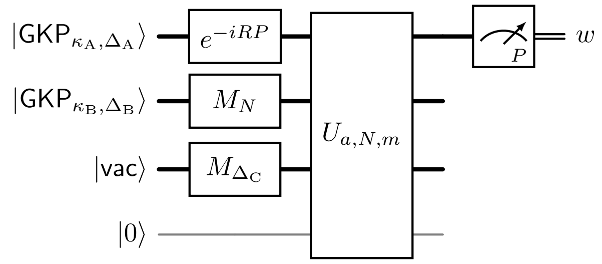

Here $N \in \mathbb{N}$ is an n-bit integer to be factored (i.e., $2^{n-1} \leq N \leq 2^n - 1$), and $a \in \mathbb{Z}_N^*$ is a randomly chosen integer coprime to N. We denote the first, second and third modes and the qubit in the circuit $\mathcal{Q}_{a,N}$ by the labels $\mathrm{A}, \mathrm{B}, \mathrm{C}$ and Q, respectively. The circuit $\mathcal{Q}_{a,N}$ additionally depends on the parameters (R, m) and $(\kappa_\mathrm{A}, \Delta_\mathrm{A}, \kappa_\mathrm{B}, \Delta_\mathrm{B}, \Delta_\mathrm{C})$. In this work, we make the choice of parameters given by Supplementary Table 1.

Unitaries	$m = 17n$ $R = 2^{17n-1}$
system A	$\kappa_\mathrm{A} = \Delta_\mathrm{A} = 2^{-16n}$
system B	$\kappa_\mathrm{B} = \Delta_\mathrm{B} = 2^{-18n^2}$
system C	$\Delta_\mathrm{C} = 2^{-50n}$

Supplementary Table 1. The parameters (N, a, m, R) of the circuit $\mathcal{Q}_{a,N}$ determining what unitaries are used. The parameters $(\kappa_\mathrm{A}, \Delta_\mathrm{A})$, $(\kappa_\mathrm{B}, \Delta_\mathrm{B})$ and Δ_C determine the amount of squeezing in the modes A, B and C, respectively. The table gives a choice of these parameters such that output samples of $\mathcal{Q}_{a,N}$ can be used to factor N, and $\mathcal{Q}_{a,N}$ can be realized by a circuit of depth and size polynomial in n.

We proceed as follows: To develop intuition, we first discuss a version of the quantum subroutine where the initial approximate GKP states $|\mathsf{GKP}_{\kappa_\mathrm{A},\Delta_\mathrm{A}}\rangle$, $|\mathsf{GKP}_{\kappa_\mathrm{B},\Delta_\mathrm{B}}\rangle$ are replaced by idealized GKP states and where the vacuum state $|\mathrm{vac}\rangle$ is replaced by a position-eigenstate localized at $x = 0$ (see Section 1). In Section 2, we prove our main result (Theorem 1 in the main text). We show that the circuit $\mathcal{Q}_{a,N}$ can be used to find a factor of N even when physically meaningful initial states (approximate GKP states) are used. This proof relies on three propositions, which we show in subsequent sections. They can be summarized as follows:

Proposition 1 (shown in Section 3) identifies a sufficient condition for when access to samples of an (N-dependent) probability distribution on $\mathbb{R}$ can be used to efficiently find a factor of N.

Proposition 2 (shown in Section 4) derives an expression for (an approximation to) the state of the circuit $\mathcal{Q}_{a,N}$ before the measurement.

Proposition 3 (shown in Section 6) analyzes the output distribution of $\mathcal{Q}_{a,N}$, and shows that the condition of Proposition 1 is satisfied.

Combined with the efficient preparation procedure for approximate GKP states from [2], Propositions 1–3 imply our main result (Theorem 1 in the main text). Indeed, Propositions 1–3 mean that the output of the circuit $\mathcal{Q}_{a,N}$ can be efficiently post-processed to obtain a factor of N (see Lemma 1 in the main text). To obtain a circuit which does not use approximate GKP initial states as a resource, we use the GKP state preparation protocol from [2] to prepare initial states $\sigma_{\kappa_\mathrm{A},\Delta_\mathrm{A}}, \sigma_{\kappa_\mathrm{B},\Delta_\mathrm{B}} \in \mathcal{B}(L^2(\mathbb{R}))$ close to approximate GKP states in modes A and B, respectively. Finally, we achieve the constant success probability stated in Theorem 1 by repetition.

We need a number of auxiliary concepts and results. In Section 5 we discuss how to perform approximate function evaluation using CV quantum systems. In Section 7, we summarize relevant properties of approximate GKP states. Combinatorial bounds on Gaussian sums used in our analysis are given in Section 8.

Circuits analyzed in this supplementary note

It is convenient to view the circuit $\mathcal{Q}_{a,N}$, as well as other circuits appearing in our analysis, as elements of a family of circuits parametrized by a triple $(\rho_{\mathrm{A}}, \rho_{\mathrm{B}}, \rho_{\mathrm{C}})$ of single-mode initial states which may or may not be mixed. For each such triple, we define a circuit $\mathcal{W}_{a,N}(\rho_{\mathrm{A}}, \rho_{\mathrm{B}}, \rho_{\mathrm{C}})$ by replacing the initial states (up to the unitaries e^{-iRP} on A and $M_{\Delta_{\mathrm{C}}}$ on C) in the circuit $\mathcal{Q}_{a,N}$ by $(\rho_{\mathrm{A}}, \rho_{\mathrm{B}}, \rho_{\mathrm{C}})$, i.e., we set

$\mathcal{W}_{a,N}(\rho_{\mathrm{A}}, \rho_{\mathrm{B}}, \rho_{\mathrm{C}}) :=$

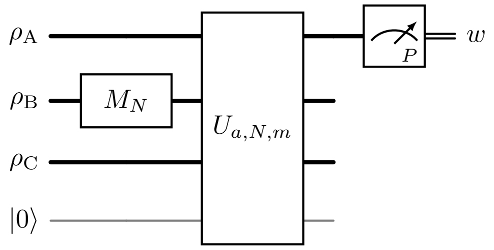

With this convention, the circuits we analyze can be described as follows:

1. The circuit $\mathcal{Q}_{a,N}$ is equal to

$$\mathcal{Q}_{a,N} = \mathcal{W}_{a,N}(e^{-iRP}\mathsf{GKP}_{\kappa_{\mathrm{A}},\Delta_{\mathrm{A}}}, \mathsf{GKP}_{\kappa_{\mathrm{B}},\Delta_{\mathrm{B}}}, \Psi_{\Delta_{\mathrm{C}}}) \ ,$$

   where $\Psi_{\Delta_{\mathrm{C}}} := M_{\Delta_{\mathrm{C}}} |\mathrm{vac}\rangle$ is a squeezed vacuum state.

2. The idealized circuit we discuss in Section 1.5 to develop intuition is

$$\mathcal{W}_{a,N}(\mathrm{e}^{-iRP}\text{Ш}_R, \mathsf{GKP}, |0\rangle) \ ,$$

   where

$$|\text{Ш}_1^{(R)}\rangle \propto \sum_{x=-R}^{R-1} |x\rangle \qquad (1)$$

   is a (formal) superposition of position-eigenstates $|x\rangle$, where $x \in \{-R, \ldots, R-1\}$,

$$|\mathsf{GKP}\rangle \propto \sum_{x\in\mathbb{Z}} |x\rangle \qquad (2)$$

   is an idealized GKP state, and $|0\rangle$ is the position-eigenstate with eigenvalue $x = 0$.

3. The circuit used to obtain Theorem 1 uses states $\sigma_{\kappa_{\mathrm{A}},\Delta_{\mathrm{A}}}$ and $\sigma_{\kappa_{\mathrm{B}},\Delta_{\mathrm{B}}}$ produced by our approximate GKP state preparation protocol [2] in modes A and B, with a displacement applied to mode A, as well as a squeezed vacuum state $\Psi_{\Delta_{\mathrm{C}}}$ in mode C, i.e., it amounts to the circuit

$$\mathcal{W}_{a,N}(e^{-iRP}\sigma_{\kappa_{\mathrm{A}},\Delta_{\mathrm{A}}}e^{iRP}, \sigma_{\kappa_{\mathrm{B}},\Delta_{\mathrm{B}}}, \Psi_{\Delta_{\mathrm{C}}}) \ .$$

In the proof of Proposition 2, we will also use the following intermediate circuit, which depends on three "truncation" parameters $(\varepsilon_{\mathrm{A}}, \varepsilon_{\mathrm{B}}, \varepsilon_{\mathrm{C}})$:

4. Given $\varepsilon_{\mathrm{A}}, \varepsilon_{\mathrm{B}}, \varepsilon_{\mathrm{C}} \in (0, 1/2)$, we consider the circuit

$$\mathcal{W}_{a,N}(c \cdot \Pi_{[-1/2,2R-1/2]} e^{-iRP} \mathsf{GKP}^{\varepsilon_{\mathrm{A}}}_{\kappa_{\mathrm{A}},\Delta_{\mathrm{A}}}, \mathsf{GKP}^{\varepsilon_{\mathrm{B}}}_{\kappa_{\mathrm{B}},\Delta_{\mathrm{B}}}, \Psi^{\varepsilon_{\mathrm{C}}}_{\Delta_{\mathrm{C}}}) ,$$

where we define $\Pi_{\mathcal{C}}$ as the orthogonal projection onto the subspace of $L^2(\mathbb{R})$ of functions supported on the set $\mathcal{C} \subseteq \mathbb{R}$, i.e., $\Pi_{\mathcal{C}}$ acts on $L^2(\mathbb{R})$ as pointwise multiplication by the characteristic function of the set $\mathcal{C}$. The factor c is a normalization constant. We define the union of intervals of length 2ε centered around integers as $\mathbb{Z}(\varepsilon) = \mathbb{Z} + [-\varepsilon, \varepsilon] = \{x + y \mid x \in \mathbb{Z},\ y \in \mathbb{R},\ |y| \leq \varepsilon\}$. The state $\mathsf{GKP}^{\varepsilon}_{\kappa,\Delta} = \Pi_{\mathbb{Z}(\varepsilon)} \mathsf{GKP}_{\kappa,\Delta} / \|\Pi_{\mathbb{Z}(\varepsilon)} \mathsf{GKP}_{\kappa,\Delta}\|$ is then obtained by truncating the support of the approximate GKP state $\mathsf{GKP}_{\kappa,\Delta}$ to $\mathbb{Z}(\varepsilon)$. Similarly, $\Psi^{\varepsilon}_{\Delta} = \Pi_{[-\varepsilon,\varepsilon]} \Psi_{\Delta} / \|\Pi_{[-\varepsilon,\varepsilon]} \Psi_{\Delta}\|$ is the result of truncating the support of the Gaussian state Ψ_{Δ} to the interval $[-\varepsilon, \varepsilon]$.

Contents

1 Action of the circuit $\mathcal{W}_{a,N}(e^{-iRP}Ш_1^{(R)}, \mathsf{GKP}, |0\rangle)$ 6

1.1 Action of composite unitaries . . . 6

1.1.1 Scalar multiplication . . . 7

1.1.2 Translation by R . . . 7

1.1.3 Qubit-controlled scalar multiplication . . . 8

1.1.4 Coherent extraction of the least significant bit (LSB) . . . 9

1.2 The unitary V_α . . . 10

1.3 Controlled multiplication by a pseudomodular power . . . 11

1.4 Controlled translation by a pseudomodular power . . . 14

1.5 Analysis of the circuit $\mathcal{W}_{a,N}(e^{-iRP}Ш_1^{(R)}, \mathsf{GKP}, |0\rangle)$ . . . 15

2 Circuit analysis with finitely squeezed GKP states 17

2.1 Definition of approximate GKP states . . . 17

2.2 Proof of Lemma 1 and Theorem 1: Efficient quantum algorithm for factoring . . 19

3 Proof of Proposition 1: Classical post-processing 23

4 Proof of Proposition 2: Output state of circuit 27

4.1 Distance bounds from properties of approximate GKP states . . . 30

5 Approximate function evaluation for CV systems 38

5.1 Exact function evaluation . . . 38

5.2 Framework for approximate function evaluation . . . 40

5.3 Approximate evaluation of functions $h : \mathbb{R}^m \to \{0, 1\}$ . . . 41

5.4 Approximate function evaluation by various building blocks . . . 44

5.4.1 Approximate evaluation of the least significant bit extraction . . . 44

5.4.2 Approximate function evaluation by the unitaries V_α, $V_{a,N,m}$, and $U_{a,N,m}$ 47

5.5 Implications for the algorithm . . . 60

6 Proof of Proposition 3: Distribution of measurement outcomes 63

6.1 The Fourier transform of Φ_a . . . 63

6.2 Bounds on the Fourier transform . . . 64

6.3 Bounds on the probability distribution of outcomes . . . 67

7 Properties of approximate GKP states 70

8 Combinatorial bounds 74

8.1 Bounds on sums of Gaussians . . . 74

8.2 A tail bound for discrete Gaussian distributions . . . 76

8.3 A bound derived from Jacobi's triple product identity . . . 76

Supplementary References 80

1 Action of the circuit $\mathcal{W}_{a,N}(e^{-iRP}Ш_1^{(R)}, \mathsf{GKP}, |0\rangle)$

In this section, we give a high-level overview of the functionality of our circuit $\mathcal{Q}_{a,N}$ (Figure 3 in the main text), as well as its various building blocks. To do so, we consider idealized initial states which are defined in terms of the position-"eigenstates" $|x\rangle$ of the position (quadrature) operator Q. Here $|x\rangle$ denotes the eigenstate of Q with eigenvalue $x \in \mathbb{R}$. Strictly speaking, these are distributions, and so are most of the expressions we deal with in this section. This discussion is for illustrative purposes only. We shall give a rigorous version of these arguments dealing with normalizable functions (i.e., elements of $L^2(\mathbb{R})$) in Section 2.

Recall that we obtain the circuit $\mathcal{W}_{a,N}(e^{-iRP}Ш_1^{(R)}, \mathsf{GKP}, |0\rangle)$ (cf. Eq. (1) and Eq. (2)) from the circuit $\mathcal{Q}_{a,N}$ by replacing the initial state of

1. mode A by the formal uniform superposition $e^{-iRP}|Ш_1^{(R)}\rangle = e^{-iRP}\sum_{x=-R}^{R-1}|x\rangle = \sum_{x=0}^{2R-1}|x\rangle$ of position-eigenstates $\{|x\rangle\}_{x=0}^{2R-1}$,
2. mode B by the idealized GKP state $|\mathsf{GKP}\rangle \propto \sum_{y\in\mathbb{Z}}|y\rangle$, defined as the (formal) uniform superposition of position-eigenstates with integer positions $y \in \mathbb{Z}$,
3. mode C by the position-eigenstate $|0\rangle$ localized at $x = 0$.

The corresponding circuit $\mathcal{W}_{a,N}(e^{-iRP}Ш_1^{(R)}, \mathsf{GKP}, |0\rangle)$ is shown in Supplementary Figure 1 with intermediate states Ψ_0, Ψ_1, Ψ_2.

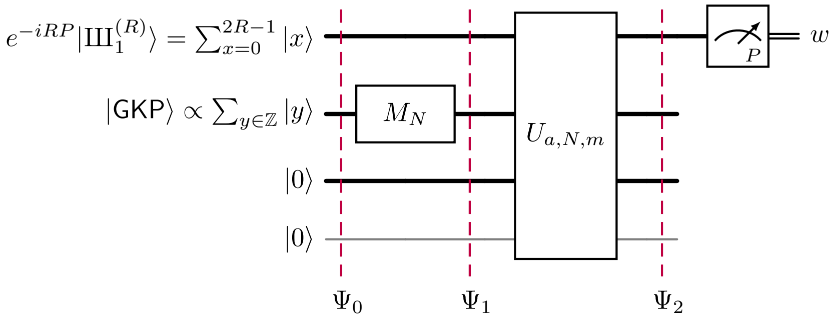

Supplementary Figure 1. The circuit $\mathcal{W}_{a,N}(e^{-iRP}Ш_1^{(R)}, \mathsf{GKP}, |0\rangle)$ which uses idealized initial states. We indicate intermediate states (distributions) Ψ_0, Ψ_1, Ψ_2 that are used in our analysis in Section 1.5.

To compute the state Ψ_2 produced in this circuit (i.e., the state before measurement, see Supplementary Figure 1), we analyze the unitary $U_{a,N,m}$. This entails an analysis of the elementary gates in Section 1.1 as well as an analysis of the unitaries composing the unitary $U_{a,N,m}$ (in Sections 1.2 and 1.3). This will allow us to verify that $\mathcal{W}_{a,N}(e^{-iRP}Ш_1^{(R)}, \mathsf{GKP}, |0\rangle)$ implements what can be seen as a CV version of the quantum subroutine of Shor's algorithm, see Section 1.5.

1.1 Action of composite unitaries

In this section, we discuss the action of the composite unitaries introduced in Figure 2 of the main text. Specifically, we study their effect on position-eigenstates and explain how the corresponding action arises from the elementary operations introduced in Figure 1 of the main text. We also analyze the corresponding circuit size (i.e., the number of elementary gates used) and depth (i.e., the number of gate layers) in each case.

1.1.1 Scalar multiplication

Let $\alpha > 0$ be arbitrary. The multiplication operator M_α is defined in terms of the squeezing operator $S(z) = e^{iz(QP+PQ)/2}$ as

$$M_\alpha = S(-\log \alpha) \ .$$

The action of M_α on an element $\Psi \in L^2(\mathbb{R})$ is given by

$$(M_\alpha \Psi)(x) = \frac{1}{\sqrt{\alpha}} \Psi(x/\alpha) \qquad \text{for} \qquad x \in \mathbb{R} \ . \tag{3}$$

This implies that for $x \in \mathbb{R}$, the position-eigenstate $|x\rangle$ is transformed as

$$M_\alpha |x\rangle \propto |\alpha \cdot x\rangle \qquad \text{for any } x \in \mathbb{R} \ . \tag{4}$$

(Here and in the following we omit normalization constants since position-eigenstates are not normalizable in the first place. We note that these normalization constants are fixed by the action of the corresponding function being evaluated, i.e., scalar multiplication by α in this case, see Section 5.) In other words, the operator M_α acts as a scalar multiplication by α in position-space. We represent this as

$$|x\rangle \, -\!\boxed{M_\alpha}\!- \, |\alpha \cdot x\rangle \qquad \text{for} \qquad x \in \mathbb{R} \ .$$

We note that this action can equivalently be understood by the fact that squeezing is a Gaussian unitary, and the action on the mode operators (Q, P) in the Heisenberg picture is given by

$$\begin{aligned} M_\alpha^\dagger Q M_\alpha &= \alpha Q \\ M_\alpha^\dagger P M_\alpha &= \tfrac{1}{\alpha} P \ . \end{aligned} \tag{5}$$

Eqs. (3), (4) and (5) are all essentially equivalent expressions defining the action of M_α. We will use these different formalisms interchangeably, but will mostly rely on terms of the form (4).

We can find the following decomposition of M_α in terms of elementary operations. Let

$$\ell := \lceil |\log \alpha| \rceil \ .$$

We decompose unitary M_α as

$$M_\alpha = S(-(\log \alpha)/\ell)^\ell \ , \tag{6}$$

where the power ℓ indicates repeated application of ℓ single-mode squeezing operations $S(-(\log \alpha)/\ell)$ of a constant strength, i.e., the absolute value of the squeezing parameter is bounded by $|(\log \alpha)/\ell| \leq 1$. Therefore the unitary M_α can be implemented by a circuit of size and depth $\ell = O(|\log \alpha|)$ for $\alpha \to \infty$ (respectively $\alpha \to 0$).

1.1.2 Translation by R

Let $R \geq 1$. Recall that the unitary e^{-iRP} is Gaussian and acts as a translation in position-space, i.e.,

$$\begin{aligned} e^{iRP} Q e^{-iRP} &= Q + R \cdot I \\ e^{iRP} P e^{-iRP} &= P \end{aligned} \ , \tag{7}$$

where I denotes the identity on $L^2(\mathbb{R})$. We express this as

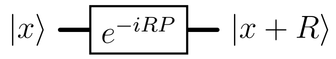

for $x \in \mathbb{R}$.

Combining (5) and (7), it is straightforward to verify that

$$e^{-iRP} = M_R \cdot e^{-iP} \cdot M_{1/R} \ . \tag{8}$$

By decomposing both M_R and $M_{1/R}$ as in Eq. (6), Eq. (8) implies that the unitary e^{-iRP} can be realized by a circuit of size and depth $O(\log R)$.

1.1.3 Qubit-controlled scalar multiplication

The qubit-controlled version of the unitary M_α is denoted $\mathsf{ctrl}M_\alpha$. It multiplies the position $x \in \mathbb{R}$ of a bosonic mode by a scalar $\alpha > 0$ if the control qubit is in the state $|1\rangle$. This operation is introduced in Figure 2 of the main text as

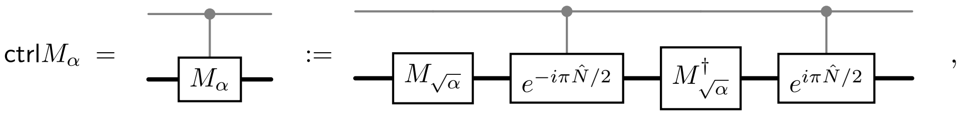

where $\hat{N} = (Q^2 + P^2 - I)/2$.

We can show that the unitary $\mathsf{ctrl}M_\alpha$ has the following action on a product state $|b\rangle \otimes |x\rangle$, where $|b\rangle \in \{|0\rangle, |1\rangle\}$ is a computational basis state of a qubit, and $|x\rangle$ for $x \in \mathbb{R}$ denotes the position-eigenstate: We have

$$\mathsf{ctrl}M_\alpha \left(|b\rangle \otimes |x\rangle\right) \propto \begin{cases} |0\rangle \otimes |x\rangle & \text{if } b = 0 \\ |1\rangle \otimes |\alpha \cdot x\rangle & \text{if } b = 1 \ . \end{cases}$$

We denote this as

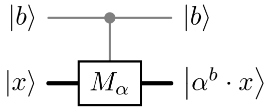

for $b \in \{0, 1\}$ and $x \in \mathbb{R}$.

This claim is trivial for $b = 0$. For $b = 1$, it follows from the identity

$$e^{-i\pi\hat{N}/2} M^\dagger_{\sqrt{\alpha}} e^{i\pi\hat{N}/2} = M_{\sqrt{\alpha}}$$

because the Gaussian unitary $e^{i\pi\hat{N}/2}$ acts as

$$\begin{aligned} e^{i\pi\hat{N}/2} Q e^{-i\pi\hat{N}/2} &= -P \\ e^{i\pi\hat{N}/2} P e^{-i\pi\hat{N}/2} &= Q \ . \end{aligned}$$

By definition, the circuit realizing $\mathsf{ctrl}M_\alpha$ is composed of two controlled $e^{\pm i\pi\hat{N}/2}$ gates and two unitaries $M_{\sqrt{\alpha}}$ and $M^\dagger_{\sqrt{\alpha}} = M_{1/\sqrt{\alpha}}$ (see Section 1.1.1). Therefore, both the circuit size as well as the circuit depth of the gate $\mathsf{ctrl}M_\alpha$ are dominated by that of $M_{\sqrt{\alpha}}$ and scale as $O(|\log \alpha|)$ for $\alpha \to \infty$ (respectively $\alpha \to 0$).

1.1.4 Coherent extraction of the least significant bit (LSB)

Let $x \in \mathbb{Z}$ be an integer. We define the least significant bit (LSB) of x as $x_0 = x \bmod 2$

The following circuit (see Figure 2 of the main text) defines a unitary $U^{\mathsf{LSB}}_{\mathrm{A}\to\mathrm{Q}}$ acting on mode A and qubit Q:

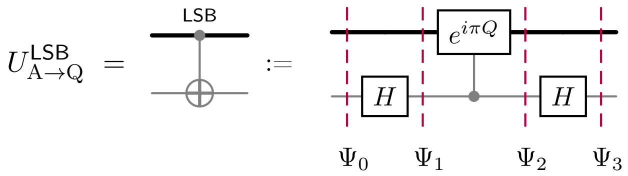

. (9)

We show that it extracts the LSB of the position of mode A into the qubit Q. That is, for any integer $x \in \mathbb{Z}$ encoded into the position-eigenstate $|x\rangle$ of a mode A, this unitary exactly computes the LSB x_0 of x into the qubit Q, i.e., we have

$$U^{\mathsf{LSB}}_{\mathrm{A}\to\mathrm{Q}}\left(|x\rangle_{\mathrm{A}} \otimes |b\rangle_Q\right) = |x\rangle_{\mathrm{A}} \otimes |x_0 \oplus b\rangle_{\mathrm{Q}} \qquad \text{for all } x \in \mathbb{Z} \text{ and } b \in \{0,1\}\ ,$$

where $\oplus$ denotes addition modulo 2.

Proof. We consider the intermediate states Ψ_0, Ψ_1 and Ψ_2, see (9). Starting from $|\Psi_0\rangle \propto |x\rangle \otimes |b\rangle$, we derive

$$\begin{aligned} |\Psi_1\rangle &\propto (I \otimes H)\,|\Psi_0\rangle \\ &\propto |x\rangle \otimes \big(\,|0\rangle + (-1)^b\,|1\rangle\,\big)\ ; \end{aligned}$$

hence

$$\begin{aligned} |\Psi_2\rangle &\propto \left(I \otimes I + e^{i\pi Q} \otimes |1\rangle\langle 1|\right)|\Psi_1\rangle \\ &\propto |x\rangle \otimes |0\rangle + (-1)^b e^{i\pi x}\,|x\rangle \otimes |1\rangle \\ &\propto |x\rangle \otimes |0\rangle + (-1)^b e^{i\pi x_0}\,|x\rangle \otimes |1\rangle \\ &\propto |x\rangle \otimes |0\rangle + (-1)^{x_0+b}\,|x\rangle \otimes |1\rangle\ . \end{aligned} \tag{10}$$

We note that Eq. (10) relies on the identity $e^{i\pi x} = (-1)^x = (-1)^{x_0}$ for any $x \in \mathbb{Z}$, i.e., the fact that the LSB x_0 determines whether or not x is even or odd.

It follows that the output state is

$$\begin{aligned} |\Psi_3\rangle &\propto (I \otimes H)\,|\Psi_2\rangle \\ &\propto |x\rangle \otimes |x_0 \oplus b\rangle\ , \end{aligned}$$

which is the claim. □

We will represent this as

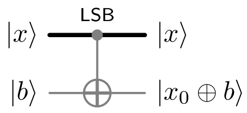

for $\quad x \in \mathbb{Z}$ and $b \in \{0,1\}$.

The unitary $U^{\mathsf{LSB}}_{\mathrm{A}\to\mathrm{Q}}$ is implemented by a circuit of a constant size and constant depth.

1.2 The unitary V_α

This building block V_α is used repeatedly (with $\alpha > 0$) in the definition of the unitary $V_{a,N,m}$ (see Figure 2 in the main text). It is defined as follows, where we labeled the respective bosonic modes by $\mathrm{A}, \mathrm{B}, \mathrm{C}$ and the qubit by Q.

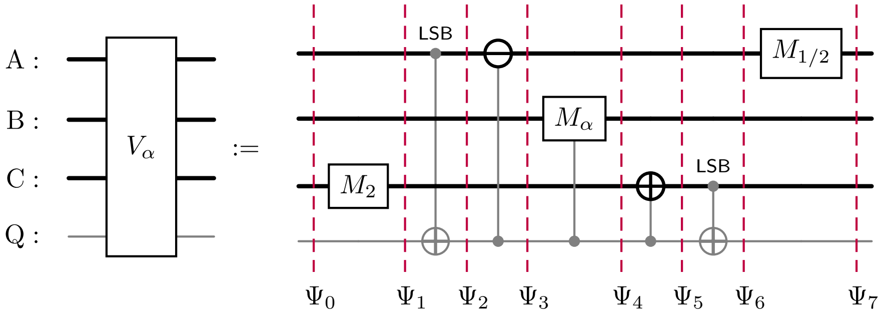

(11)

We show that this unitary acts on a tensor product of position-eigenstates $|x\rangle, |y\rangle, |z\rangle$ and of a qubit in the state $|0\rangle$ as

$$V_\alpha\big(|x\rangle_{\mathrm{A}} \otimes |y\rangle_{\mathrm{B}} \otimes |z\rangle_{\mathrm{C}} \otimes |0\rangle_{\mathrm{Q}}\big) \;\propto\; |(x-x_0)/2\rangle_{\mathrm{A}} \otimes |\alpha^{x_0} \cdot y\rangle_{\mathrm{B}} \otimes |2z + x_0\rangle_{\mathrm{C}} \otimes |0\rangle_{\mathrm{Q}} \tag{12}$$

for all $x \in \mathbb{Z}$, $y \in \mathbb{R}$ and $z \in \mathbb{Z}$.

Proof. The unitary V_α is the result of applying a sequence of 7 unitaries, see the illustration (11) for the definition of intermediate states $\Psi_0, \ldots, \Psi_7$.

Starting with the initial state

$$|\Psi_0\rangle \propto |x\rangle \otimes |y\rangle \otimes |z\rangle \otimes |0\rangle \;\;,$$

we first squeeze mode C, obtaining

$$\begin{aligned} |\Psi_1\rangle &\propto (I \otimes I \otimes M_2 \otimes I)\Psi_0 \\ &\propto |x\rangle \otimes |y\rangle \otimes |2z\rangle \otimes |0\rangle \;\;. \end{aligned}$$

We then extract the LSB x_0 of the position x of mode A to the qubit, resulting in

$$\begin{aligned} |\Psi_2\rangle &\propto U^{\mathsf{LSB}}_{\mathrm{A}\to\mathrm{Q}} |\Psi_1\rangle \\ &\propto |x\rangle \otimes |y\rangle \otimes |2z\rangle \otimes |x_0\rangle \;\;. \end{aligned}$$

Having extracted the LSB x_0 to the qubit, we can now subtract x_0 from the position-eigenstate of mode A by applying a qubit-controlled translation, resulting in

$$\begin{aligned} |\Psi_3\rangle &\propto \big(I \otimes I \otimes I \otimes |0\rangle\langle 0| + e^{iP} \otimes I \otimes I \otimes |1\rangle\langle 1|\big)\,|\Psi_2\rangle \\ &\propto |x - x_0\rangle \otimes |y\rangle \otimes |2z\rangle \otimes |x_0\rangle \;\;. \end{aligned}$$

After this step, the position $x - x_0$ of mode A is even. We then use x_0 (stored in the qubit Q) as a control to multiply the position-eigenstate of mode B by α, giving

$$\begin{aligned} |\Psi_4\rangle &\propto \big(I \otimes I \otimes I \otimes |0\rangle\langle 0| + I \otimes M_\alpha \otimes I \otimes |1\rangle\langle 1|\big)\,|\Psi_3\rangle \\ &\propto |x - x_0\rangle \otimes |\alpha^{x_0} \cdot y\rangle \otimes |2z\rangle \otimes |x_0\rangle \;\;, \end{aligned}$$

and add x_0 to the position of mode C by a qubit-controlled translation. This results in

$$\begin{aligned}|\Psi_5\rangle &\propto \left(I\otimes I\otimes I\otimes |0\rangle\langle 0| + I\otimes I\otimes e^{-iP}\otimes |1\rangle\langle 1|\right)|\Psi_4\rangle \\ &\propto |x-x_0\rangle\otimes|\alpha^{x_0}\cdot y\rangle\otimes|2z+x_0\rangle\otimes|x_0\rangle \ .\end{aligned}$$

We then restore the qubit Q to the computational basis state $|0\rangle$ by application of the unitary $U^{\mathsf{LSB}}_{\mathrm{C}\to\mathrm{Q}}$, that is,

$$\begin{aligned}|\Psi_6\rangle &\propto U^{\mathsf{LSB}}_{\mathrm{C}\to\mathrm{Q}}|\Psi_5\rangle \\ &\propto |x-x_0\rangle\otimes|\alpha^{x_0}\cdot y\rangle\otimes|2z+x_0\rangle\otimes|0\rangle \ .\end{aligned}$$

Here we used that $2z+x_0$ has LSB x_0. In the final step, we apply squeezing to mode A, obtaining

$$\begin{aligned}|\Psi_7\rangle &\propto \left(M_{1/2}\otimes I\otimes I\otimes I\right)|\Psi_6\rangle \\ &\propto |(x-x_0)/2\rangle\otimes|\alpha^{x_0}\cdot y\rangle\otimes|2z+x_0\rangle\otimes|0\rangle \ .\end{aligned}$$

This proves Eq. (12). □

To summarize, the unitary V_α acts as

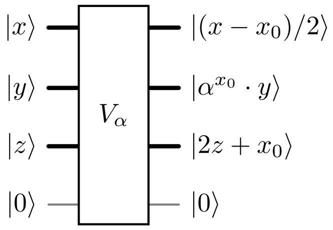

for $x\in\mathbb{Z}$, $y\in\mathbb{R}$ and $z\in\mathbb{Z}$.

Furthermore, both the size as well as the depth of the circuit implementing the unitary V_α scale as $O(|\log\alpha|)$ for $\alpha\to 0$ respectively $\alpha\to\infty$. Indeed, these quantities are dominated by the circuit size and depth of the unitary $\mathsf{ctrl}M_\alpha$, whereas the other gates only contribute a constant.

1.3 Controlled multiplication by a pseudomodular power

At the core of the circuit $\mathcal{W}_{a,N}(e^{-iRP}\text{Ш}_1^{(R)}, \mathsf{GKP}, |0\rangle)$ is the composed unitary $V_{a,N,m}$. It is defined by iterative application of the unitaries V_α with changing parameter α as follows (cf. Figure 2 of the main text).

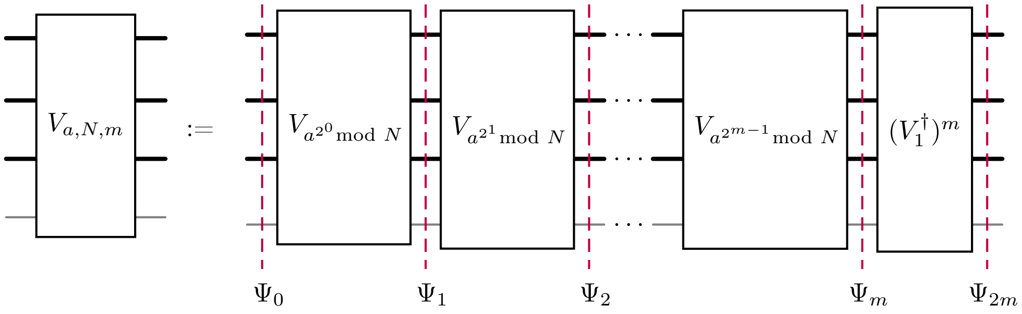

(13)

Since each V_α is given by a sequence of elementary gates (see Eq. (11)) and this set is closed under taking inverses, the unitary $V_{a,N,m}$ can be decomposed into elementary gates.

We will argue that the unitary $V_{a,N,m}$ multiplies the position y of the mode B by the value $f_{a,N,m}(x)$ where $x \in \mathbb{N}_0$ is the position of the mode A. Here

$$f_{a,N,m}(x) = \prod_{i=0}^{m-1} \left(a^{2^i} \mod N\right)^{x_i} , \tag{14}$$

where we used the m least significant bits in the binary representation of $x \in \mathbb{N}_0$

$$x = \sum_{j=0}^{\infty} x_j 2^j .$$

We call the function $f_{a,N,m}(x)$ the pseudomodular power. In particular, we have $a^x = a^{\sum_{i=0}^{m-1} x_i 2^i} = \prod_{i=0}^{m-1} \left(a^{2^i}\right)^{x_i}$ for any integer $x \in \{0, \ldots, 2^m - 1\}$ and thus thus

$$f_{a,N,m}(x) \equiv a^x \pmod N \qquad \text{for all } x \in \{0, \ldots, 2^m - 1\} . \tag{15}$$

We show the following action of $V_{a,N,m}$ for all $x \in \mathbb{N}_0$, $y \in \mathbb{R}$ and $z \in \mathbb{N}_0$. We have

$$V_{a,N,m}\big(|x\rangle \otimes |y\rangle \otimes |z\rangle \otimes |0\rangle \big) \ \propto \ |x\rangle \otimes |f_{a,N,m}(x) \cdot y\rangle \otimes |z\rangle \otimes |0\rangle . \tag{16}$$

Proof. Let $x \in \mathbb{N}_0$, $y \in \mathbb{R}$ and $z \in \mathbb{N}_0$. Consider the initial state

$$|\Psi_0\rangle \propto |x\rangle \otimes |y\rangle \otimes |z\rangle \otimes |0\rangle ,$$

see diagram (13).

Each element $(V_{a^{2^i} \mod N})_{i\in\{0,\ldots,m-1\}}$ of the first m gates defining $V_{a,N,m}$ corresponds to a bit-controlled multiplication by a factor $(a^{2^i} \mod)^{x_i}$ in the definition (14) of the pseudomodular power.

In more detail, the application of $V_{a^{2^0} \mod N}$ to Ψ_0 multiplies the position of the mode B by the factor $(a^{2^0} \mod N)^{x_0}$ and moves x_0 (originally the LSB of the the position x in mode A) to the LSB of the position in mode C, that is,

$$\begin{aligned} |\Psi_1\rangle &\propto V_{a^{2^0} \mod N} |\Psi_0\rangle \\ &\propto |(x - x_0)/2\rangle \otimes \left|(a^{2^0} \mod N)^{x_0} \cdot y\right\rangle \otimes |2z + x_0\rangle \otimes |0\rangle . \end{aligned}$$

Subsequently, $V_{a^{2^1} \mod N}$ is applied to Ψ_1. This multiplies the position of mode B by the second factor $(a^{2^1} \mod N)^{x_1}$ of the pseudomodular power and moves x_1 (previously the LSB of the position $(x - x_0)/2$ of the mode A) to the LSB of the position in the mode C, i.e.,

$$\begin{aligned} &|\Psi_2\rangle \propto V_{a^{2^1} \mod N} |\Psi_1\rangle \\ &\quad \propto \left|(x - 2^1 x_1 - 2^0 x_0)/2^2\right\rangle \otimes \left|(a^{2^1} \mod N)^{x_1} \cdot (a^{2^0} \mod N)^{x_0} \cdot y\right\rangle \otimes \left|2^2 z + 2^1 x_0 + 2^0 x_1\right\rangle \otimes |b\rangle . \end{aligned}$$

Note that x_0 is now in the second-least-significant bit of mode C). Inductively, we obtain for all $x \in \mathbb{N}_0$, $y \in \mathbb{R}$ and $z \in \mathbb{N}_0$ that after applying the jth unitary $V_{a^{2^{j-1}} \mod N}$ in the sequence, we have the state

$$|\Psi_j\rangle \propto \left|x^{(j)}\right\rangle \otimes \left|y^{(j)}\right\rangle \otimes \left|z^{(j)}\right\rangle \otimes |0\rangle ,$$

where

$$\begin{aligned}
x^{(j)} &= 2^{-j}x - \sum_{i=0}^{j-1} 2^{-j+i}x_i \\
y^{(j)} &= \left(\prod_{i=0}^{j-1} \left(a^{2^i} \mod N\right)^{x_i}\right) \cdot y \\
z^{(j)} &= 2^j z + \sum_{i=0}^{j-1} 2^{j-1-i}x_i \ .
\end{aligned} \tag{17}$$

Thus applying the first m gates, the resulting state is

$$\begin{aligned}
|\Psi_m\rangle &\propto \left|x^{(m)}\right\rangle \otimes \left|y^{(m)}\right\rangle \otimes \left|z^{(m)}\right\rangle \otimes |0\rangle \\
&= \left|x^{(m)}\right\rangle \otimes |f_{a,N,m}(x) \cdot y\rangle \otimes \left|z^{(m)}\right\rangle \otimes |0\rangle \ ,
\end{aligned}$$

where we used the definition (14) of the pseudomodular power.

At this stage, the multiplication of the position y of the mode B by the pseudomodular power $f_{a,N,m}(x)$ of the position x of the mode A is completed. However, x (or more precisely the m lowest bits of x) has been moved from mode A to mode C (in the reversed binary representation). The remaining steps (the m-fold application of $V_1^\dagger$) undo that latter, undesired operation.

Since $(x, y, z) \mapsto (2x + z_0, y, (z - z_0)/2)$ is a bijection on $\mathbb{N}_0 \times \mathbb{R} \times \mathbb{N}_0$ which is computed by the unitary V_1, it is easy to verify that

$$V_1^\dagger \left(|x\rangle \otimes |y\rangle \otimes |z\rangle \otimes |0\rangle\right) = |2x + z_0\rangle \otimes |y\rangle \otimes |(z - z_0)/2\rangle \otimes |0\rangle$$

for all $x \in \mathbb{N}_0$, $y \in \mathbb{R}$ and $z \in \mathbb{N}_0$. Applying the unitary $V_1^\dagger$ to $\Psi^{(m)}$ gives

$$V_1^\dagger \Psi_m \propto \left|2x^{(m)} + z_0^{(m)}\right\rangle \otimes \left|f_{a,N,m}(x) \cdot y\right\rangle \otimes \left|(z^{(m)} - z_0^{(m)})/2\right\rangle \otimes |0\rangle \ . \tag{18}$$

By definition of $z^{(m)}$ we have that $z_0^{(m)} = x_{m-1}$, thus

$$\begin{aligned}
2x^{(m)} + z_0^{(m)} &= 2\left(2^{-m}x - \sum_{i=0}^{m-1} 2^{-m+i}x_i\right) + x_{m-1} \\
&= 2^{-(m-1)}x - \sum_{i=0}^{(m-1)-1} 2^{-(m-1)+i}x_i \\
&= x^{(m-1)} \ ,
\end{aligned}$$

where we used definition of $x^{(j)}$ from (17). Similarly, we have

$$\begin{aligned}
(z^{(m)} - z_0^{(m)})/2 &= \left(\left(2^m z + \sum_{i=0}^{m-1} 2^{m-1-i}x_i\right) - x_{m-1}\right)/2 \\
&= 2^{m-1}z + \sum_{i=0}^{(m-1)-1} 2^{(m-1)-1-i}x_i \\
&= z^{(m-1)} \ .
\end{aligned}$$

Inserting these into (18) we obtain

$$V_1^\dagger \left|\Psi_m\right\rangle \propto \left|x^{(m-1)}\right\rangle \otimes \left|f_{a,N,m}(x)\cdot y\right\rangle \otimes \left|z^{(m-1)}\right\rangle \otimes |0\rangle \ .$$

By induction, it follows that

$$(V_1^\dagger)^m \left|\Psi_m\right\rangle \propto |x\rangle \otimes \left|f_{a,N,m}(x)\cdot y\right\rangle \otimes |z\rangle \otimes |0\rangle$$

for all $x \in \mathbb{N}_0$, $y \in \mathbb{R}$ and $z \in \mathbb{N}_0$. This completes the proof. □

We summarize this action as

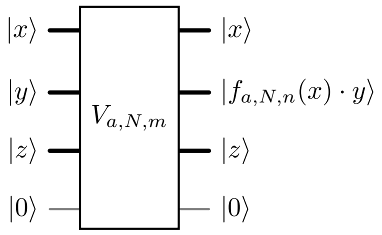

for $\quad x \in \mathbb{N}_0$, $y \in \mathbb{R}$ and $z \in \mathbb{N}_0$.

By definition, $V_{a,N,m}$ is composed of m gates V_α with $0 < \alpha < N$ and m gates $V_1^\dagger$. Therefore, both the size as well as the depth of the unitary $V_{a,N,m}$ scale as $O(m \log N)$.

1.4 Controlled translation by a pseudomodular power

The unitary $U_{a,N,m} = V^\dagger_{a,N,m} \cdot (I \otimes e^{-iP} \otimes I \otimes I) \cdot V_{a,N,m}$ translates the position y of the mode B by $f_{a,N,m}(x)$, where x is the position of the mode A. Diagrammatically it is defined as

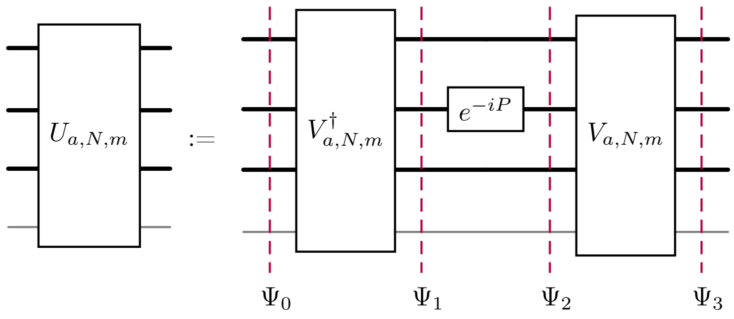

. (19)

We show that the unitary $U_{a,N,m}$ acts as

$$U_{a,N,m}\left(|x\rangle \otimes |y\rangle \otimes |z\rangle \otimes |0\rangle\right) \propto |x\rangle \otimes |y + f_{a,N,m}(x)\rangle \otimes |z\rangle \otimes |0\rangle \tag{20}$$

for all $x \in \mathbb{N}_0$, $y \in \mathbb{R}$ and $z \in \mathbb{N}_0$.

Proof. We first note that (16) implies

$$V^\dagger_{a,N,m}\left(|x\rangle \otimes |y\rangle \otimes |z\rangle \otimes |0\rangle\right) = |x\rangle \otimes |y/f_{a,N,m}(x)\rangle \otimes |z\rangle \otimes |0\rangle$$

for all $x \in \mathbb{N}_0$, $y \in \mathbb{R}$, and $z \in \mathbb{N}_0$.

Thus starting from $|\Psi_0\rangle \propto |x\rangle \otimes |y\rangle \otimes |z\rangle \otimes |0\rangle$, we obtain (see Eq. (19))

$$\begin{aligned}
|\Psi_1\rangle &\propto (V_{a,N,m})^\dagger |\Psi_0\rangle \\
&\propto |x\rangle \otimes |y/f_{a,N,m}(x)\rangle \otimes |z\rangle \otimes |0\rangle \ , \\
|\Psi_2\rangle &\propto (I \otimes e^{-iP} \otimes I \otimes I)\, |\Psi_1\rangle \\
&\propto |x\rangle \otimes |1 + y/f_{a,N,m}(x)\rangle \otimes |z\rangle \otimes |0\rangle \ , \\
|\Psi_3\rangle &\propto (V_{a,N,m})\, |\Psi_2\rangle \\
&\propto |x\rangle \otimes |y + f_{a,N,m}(x)\rangle \otimes |z\rangle \otimes |0\rangle \ ,
\end{aligned}$$

which is the claim. $\square$

We summarize this as

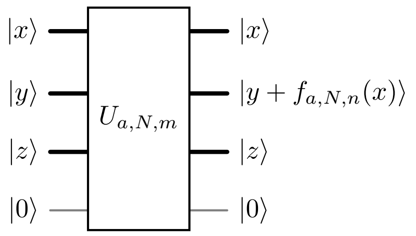

for $\quad x \in \mathbb{N}_0,\ y \in \mathbb{R}$ and $z \in \mathbb{N}_0$.

Since both $V_{a,N,m}^{\dagger}$ and $V_{a,N,m}$ can be realized by a circuit of size $O(m \log N)$ and e^{-iP} belongs to our gate set, both the circuit size as well as the circuit depth of the unitary $U_{a,N,m}$ scale as $O(m \log N)$.

1.5 Analysis of the circuit $\mathcal{W}_{a,N}(e^{-iRP}Ш_1^{(R)}, \mathsf{GKP}, |0\rangle)$

We now explain the intuition behind the circuit $\mathcal{Q}_{a,N}$ (see Figure 3 of the main text) by analyzing its idealized version $\mathcal{W}_{a,N}(e^{-iRP}Ш_1^{(R)}, \mathsf{GKP}, |0\rangle)$ (see Supplementary Figure 1). We show that the circuit $\mathcal{W}_{a,N}(e^{-iRP}Ш_1^{(R)}, \mathsf{GKP}, |0\rangle)$ implements a form of (pseudo)modular exponentiation and a modular measurement.

Note that a rigorous analysis of circuit $\mathcal{Q}_{a,N}$ is provided in Section 2. The following analysis of the circuit $\mathcal{W}_{a,N}(e^{-iRP}Ш_1^{(R)}, \mathsf{GKP}, |0\rangle)$ is for illustrative purposes only.

Pseudomodular exponentiation. We analyze the action of the circuit $\mathcal{W}_{a,N}(e^{-iRP}Ш_1^{(R)}, \mathsf{GKP}, |0\rangle)$. Recall that the initial state is (see Supplementary Figure 1)

$$\begin{aligned}|\Psi_0\rangle &\propto e^{-iRP}|Ш_1^{(R)}\rangle \otimes |\mathsf{GKP}\rangle \otimes |0\rangle \otimes |0\rangle \\ &= \left(\sum_{x=0}^{2R-1} |x\rangle\right) \otimes \left(\sum_{y\in\mathbb{Z}} |y\rangle\right) \otimes |0\rangle \otimes |0\rangle \ ,\end{aligned}$$

where $R = 2^{17n-1} = 2^{m-1}$ (see Supplementary Table 1). Thus the state Ψ_1 is

$$\begin{aligned}|\Psi_1\rangle &\propto (I \otimes M_N \otimes I \otimes I)\ |\Psi_0\rangle \\ &\propto (I \otimes M_N \otimes I \otimes I) \left(\sum_{x=0}^{2R-1} |x\rangle\right) \otimes \left(\sum_{y\in\mathbb{Z}} |y\rangle\right) \otimes |0\rangle \otimes |0\rangle \\ &\propto \left(\sum_{x=0}^{2R-1} |x\rangle\right) \otimes \left(\sum_{y\in\mathbb{Z}} |N \cdot y\rangle\right) \otimes |0\rangle \otimes |0\rangle\end{aligned}$$

Subsequently, we apply the unitary $U_{a,N,m}$. Following Eq. (20) we obtain

$$\begin{aligned}|\Psi_2\rangle &\propto U_{a,N,m}\ |\Psi_1\rangle \\ &\propto \sum_{x=0}^{2R-1} \sum_{y\in\mathbb{Z}} |x\rangle \otimes |N \cdot y + f_{\alpha,N,m}(x)\rangle \otimes |0\rangle \otimes |0\rangle \ .\end{aligned}$$

Modular measurement. Let us trace out both systems C and Q. At this point, measuring the mode B implements a form of modular measurement of the position of mode A, in our case a (mod N)-measurement. Suppose we measure the mode B and obtain the measurement outcome

$k \in \mathbb{Z}$. This implies that every $x \in \{0, \ldots 2R-1\}$ contained in the support of the reduced state of the mode A has to satisfy

$$N \cdot y + f_{\alpha,N,m}(x) = k \qquad \text{for some } y \in \mathbb{Z} \ .$$

In particular

$$\begin{aligned} k &\equiv f_{a,N,m}(x) \pmod N \\ &\equiv a^x \pmod N \ , \end{aligned}$$

where we obtained the last equality by (15). Thus after obtaining the measurement outcome k in mode B, the reduced state $\Psi_2^{(k)}$ of mode A is

$$\left|\Psi_2^{(k)}\right\rangle \propto \sum_{\substack{x\in\{0,\ldots,2R-1\}: \\ a^x \equiv k \mod N}} |x\rangle \ .$$

This state is formally similar to the state produced by the quantum subroutine of Shor's algorithm, but with position-encoded integers.

The size (i.e., number of elementary operations) of the circuit $\mathcal{W}_{a,N}(e^{-iRP}\text{Ш}_1^{(R)}, \mathsf{GKP}, |0\rangle)$ excluding the preparation of the initial states $(e^{-iRP}\text{Ш}_1^{(R)}, \mathsf{GKP}, |0\rangle)$ is the size of the composed unitaries M_N and $U_{a,N,m}$, plus the P-quadrature measurement. With the parameters (m, R) given in Supplementary Table 1 this adds up to $O(\log N) + O(n^2) + 1 = O(n^2)$ elementary operations. Ignoring the initial state preparation, the circuit $\mathcal{W}_{a,N}(e^{-iRP}\text{Ш}_1^{(R)}, \mathsf{GKP}, |0\rangle)$ is thus composed of $O(n^2)$ elementary operations (of the form (i)–(iv) in the main text).

2 Circuit analysis with finitely squeezed GKP states

In this section, we prove that the circuit $\mathcal{Q}_{a,N}$ (cf. Figure 3 in the main text) can be used to factor integers even though it uses approximate (instead of ideal) GKP states and finitely squeezed Gaussian states (instead of position-eigenstates). We use that this remains true even if the circuit is initialized with states prepared by our GKP state preparation protocol $\mathcal{P}^{\mathsf{GKP}}_{\kappa,\Delta}$ introduced in [2].

We first give the proof of Lemma 1 (of the main text) which we restate here for convenience:

Lemma 1. *Suppose N is an n-bit number. There is a polynomial-time classical algorithm which—given a uniformly chosen element $a \sim \mathbb{Z}_N^*$ and a single sample from the output distribution of the circuit $\mathcal{Q}_{a,N}$ (see Figure 3 of the main text)—produces a factor of N with probability at least $\Omega(1/\log n)$.*

Finally, we prove that combined with the GKP state preparation protocol from [2] and repetition of our algorithm leads to the Theorem 1 (of the main text), which we restate here.

Theorem 1 (Efficient quantum algorithm for factoring)**.** *There is a polynomial-time algorithm which, given an n-bit integer N,*

(I) repeatedly uses a quantum circuit on 3 oscillators and 1 qubit consisting of $O(n^2)$ elementary operations (i)– (iv) *of the main text, and*

(II) produces a factor of N with constant probability.

We first discuss relevant definitions of approximate GKP states in Section 2.1. In Section 2.2, we give the proof of Lemma 1 and Theorem 1. The proof of various auxiliary results is deferred to subsequent sections.

2.1 Definition of approximate GKP states

We define approximate position-eigenstates as well as approximate GKP states in this section and summarize some of their properties.

Let us start with approximate position-eigenstates. For $z \in \mathbb{R}$ let $|\chi_\Delta(z)\rangle$ be an approximate position-eigenstate defined as

$$|\chi_\Delta(z)\rangle := e^{-iPz} S(\log 1/\Delta) |\mathrm{vac}\rangle \ ,$$

where $S(\xi) = e^{i\frac{\xi}{2}(QP+PQ)}$ is the single-mode squeezing operation. By definition, $\chi_\Delta(z) \in L^2(\mathbb{R})$ is the function

$$(\chi_\Delta(z))(x) := \Psi_\Delta(x-z) \ ,$$

where Ψ_Δ is the centered Gaussian given by

$$\Psi_\Delta(x) = \frac{1}{(\pi\Delta^2)^{1/4}} e^{-x^2/(2\Delta^2)} \qquad \text{for} \qquad x \in \mathbb{R}.$$

Notice that $|\Psi_\Delta(\cdot)|^2$ is a centered normal distribution with variance $\sigma^2 = \Delta^2/2$. The vacuum state $|\mathrm{vac}\rangle$ is defined as Ψ_Δ where $\Delta = 1$.

We define the approximate GKP state

$$|\mathsf{GKP}_{\kappa,\Delta}\rangle := C_{\kappa,\Delta} \sum_{z\in\mathbb{Z}} \eta_\kappa(z)\, |\chi_\Delta(z)\rangle \tag{21}$$

where $C_{\kappa,\Delta} \in \mathbb{R}$ is a normalization constant, and $\eta_\kappa(\cdot)$ a Gaussian envelope specified by a Gaussian distribution with variance $\sigma^2 = \frac{1}{2\kappa^2}$, i.e., for $\kappa > 0$

$$\eta_\kappa(z) = \frac{\sqrt{\kappa}}{\pi^{1/4}} e^{-\kappa^2 z^2/2} \ , \tag{22}$$

for illustration see Supplementary Figure 2a. We note that our definition (21) is slightly different from the convention in literature, see e.g. [3], where a non-integer spacing is used.

For our analysis it is convenient to define truncated approximate GKP states. This will ensure certain orthogonality properties. To this end let us define the $\pm\varepsilon$-supported position-eigenstate $|\chi^\varepsilon_\Delta(z)\rangle$ as a truncated Gaussian having support on $[z-\varepsilon, z+\varepsilon]$ for some small $\varepsilon \in (0, 1/2)$. More precisely, $\chi^\varepsilon_\Delta(z) \in L^2(\mathbb{R})$ is defined as

$$(\chi^\varepsilon_\Delta(z))(x) := \Psi^\varepsilon_\Delta(x-z) \qquad \text{for} \qquad x \in \mathbb{R} \ ,$$

where

$$\Psi^\varepsilon_\Delta = \frac{\Pi_{[-\varepsilon,\varepsilon]}\Psi_\Delta}{\left\|\Pi_{[-\varepsilon,\varepsilon]}\Psi_\Delta\right\|} \ ,$$

where we denote by $\Pi_{\mathcal{C}}$ the orthogonal projection onto the subspace of $L^2(\mathbb{R})$ of functions supported on $\mathcal{C} \subseteq \mathbb{R}$. Since $\varepsilon < 1/2$ the $\pm\varepsilon$-supported position-eigenstates satisfy the orthogonality property

$$\langle \chi^\varepsilon_\Delta(z), \chi^\varepsilon_\Delta(z')\rangle = \delta_{z,z'} \qquad \text{for all} \qquad z, z' \in \mathbb{Z} \ .$$

Furthermore, the overlap between a $\pm\varepsilon$-supported position-eigenstate and a displaced vacuum state centered at z is close to 1 for Δ small and $\varepsilon \in [\sqrt{\Delta}, 1/2)$, see Lemma 7.1

$$|\langle \Psi_\Delta, \Psi^\varepsilon_\Delta\rangle|^2 \geq 1 - 2\Delta \ .$$

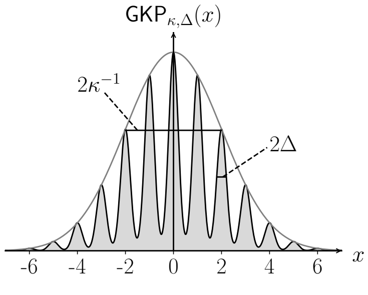

(a) The state $|\mathsf{GKP}_{\kappa,\Delta}\rangle \in L^2(\mathbb{R})$.

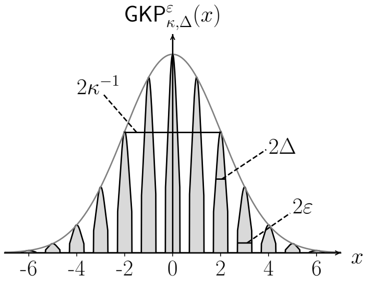

(b) The state $\left|\mathsf{GKP}^\varepsilon_{\kappa,\Delta}\right\rangle \in L^2(\mathbb{R})$.

Supplementary Figure 2. The approximate GKP state and its "truncated" version represented in position space.

We define the approximate GKP state $\left|\mathsf{GKP}^\varepsilon_{\kappa,\Delta}\right\rangle$ with truncated peaks as

$$\left|\mathsf{GKP}^\varepsilon_{\kappa,\Delta}\right\rangle := C_\kappa \sum_{z\in\mathbb{Z}} \eta_\kappa(z)\, |\chi^\varepsilon_\Delta(z)\rangle \ , \tag{23}$$

where C_κ is the normalization factor. A state $\left|\mathsf{GKP}^{\varepsilon}_{\kappa,\Delta}\right\rangle$ is shown in Supplementary Figure 2b. The GKP state with truncated peaks is related to its "non-truncated" version by

$$\mathsf{GKP}^{\varepsilon}_{\kappa,\Delta} = \frac{\Pi_{\mathbb{Z}(\varepsilon)}\mathsf{GKP}_{\kappa,\Delta}}{\left\|\Pi_{\mathbb{Z}(\varepsilon)}\mathsf{GKP}_{\kappa,\Delta}\right\|} ,$$

where $\mathbb{Z}(\varepsilon) = \mathbb{Z} + [-\varepsilon, \varepsilon]$ where the addition is to be understood as a Minkowski sum.

2.2 Proof of Lemma 1 and Theorem 1: Efficient quantum algorithm for factoring

We first provide a sufficient condition for when samples of a real-valued random variable provide a means for factoring. This statement is a straightforward generalization of the analysis of the classical post-processing procedure in Shor's algorithm. Recall that $\mathbb{Z}_N = \{0, \ldots, N-1\}$ is the set of elements in the ring of integers modulo N and $\mathbb{Z}_N^* = \{a \in \mathbb{Z}_N \mid \gcd(a, N) = 1\}$ is the set of elements of the multiplicative group of integers modulo N. We denote the order of a in the group $\mathbb{Z}_N^*$ by $r(a)$, i.e., this is the smallest positive integer r such that $a^r \equiv 1 \bmod N$.

The sufficient condition for a family $\{p_a\}_{a\in\mathbb{Z}_N^*}$ of probability density functions on $\mathbb{R}$ is expressed by the following definition.

Definition 2.1. *Let $N \in \mathbb{N}$ be an integer. Let $q = \min\{2^k \mid k \in \mathbb{N}, N^2 < 2^k\}$ be the smallest power of 2 greater than N^2. Let $a \in \mathbb{Z}_N^*$, and let $r(a)$ denote the order of $a \in \mathbb{Z}_N^*$. For $d \in \mathbb{Z}_{r(a)}$ define the union of intervals*

$$\Gamma_d(a) := \bigcup_{j\in\mathbb{Z}} \left[j + \frac{d}{r(a)} - \frac{1}{2q}, j + \frac{d}{r(a)} + \frac{1}{2q} \right] . \tag{24}$$

A family $\{p_a \mid p_a : \mathbb{R} \to [0, \infty)\}_{a\in\mathbb{Z}_N^*}$ of probability density functions on $\mathbb{R}$ is called suitable *if*

$$\min_{d\in\mathbb{Z}_r} \int_{\Gamma_d(a)} p_a(w)dw \geq \Omega(1) \cdot \frac{1}{r(a)} \qquad \text{for all} \qquad a \in \mathbb{Z}_N^* .$$

We depict the unions of intervals $\Gamma_d(a)$ in Supplementary Figure 3.

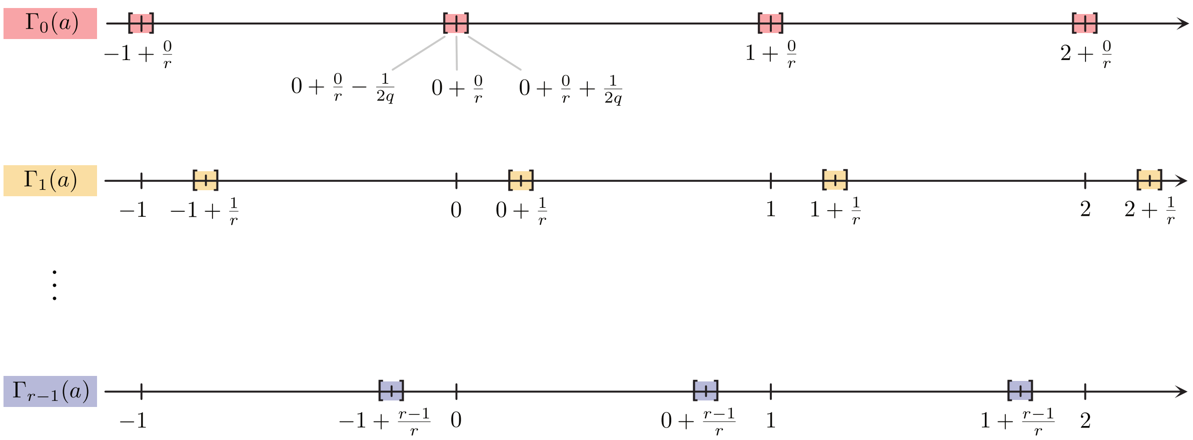

Supplementary Figure 3. An illustration of the union $\Gamma_d(a)$ of intervals from Definition 2.1, for $d \in \{0, \ldots, r-1\}$ depicted on the real axis. We shaded the intervals belonging to the same set $\Gamma_d(a)$ by the same color.

The following proposition shows that — as suggested by the terminology — suitable families of distributions can be used to factor.

Proposition 1 (Classical post-processing subroutine)**.** *There is a polynomial-time classical algorithm $\mathcal{A}$ such that the following holds. Given an n-bit integer $N \in \mathbb{N}$, assume that a pair (x, a) is generated as follows:*

(i) the element $a \sim U_{\mathbb{Z}_N^*}$ is drawn from the uniform distribution $U_{\mathbb{Z}_N^*}$ on $\mathbb{Z}_N^*$, and

(ii) $x \sim p_a$ is drawn from p_a, where $\{p_a \mid p_a : \mathbb{R} \to [0, \infty)\}_{a \in \mathbb{Z}_N^*}$ is a suitable family of probability density functions (see Definition 2.1).

Then the algorithm $\mathcal{A}$ — on input (x, a, N) — outputs a factor of N with probability at least

$$\Pr_{\substack{a \sim U_{\mathbb{Z}_N^*} \\ x \sim p_a}} [\mathcal{A}(x, a, N) \text{ divides } N] \geq \Omega\left(\frac{1}{\log n}\right) .$$

The runtime of $\mathcal{A}$ is polynomial in n.

The remainder of the proof of Lemma 1 establishes that the output distributions of the circuit $\mathcal{Q}_{a,N} = \mathcal{W}_{a,N}(e^{-iRP}\mathsf{GKP}_{\kappa_{\mathrm{A}},\Delta_{\mathrm{A}}}, \mathsf{GKP}_{\kappa_{\mathrm{B}},\Delta_{\mathrm{B}}}, \Psi_{\Delta_{\mathrm{C}}})$ in Figure 3 of the main text (for different $a \in \mathbb{Z}_N^*$) are suitable, i.e., satisfy Definition 2.1 and thus can be used for factoring according to Proposition 1. It consists of two parts summarized by Propositions 2 and 3 below.

First, we consider the output state of the circuit $\mathcal{Q}_{a,N}$ in Figure 3 of the main text before the measurement. It can be characterized as follows. For convenience, we use the shorthand notation $\|\Psi - \Phi\|_1 := \||\Psi\rangle\langle\Psi| - |\Phi\rangle\langle\Phi|\|_1$ for the variational distance between two rank-1-projections corresponding to pure states $\Psi, \Phi \in L^2(\mathbb{R})$.

Proposition 2 (Output state of the circuit $\mathcal{Q}_{a,N}$)**.** *Let $N \geq 8$ (i.e., $n \geq 4$) and $a \in \mathbb{Z}_N^*$. Let $(m, R, \kappa_{\mathrm{A}}, \Delta_{\mathrm{A}}, \kappa_{\mathrm{B}}, \Delta_{\mathrm{B}}, \Delta_{\mathrm{C}})$ be as specified in Supplementary Table 1. Let Ψ_a be the final state before the measurement of the circuit $\mathcal{Q}_{a,N}$ in Figure 3 of the main text. Let Φ_a be the state*

$$|\Phi_a\rangle = c_{\Phi_a} \sum_{z \in \mathbb{Z}} \eta_{\kappa_{\mathrm{A}}}(z - R) |\chi_{\Delta_{\mathrm{A}}}(z)\rangle \otimes e^{-i(a^z \mod N)P_{\mathrm{B}}} M_N \left|\mathsf{GKP}^{\varepsilon_{\mathrm{B}}}_{\kappa_{\mathrm{B}},\Delta_{\mathrm{B}}}\right\rangle \otimes \left|\Psi^{\varepsilon_{\mathrm{C}}}_{\Delta_{\mathrm{C}}}\right\rangle \otimes |0\rangle \ , \quad (25)$$

where c_{Φ_a} is a normalization constant and where $\varepsilon_{\mathrm{B}} = \sqrt{\Delta_{\mathrm{B}}}$ and $\varepsilon_{\mathrm{C}} = \sqrt{\Delta_{\mathrm{C}}}$. Then

$$\|\Psi_a - \Phi_a\|_1 \leq 364 \cdot 2^{-2n} .$$

The expression (25) allows us to show that the distribution p_{Ψ_a} obtained by measuring the P-quadrature of the state Ψ_a has the property required by Proposition 1. Indeed, the monotonicity of the L^1-norm distance under measurement implies that the distributions p_ρ and p_σ obtained by measuring any two states $\rho, \sigma \in \mathcal{B}(L^2(\mathbb{R}))$ satisfy

$$\|p_\rho - p_\sigma\|_1 \leq \|\rho - \sigma\|_1 . \quad (26)$$

Furthermore, we have the following statement about the distribution p_{Φ_a} obtained from a P-quadrature measurement of mode A of the state Φ_a.

Proposition 3 (Distribution of measurement outcomes)**.** *Let $N \geq 2$. For $a \in \mathbb{Z}_N^*$ let $\Phi_a \in L^2(\mathbb{R})^{\otimes 3} \otimes \mathbb{C}^2$ be the state defined by Eq. (25) of Proposition 2, with the parameters $(m, R, \kappa_{\mathrm{A}}, \Delta_{\mathrm{A}}, \kappa_{\mathrm{B}}, \Delta_{\mathrm{B}}, \Delta_{\mathrm{C}})$ specified by Supplementary Table 1 and with $\varepsilon_{\mathrm{B}} = \sqrt{\Delta_{\mathrm{B}}}$ and $\varepsilon_{\mathrm{C}} = \sqrt{\Delta_{\mathrm{C}}}$. Let $p_{\Phi_a} : \mathbb{R} \to [0, \infty)$ be the probability density function of the distribution of outcomes when performing a P-quadrature measurement on mode A of Φ_a. Then the family of distributions $\{p_{\Phi_a}\}_{a \in \mathbb{Z}_N^*}$ is suitable.*

With these ingredients, we are ready to prove Lemma 1 (of the main text). Indeed, Proposition 2 combined with Eq. (26) and Proposition 3 imply that the family of output distributions $\{p_{\Psi_a}\}_{a\in\mathbb{Z}_N^*}$ of the circuit $\mathcal{Q}_{a,N}$ is suitable. That is, for $a \in \mathbb{Z}_N^*$ we have (for sufficiently large N) that

$$\begin{aligned}\min_{d\in\mathbb{Z}_r} \int_{\Gamma_d(a)} p_{\Psi_a}(w)dw &\geq \left(\min_{d\in\mathbb{Z}_r} \int_{\Gamma_d(a)} p_{\Phi_a}(w)dw\right) - \|p_{\Psi_a} - p_{\Phi_a}\|_1 \\ &\geq \left(\min_{d\in\mathbb{Z}_r} \int_{\Gamma_d(a)} p_{\Phi_a}(w)dw\right) - \|\Psi - \Phi\|_1 \\ &\geq \Omega\left(1\right) \cdot \frac{1}{r(a)} - 364 \cdot 2^{-2n} \\ &\geq \Omega\left(1\right) \cdot \frac{1}{r(a)} \ , \end{aligned} \tag{27}$$

where we used the triangle inequality to obtain the first inequality and $r(a) < N < 2^n$, in the last step. We note that $\Omega(1)$ is independent of a. This proves Lemma 1 (if the main text).

It remains to prove Theorem 1. To do so we use the GKP state preparation protocol of [2] which we denote by $\mathcal{P}^{\mathsf{GKP}}_{\kappa,\Delta}$ in the following. This protocol prepares a mixed state close (in trace distance) to the GKP state $|\mathsf{GKP}_{\kappa,\Delta}\rangle$, see Theorem 2.2 below.

Theorem 2.2 ([2, Theorem 5.1] (see also Corollary 5.3))**.** *There exists a protocol $\mathcal{P}^{\mathsf{GKP}}_{\kappa,\Delta}$ that outputs a classical "success" flag with probability at least $1/10$, and has the property that the output state $\rho \in \mathcal{B}(L^2(\mathbb{R}))$ conditioned on success satisfies*

$$\|\rho - \mathsf{GKP}_{\kappa,\Delta}\|_1 \leq O(\Delta^{1/2}) + O(\kappa^{1/3}) \ ,$$

in the limit $(\kappa,\Delta) \to (0,0)$. The protocol uses $O(\log 1/\kappa)+O(\log 1/\Delta)$ operations of the form (i)–(iv) *of the main text, where some of the involved squeezing- and displacement operations are classically controlled by (efficiently computable functions of) the measurement result associated with a Q-quadrature measurement.*

Due to their central importance, various protocols for preparation of approximate GKP states exist [4–7]. As the primary focus in this context is on detecting small (i.e., constant) shifts, existing protocols typically target constant parameters (κ,Δ). Furthermore, in the error correction context, it often suffices to measure the quality of prepared states in terms of effective squeezing parameters which capture the degree of invariance with respect to small shifts [5, 7]. To our knowledge, the use of approximate GKP states in a quantum algorithmic context is new. This application requires significantly more stringent (i.e., non-constant) parameter choices for (κ,Δ), as well as a fidelity guarantee. Theorem 2.2 provides this.

Let Ψ_a be the final state before the measurement of the circuit $\mathcal{Q}_{a,N}$. Let ρ_a be the final (mixed) state before the measurement in the circuit $\mathcal{W}_{a,N}(e^{-iRP}\sigma_{\kappa_{\mathrm{A}},\Delta_{\mathrm{A}}}e^{iRP}, \sigma_{\kappa_{\mathrm{B}},\Delta_{\mathrm{B}}}, \Psi_{\Delta_{\mathrm{C}}})$, where we used the approximate GKP state preparation protocol $\mathcal{P}^{\mathsf{GKP}}_{\kappa_i,\Delta_i}$ to generate the state $\sigma_{\kappa_i,\Delta_i}$ in modes $i = \mathrm{A},\mathrm{B}$. Using the triangle inequality together with (26) yields

$$\min_{d\in\mathbb{Z}_r} \int_{\Gamma_d(a)} p_{\rho_a}(w)dw \geq \left(\min_{d\in\mathbb{Z}_r} \int_{\Gamma_d(a)} p_{\Psi_a}(w)dw\right) - \|\rho_a - \Psi_a\|_1 \ .$$

By the invariance of the L^1-norm distance under unitaries we obtain

$$
\begin{aligned}
\|\rho_a - \Psi_a\|_1 &= \|\sigma_{\kappa_A,\Delta_A} \otimes \sigma_{\kappa_B,\Delta_B} \otimes \Psi_{\Delta_C} \otimes |0\rangle\langle 0| - \mathsf{GKP}_{\kappa_A,\Delta_A} \otimes \mathsf{GKP}_{\kappa_B,\Delta_B} \otimes \Psi_{\Delta_C} \otimes |0\rangle\langle 0|\|_1 \\
&\leq \|\sigma_{\kappa_A,\Delta_A} - \mathsf{GKP}_{\kappa_A,\Delta_A}\|_1 + \|\sigma_{\kappa_B,\Delta_B} - \mathsf{GKP}_{\kappa_B,\Delta_B}\|_1 \qquad (28) \\
&\leq O(\Delta_A^{1/2}) + O(\kappa_A^{1/3}) + O(\Delta_B^{1/2}) + O(\kappa_B^{1/3}) \qquad (29) \\
&= O(2^{-8n} + 2^{-16n/3} + 2^{-9n^2/2} + 2^{-18n^2/3}) \\
&\leq O(2^{-4n})
\end{aligned}
$$

where we used that $\|\rho_1 \otimes \sigma - \rho_2 \otimes \sigma\|_1 = \|\rho_1 - \rho_2\|_1$ for states ρ_1, ρ_2, σ, and that

$$
\begin{aligned}
\|\rho_A \otimes \rho_B - \sigma_A \otimes \sigma_B\|_1 &\leq \|\rho_A \otimes \rho_B - \sigma_A \otimes \rho_B\|_1 + \|\sigma_A \otimes \rho_B - \sigma_A \otimes \sigma_B\|_1 \\
&\leq \|\rho_A - \sigma_A\|_1 + \|\rho_B - \sigma_B\|_1
\end{aligned}
$$

for states $\rho_A, \rho_B, \sigma_A, \sigma_B$ to obtain Eq. (28) and Theorem 2.2 (with the parameters $\kappa_A, \Delta_A, \kappa_B, \Delta_B, \Delta_C$ specified in Supplementary Table 1) to obtain Eq. (29). Combining this with (27) and using the fact that $r(a) < 2^n$ we obtain

$$
\min_{d\in\mathbb{Z}_r} \int_{\Gamma_d(a)} p_{\rho_a}(w)dw \geq \Omega(1) \cdot \frac{1}{r(a)} - O(2^{-4n}) \geq \Omega(1) \cdot \frac{1}{r(a)} .
$$

Thus Proposition 1 implies that there exists a classical polynomial-time algorithm which when applied to a single sample from the outcome distribution yields a factor of N with probability at least $\Omega(1/\log n)$. A simple amplification with $\log n$ repetitions yields the claim (II) of Theorem 1. Claim (I) of Theorem 1 is obtained by realizing the circuit $\mathcal{Q}_{a,N}$ (see Figure 3 of the main text) together with the preparation of the initial states with the parameters $(m, R, \kappa_A, \Delta_A, \kappa_B, \Delta_B, \Delta_C)$ given in Supplementary Table 1. We analyzed the size and depth of the circuit $\mathcal{W}_{a,N}(e^{-iRP}Ш_1^{(R)}, \mathsf{GKP}, |0\rangle)$ in Section 1.5 which did not accounted for the initial state preparation but for the circuit $\mathcal{W}_{a,N}$ only. To prepare GKP states we use the protocol $\mathcal{P}^{\mathsf{GKP}}_{\kappa,\Delta}$ of [2] producing states $\sigma_{\kappa,\Delta}$ close to the state $\mathsf{GKP}_{\kappa,\Delta}$, see Theorem 2.2. Thus the number of operations needed to prepare the initial state $e^{-iRP}\sigma_{\kappa_A,\Delta_A}$ on the mode A is $O(\log 1/\kappa_A) + O(\log 1/\Delta_A) + O(\log R) = O(n)$, where we haven also taken into account the translation by R performed by the decomposed unitary e^{-iRP} (cf. Section 1.1.2) and used the parameters $\kappa_A = \Delta_A = 2^{-16n}$. The initial state on the mode B is a GKP state prepared by protocol $\mathcal{P}^{\mathsf{GKP}}_{\kappa,\Delta}$ with parameters $\kappa_B = \Delta_B = 2^{-18n^2}$ (cf.Supplementary Table 1) which requires $O(n^2)$ elementary operations to prepare. The initial state on mode C is the squeezed vacuum state $|\Psi_{\Delta_C}\rangle = M_{\Delta_C}|\text{vac}\rangle$ whose preparation requires $O(|\log \Delta_C|) = O(n)$ elementary operations since $\Delta_C = 2^{-50n}$. Therefore, the number of elementary operations needed to implement $\mathcal{W}_{a,N}(e^{-iRP}\sigma_{\kappa_A,\Delta_A}e^{iRP}, \sigma_{\kappa_B,\Delta_B}, \Psi_{\Delta_C})$ including its initial states scales as $O(n^2)$ and so does its circuit size and depth.

3 Proof of Proposition 1: Classical post-processing

In this section, we prove Proposition 1, which we restate here for the reader's convenience.

Proposition 1 (Classical post-processing subroutine)**.** *There is a polynomial-time classical algorithm $\mathcal{A}$ such that the following holds. Given an n-bit integer $N \in \mathbb{N}$, assume that a pair (x, a) is generated as follows:*

(i) the element $a \sim U_{\mathbb{Z}_N^*}$ is drawn from the uniform distribution $U_{\mathbb{Z}_N^*}$ on $\mathbb{Z}_N^*$, and

(ii) $x \sim p_a$ is drawn from p_a, where $\{p_a \mid p_a : \mathbb{R} \to [0, \infty)\}_{a \in \mathbb{Z}_N^*}$ is a suitable family of probability density functions (see Definition 2.1).

Then the algorithm $\mathcal{A}$ — on input (x, a, N) — outputs a factor of N with probability at least

$$\Pr_{\substack{a \sim U_{\mathbb{Z}_N^*} \\ x \sim p_a}} [\, \mathcal{A}(x, a, N) \text{ divides } N \,] \geq \Omega\left(\frac{1}{\log n}\right) .$$

The runtime of $\mathcal{A}$ is polynomial in n.

Here we prove Proposition 1 by relating it to the classical post-processing subroutine of Shor's algorithm [8]. To this end, let us define the set of good inputs for the post-processing subroutine of Shor's algorithm.

Definition 3.1. *Let $N \in \mathbb{N}$. Let q be the smallest power of 2 such that $q > N^2$. We define the following set for all $a \in \mathbb{Z}_N^*$*

$$\mathsf{Good}_a := \left\{ c \in \mathbb{Z}_q \mid \exists d \in \mathbb{Z}_{r(a)} \text{ such that } \left| \frac{c}{q} - \frac{d}{r(a)} \right| \leq \frac{1}{2q} \right\} ,$$

where $r(a)$ is the order of a in $\mathbb{Z}_N^*$.

We use this set to paraphrase the success probability of the classical post-processing of Shor's algorithm in the following theorem.

Theorem 3.2 (Shor's post-processing subroutine, paraphrased from [8])**.** *There is an efficient classical algorithm $\mathcal{C}^{\mathsf{Shor}}$ with the following properties. Given an n-bit integer $N \in \mathbb{N}$, assume that a pair (c, a) is generated as follows:*

(i) the element $a \sim U_{\mathbb{Z}_N^*}$ is drawn from the uniform distribution $U_{\mathbb{Z}_N^*}$ on $\mathbb{Z}_N^*$, and

(ii) $c \sim P_a$ is drawn from P_a, where $\{P_a \mid P_a : \mathbb{Z}_q \to [0, 1]\}_{a \in \mathbb{Z}_N^*}$ is a family of probability distributions on $\mathbb{Z}_q$.

Then the algorithm $\mathcal{C}^{\mathsf{Shor}}$, given a sample $c \sim P_a$ and the two parameters a and N, outputs a factor of N with probability at least

$$\Pr_{\substack{a \sim U_{\mathbb{Z}_N^*} \\ c \sim P_a}} [\mathcal{C}^{\mathsf{Shor}}(c, a, N) \text{ divides } N] \geq \Omega(1) \cdot \min_{a \in \mathbb{Z}_N^*} \left(\frac{r(a)}{\log \log r(a)} \cdot \min_{c \in \mathsf{Good}_a} P_a(c) \right)$$

in time polynomial in n.

Theorem 3.2 shows that samples from the family $\{P_a\}_{a\in\mathbb{Z}_N^*}$ can be used to factor N as long as each P_a is such that every element $c \in \mathsf{Good}_a$ has non-negligible probability $P_a(c)$.

We can relax this condition somewhat, demanding only that $P_a(\{c-1, c, c+1\})$ is non-negligible for every $c \in \mathsf{Good}_a$. (Here $c-1, c+1 \in \mathbb{Z}_q$, i.e., substraction and addition is to be understood modulo q.) To this end, consider the following modified algorithm $\mathcal{C}^{\mathsf{Shor}'}$. It again takes $a \sim U_{\mathbb{Z}_N^*}$, a sample $c \sim P_a$ and N, and proceeds as follows: For $\ell \in \{-1, 0, 1\}$, the algorithm computes $\mathcal{C}^{\mathsf{Shor}}(c+\ell, a, N)$. It returns the corresponding number if one of the three outputs is a factor of N, and outputs failure otherwise. It is easy to check that (as a consequence of Theorem 3.2), we then also have

$$\Pr_{\substack{a\sim U_{\mathbb{Z}_N^*}\\ c\sim P_a}}[\mathcal{C}^{\mathsf{Shor}'}(c, a, N) \text{ divides } N] \geq \Omega(1)\cdot \min_{a\in\mathbb{Z}_N^*}\left(\frac{r(a)}{\log\log r(a)}\cdot \min_{c\in\mathsf{Good}_a} P_a\left(\{c-1, c, c+1\}\right)\right) \tag{30}$$

This reasoning can trivially be extended to probability density functions on the real line combined with a discretization procedure. Let $\mathsf{discret} : \mathbb{R} \to \mathbb{Z}_q$ be a "discretization" function. Then a probability distribution $\widetilde{p} : \mathbb{R} \to [0, 1]$ on $\mathbb{R}$ induces a distribution $p : \mathbb{Z}_q \to [0, 1]$ by

$$P(c) = \int_{\mathsf{discret}^{-1}(\{c\})} \tilde{p}(x)dx \qquad \text{for} \qquad c \in \mathbb{Z}_q\ .$$

In particular, statements such as (30) can be translated to families $\{p_a\}_{a\in\mathbb{Z}_N^*}$ of probability distributions on the real line. We argue that doing so with a specific discretization function $\mathsf{discret}$ leads to the concept of a suitable family of distributions, see Def. (2.1).

Concretely, consider the function

$$\begin{array}{rccc}\mathsf{discret}: & \mathbb{R} & \to & \mathbb{Z}_q \\ & x & \mapsto & \min\left(\underset{c\in\{0,\ldots,q\}}{\arg\min}\left|\frac{c}{q} - \mathrm{frac}(x)\right|\right) \mod q\end{array}$$

where $\mathrm{frac}(x) := x - \lfloor x\rfloor$ is the fractional part of $x \in \mathbb{R}$. It returns the closest multiple of $1/q$ to the fractional part of x. One can check that the function $\mathsf{discret}(\cdot)$ satisfies the following two properties:

(i) $\mathsf{discret}(x) = \mathsf{discret}(x+1) \quad$ for all $x \in \mathbb{R}$, i.e., it is translation-invariant, and

(ii) $\mathsf{discret}\left(\left\{\frac{c}{q}\right\} + \left(-\frac{1}{2q}, \frac{1}{2q}\right]\right) = \{c\} \quad$ for all $c \in \mathbb{Z}_q$, i.e., any argument $1/(2q)$-close to c/q for $c \in \mathbb{Z}_q$ gets mapped to c.

We use these properties to show the following relation between the unions of intervals $\Gamma_d(a)$ from Proposition 1 and the action of the discretization function.

Lemma 3.1. *Let $a \in \mathbb{Z}_N^*$, let $r(a)$ be the order of a in $\mathbb{Z}_N^*$, and let $c \in \mathsf{Good}_a$. Then there exists $d \in \mathbb{Z}_{r(a)}$ such that*

$$\mathsf{discret}^{-1}\left(\{c-1, c, c+1\}\right) \supset \Gamma_d(a)\ ,$$

where $\Gamma_d(a)$ is the union of intervals defined in Definition 2.1 (see Eq. (24)).

Proof. For brevity, let us write $r = r(a)$. We partition $\Gamma_d(a)$ as

$$\begin{aligned}\Gamma_d(a) &= \bigcup_{j\in\mathbb{Z}} \left[j + \frac{d}{r} - \frac{1}{2q}, j + \frac{d}{r} + \frac{1}{2q}\right] \\ &= \bigcup_{j\in\mathbb{Z}} \Gamma_{j,d} \qquad \text{where} \qquad \Gamma_{j,d} := \left[j + \frac{d}{r} - \frac{1}{2q}, j + \frac{d}{r} + \frac{1}{2q}\right] \ .\end{aligned}$$

We remark that the intervals $\Gamma_{j,d}$ are in both $j \in \mathbb{Z}$ and $d \in \mathbb{Z}_r$ pairwise disjoint (since $q > r \geq 1$). Furthermore, we have

$$\Gamma_{j,d} = \{j\} + \Gamma_{0,d} \qquad \text{for every} \qquad j \in \mathbb{Z} \tag{31}$$

where the addition is to be understood as a Minkowski sum. We claim that

$$\{c-1, c, c+1\} \supseteq \mathsf{discret}(\Gamma_{0,d}) \ . \tag{32}$$

With (31), this implies the claim because of property (i) of the discretization function $\mathsf{discret}$.

To prove (32), recall that the assumption $c \in \mathsf{Good}_a$ means that there is an element $d \in \mathbb{Z}_r$ such that

$$\left|\frac{c}{q} - \frac{d}{r}\right| \leq \frac{1}{2q} \ .$$

This implies that

$$\frac{c}{q} \in \Gamma_{0,d} \ .$$

Since $\Gamma_{0,d}$ is an interval of diameter $\frac{1}{q} < \frac{3}{2q}$, it follows that

$$\left(\frac{c}{q} - \frac{3}{2q}, \frac{c}{q} + \frac{3}{2q}\right] \supset \Gamma_{0,d} \ ,$$

because

$$\left(\frac{c}{q} - \frac{3}{2q}, \frac{c}{q} + \frac{3}{2q}\right] = \left(\left\{\frac{c-1}{q}\right\} + I_q\right) \cup \left(\left\{\frac{c}{q}\right\} + I_q\right) \cup \left(\left\{\frac{c+1}{q}\right\} + I_q\right) \ , \tag{33}$$

where $I_q = (-1/(2q), 1/(2q)]$. We illustrate the covering of $\Gamma_{0,d}$ by the three intervals in the RHS of Eq. (33) in Supplementary Figure 4. The claim (32) follows from property (ii) of the discretization function $\mathsf{discret}$. $\square$

In the following we prove Proposition 1. By the assumption that the family $\{p_a \mid p_a : \mathbb{R} \to [0, \infty)\}_{a\in\mathbb{Z}_N^*}$ of probability distributions is suitable (see Def. 2.1), we have

$$\min_{d\in\mathbb{Z}_r} \int_{\Gamma_d(a)} p_a(w)dw \geq \Omega(1) \cdot \frac{1}{r(a)} \qquad \text{for all} \qquad a \in \mathbb{Z}_N^* \ .$$

It follows from Lemma 3.1 that for every $a \in \mathbb{Z}_N^*$, the random variable $C_a = \mathsf{discret}(X_a)$, where $X_a \sim p_a$ is drawn from p_a, is distributed according to a distribution P_a that satisfies

$$P_a(\{c-1, c, c+1\}) \geq p_a(\Gamma_d(a)) \geq \Omega(1) \cdot \frac{1}{r(a)} \qquad \text{for every } c \in \mathsf{Good}_a \ .$$

(Here we write $p_a(A) = \int_A p_a(x)dx$ for $A \subseteq \mathbb{R}$.) Applying (30) to the family $\{P_a\}_{a\in\mathbb{Z}_N^*}$ of distributions yields

$$\Pr_{\substack{a\sim U_{\mathbb{Z}_N^*}\\ c\sim P_a}} [\mathcal{C}^{\mathsf{Shor}'}(c, a, N) \text{ divides } N] \geq \Omega(1) \cdot \left(\min_{a\in\mathbb{Z}_N^*} \frac{1}{\log\log r(a)}\right) \ .$$

This is the claim because $r(a) \leq N$.

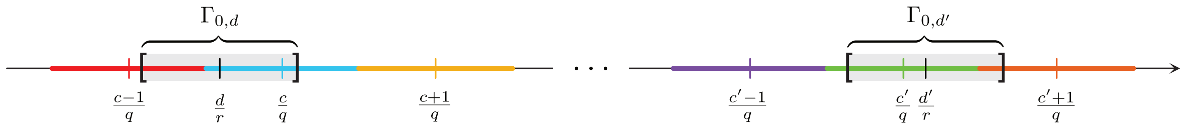

(a) A covering of the interval $\Gamma_{0,d}$ by three intervals $\{\frac{c-1}{q}\}+I_q$, $\{\frac{c}{q}\}+I_q$, $\{\frac{c+1}{q}\}+I_q$ colored in red, blue and yellow, respectively. The function $\mathsf{discret}$ maps these intervals to $c-1, c$ and $c+1$, respectively. In the given example, the set $\Gamma_{0,d}$ is covered by the two intervals $\{\frac{c-1}{q}\}+I_q$ and $\{\frac{c}{q}\}+I_q$ (red and blue). On the other hand, the displayed interval $\Gamma_{0,d'}$ is covered by the two intervals $\{\frac{c'}{q}\}+I_q$ and $\{\frac{c'+1}{q}\}+I_q$ (green and orange). In general, using three intervals $\{\frac{c-1}{q}\}+I_q$, $\{\frac{c}{q}\}+I_q$, $\{\frac{c+1}{q}\}+I_q$ is always sufficient to cover $\Gamma_{j,d}$ (in this illustration $c, c' \in \mathsf{Good}_a$).

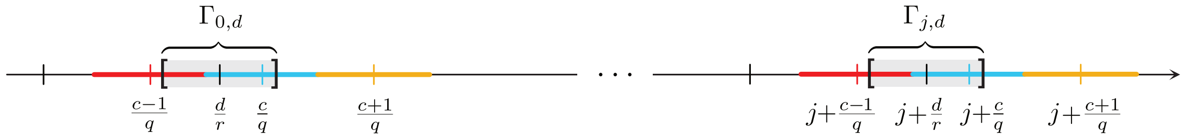

(b) A covering of interval $\Gamma_{j,d}$, for $j \in \mathbb{Z}$. These intervals are covered by $\{j+\frac{c-1}{q}\}+I_q$, $\{j+\frac{c}{q}\}+I_q$, $\{j+\frac{c+1}{q}\}+I_q$ and mapped by the function $\mathsf{discret}$ to $c-1, c, c+1$, respectively.

Supplementary Figure 4. An illustration of the covering of the interval $\Gamma_{0,d}$ by the three intervals $\{\frac{c-1}{q}\}+I_q$, $\{\frac{c}{q}\}+I_q$, $\{\frac{c+1}{q}\}+I_q$, where $I_q = (-1/(2q), 1/(2q)]$, used in the proof of Lemma 3.1 and explanation of the action of the discretization function $\mathsf{discret}$.

4 Proof of Proposition 2: Output state of circuit

This section is devoted to the proof of Proposition 2, which characterizes the output state Ψ_a of the circuit $\mathcal{Q}_{a,N} = \mathcal{W}_{a,N}(e^{-iRP}\mathsf{GKP}_{\kappa_\mathrm{A},\Delta_\mathrm{A}}, \mathsf{GKP}_{\kappa_\mathrm{B},\Delta_\mathrm{B}}, \Psi_{\Delta_\mathrm{C}})$ before the measurement (see Figure 3 of the main text). By definition, this output state Ψ_a is equal to

$$|\Psi_a\rangle = U_{a,N,m}\Big(e^{-iRP_\mathrm{A}}\,|\mathsf{GKP}_{\kappa_\mathrm{A},\Delta_\mathrm{A}}\rangle \otimes M_N\,|\mathsf{GKP}_{\kappa_\mathrm{B},\Delta_\mathrm{B}}\rangle \otimes |\Psi_{\Delta_\mathrm{C}}\rangle \otimes |0\rangle\Big)\ , \tag{34}$$

where $U_{a,N,m}$ is the unitary specified in Section 1.4 and the parameters $\kappa_\mathrm{A}, \Delta_\mathrm{A}, \kappa_\mathrm{B}, \Delta_\mathrm{B}, \Delta_\mathrm{C}$ and R, m are as in Supplementary Table 1. Proposition 2 (which we restate here for convenience) is the following.

Proposition 2 (Output state of the circuit $\mathcal{Q}_{a,N}$)**.** *Let $N \geq 8$ (i.e., $n \geq 4$) and $a \in \mathbb{Z}_N^*$. Let $(m, R, \kappa_\mathrm{A}, \Delta_\mathrm{A}, \kappa_\mathrm{B}, \Delta_\mathrm{B}, \Delta_\mathrm{C})$ be as specified in Supplementary Table 1. Let Ψ_a be the final state before the measurement of the circuit $\mathcal{Q}_{a,N}$ in Figure 3 of the main text. Let Φ_a be the state*

$$|\Phi_a\rangle = c_{\Phi_a}\sum_{z\in\mathbb{Z}} \eta_{\kappa_\mathrm{A}}(z-R)\,|\chi_{\Delta_\mathrm{A}}(z)\rangle \otimes e^{-i(a^z \mod N)P_\mathrm{B}} M_N \left|\mathsf{GKP}^{\varepsilon_\mathrm{B}}_{\kappa_\mathrm{B},\Delta_\mathrm{B}}\right\rangle \otimes \left|\Psi^{\varepsilon_\mathrm{C}}_{\Delta_\mathrm{C}}\right\rangle \otimes |0\rangle\ , \tag{25}$$

where c_{Φ_a} is a normalization constant and where $\varepsilon_\mathrm{B} = \sqrt{\Delta_\mathrm{B}}$ and $\varepsilon_\mathrm{C} = \sqrt{\Delta_\mathrm{C}}$. Then

$$\|\Psi_a - \Phi_a\|_1 \leq 364 \cdot 2^{-2n}\ .$$

Proof. To establish Proposition 2, we go through a sequence

$$\Psi_a = \Psi^{(0)}, \Psi^{(1)}, \ldots, \Psi^{(5)} = \Phi_a$$

of states and compare these pairwise. Their definition is given in Supplementary Table 2. We establish error bounds of the form

$$\left\|\Psi^{(j)} - \Psi^{(j-1)}\right\|_1 \leq \varepsilon^{(j)} \qquad \text{for} \qquad j \in \{1, \ldots, 5\}\ . \tag{35}$$

With the triangle inequality, this implies that

$$\|\Phi_a - \Psi_a\|_1 \leq \sum_{j=1}^{5} \varepsilon^{(j)} = 364 \cdot 2^{-2n}\ ,$$

implying Proposition 2.

Let us briefly sketch the main steps. The corresponding detailed proofs (including the estimates on the errors $\varepsilon^{(j)}$, $j \in \{1, \ldots, 5\}$) are deferred to subsequent sections.

Closeness of $\Psi^{(0)}$ and $\Psi^{(1)}$: The state $\Psi^{(0)} = \Psi_a$ of the circuit in Figure 3 of the main text before the measurement is given by Eq. (34), that is

$$\left|\Psi^{(0)}\right\rangle = U_{a,N,m}\left|\Phi^{(0)}\right\rangle \quad \text{where} \quad \left|\Phi^{(0)}\right\rangle = e^{-iRP_\mathrm{A}}\,|\mathsf{GKP}_{\kappa_\mathrm{A},\Delta_\mathrm{A}}\rangle \otimes M_N\,|\mathsf{GKP}_{\kappa_\mathrm{B},\Delta_\mathrm{B}}\rangle \otimes |\Psi_{\Delta_\mathrm{C}}\rangle \otimes |0\rangle\ .$$

For sufficiently small $\varepsilon_\mathrm{A}, \varepsilon_\mathrm{B}, \varepsilon_\mathrm{C}$, the result

$$\left|\widetilde{\Phi}^{(0)}\right\rangle = e^{-iRP_\mathrm{A}}\left|\mathsf{GKP}^{\varepsilon_\mathrm{A}}_{\kappa_\mathrm{A},\Delta_\mathrm{A}}\right\rangle \otimes M_N\left|\mathsf{GKP}^{\varepsilon_\mathrm{B}}_{\kappa_\mathrm{B},\Delta_\mathrm{B}}\right\rangle \otimes \left|\Psi^{\varepsilon_\mathrm{C}}_{\Delta_\mathrm{C}}\right\rangle \otimes |0\rangle \tag{36}$$

of restricting the support of $\Phi^{(0)}$ (and renormalizing) is close to $\Phi^{(0)}$. Indeed, this follows from the fact that the distance measure we consider (the L^1-distance) is invariant under unitaries and tensorizes, together with the fact that a truncated approximate GKP state $\mathsf{GKP}^{\varepsilon}_{\kappa,\Delta}$ is close to

$$
\begin{aligned}
\left|\Psi^{(0)}\right\rangle &= U_{a,N,m}(e^{-iRP_{\mathrm{A}}}\left|\mathsf{GKP}_{\kappa_{\mathrm{A}},\Delta_{\mathrm{A}}}\right\rangle && \otimes M_N \left|\mathsf{GKP}_{\kappa_{\mathrm{B}},\Delta_{\mathrm{B}}}\right\rangle \otimes \left|\Psi_{\Delta_{\mathrm{C}}}\right\rangle \otimes |0\rangle) \\
\left|\Psi^{(1)}\right\rangle &= c_1\, U_{a,N,m}(\Pi_{[-1/2,2R-1/2]}\, e^{-iRP_{\mathrm{A}}}\left|\mathsf{GKP}^{\varepsilon_{\mathrm{A}}}_{\kappa_{\mathrm{A}},\Delta_{\mathrm{A}}}\right\rangle && \otimes M_N \left|\mathsf{GKP}^{\varepsilon_{\mathrm{B}}}_{\kappa_{\mathrm{B}},\Delta_{\mathrm{B}}}\right\rangle \otimes \left|\Psi^{\varepsilon_{\mathrm{C}}}_{\Delta_{\mathrm{C}}}\right\rangle \otimes |0\rangle) \\
\left|\Psi^{(2)}\right\rangle &= c_2 \textstyle\sum_{z=0}^{2R-1} \eta_{\kappa_{\mathrm{A}}}(z-R)\left|\chi^{\varepsilon_{\mathrm{A}}}_{\Delta_{\mathrm{A}}}(z)\right\rangle && \otimes e^{-if_{a,N,m}(z)P_{\mathrm{B}}}\, M_N \left|\mathsf{GKP}^{\varepsilon_{\mathrm{B}}}_{\kappa_{\mathrm{B}},\Delta_{\mathrm{B}}}\right\rangle \otimes \left|\Psi^{\varepsilon_{\mathrm{C}}}_{\Delta_{\mathrm{C}}}\right\rangle \otimes |0\rangle \\
\left|\Psi^{(3)}\right\rangle &= c_3 \textstyle\sum_{z=0}^{2R-1} \eta_{\kappa_{\mathrm{A}}}(z-R)\left|\chi^{\varepsilon_{\mathrm{A}}}_{\Delta_{\mathrm{A}}}(z)\right\rangle && \otimes e^{-i(a^z \bmod N)P_{\mathrm{B}}} M_N \left|\mathsf{GKP}^{\varepsilon_{\mathrm{B}}}_{\kappa_{\mathrm{B}},\Delta_{\mathrm{B}}}\right\rangle \otimes \left|\Psi^{\varepsilon_{\mathrm{C}}}_{\Delta_{\mathrm{C}}}\right\rangle \otimes |0\rangle \\
\left|\Psi^{(4)}\right\rangle &= c_4 \textstyle\sum_{z\in\mathbb{Z}} \eta_{\kappa_{\mathrm{A}}}(z-R)\left|\chi^{\varepsilon_{\mathrm{A}}}_{\Delta_{\mathrm{A}}}(z)\right\rangle && \otimes e^{-i(a^z \bmod N)P_{\mathrm{B}}} M_N \left|\mathsf{GKP}^{\varepsilon_{\mathrm{B}}}_{\kappa_{\mathrm{B}},\Delta_{\mathrm{B}}}\right\rangle \otimes \left|\Psi^{\varepsilon_{\mathrm{C}}}_{\Delta_{\mathrm{C}}}\right\rangle \otimes |0\rangle \\
\left|\Psi^{(5)}\right\rangle &= c_5 \textstyle\sum_{z\in\mathbb{Z}} \eta_{\kappa_{\mathrm{A}}}(z-R)\left|\chi_{\Delta_{\mathrm{A}}}(z)\right\rangle && \otimes e^{-i(a^z \bmod N)P_{\mathrm{B}}} M_N \left|\mathsf{GKP}^{\varepsilon_{\mathrm{B}}}_{\kappa_{\mathrm{B}},\Delta_{\mathrm{B}}}\right\rangle \otimes \left|\Psi^{\varepsilon_{\mathrm{C}}}_{\Delta_{\mathrm{C}}}\right\rangle \otimes |0\rangle
\end{aligned}
$$

$\left\|\Psi^{(0)}-\Psi^{(1)}\right\|_1$	$\leq$	$\varepsilon^{(1)} = 9\cdot 2^{-2n}$	(Lemma 4.1)
$\left\|\Psi^{(1)}-\Psi^{(2)}\right\|_1$	$\leq$	$\varepsilon^{(2)} = 352\cdot 2^{-2n}$	(Lemma 5.11)
$\left\|\Psi^{(2)}-\Psi^{(3)}\right\|_1$	$\leq$	$\varepsilon^{(3)} = 2^{-2n}$	(Lemma 4.2)
$\left\|\Psi^{(3)}-\Psi^{(4)}\right\|_1$	$\leq$	$\varepsilon^{(4)} = 2^{-2n}$	(Lemma 4.3)
$\left\|\Psi^{(4)}-\Psi^{(5)}\right\|_1$	$\leq$	$\varepsilon^{(5)} = 2^{-2n}$	(Lemma 4.4)

Supplementary Table 2. Above, the states used in the analysis. The constants c_j are chosen such that the states are normalized. Below, the results establishing closeness $\varepsilon^{(j)}$ between the respective consecutive states $\Psi^{(j-1)}$ and $\Psi^{(j)}$ (cf. (35)).

an approximate GKP state $\mathsf{GKP}_{\kappa,\Delta}$, and a truncated squeezed vacuum state $\Psi^{\varepsilon}_{\Delta}$ is close to the squeezed vacuum state Ψ_{Δ} (for appropriate parameter choices).

Observe that $e^{-iRP}\left|\mathsf{GKP}^{\varepsilon_{\mathrm{A}}}_{\kappa_{\mathrm{A}},\Delta_{\mathrm{A}}}\right\rangle$ is centered around R (with an envelope whose variance is determined by κ_{A}), and has peaks (local maxima) supported around integers (with interval width $2\varepsilon_{\mathrm{A}}$). It follows that $e^{-iRP}\left|\mathsf{GKP}^{\varepsilon_{\mathrm{A}}}_{\kappa_{\mathrm{A}},\Delta_{\mathrm{A}}}\right\rangle$ has most of its support on the interval $[-1/2, 2R-1/2]$, i.e., the state $\widetilde{\Phi}^{(0)}$ (see Eq. (36)) is close to the state

$$
\left|\Phi^{(1)}\right\rangle = c_1\cdot \Pi_{[-1/2,2R-1/2]} e^{-iRP_{\mathrm{A}}}\left|\mathsf{GKP}^{\varepsilon_{\mathrm{A}}}_{\kappa_{\mathrm{A}},\Delta_{\mathrm{A}}}\right\rangle \otimes M_N\left|\mathsf{GKP}^{\varepsilon_{\mathrm{B}}}_{\kappa_{\mathrm{B}},\Delta_{\mathrm{B}}}\right\rangle \otimes \left|\Psi^{\varepsilon_{\mathrm{C}}}_{\Delta_{\mathrm{C}}}\right\rangle \otimes |0\rangle\ , \qquad (37)
$$

where $c_1 > 0$ is a normalization constant. Thus $\Phi^{(0)}$ and $\Phi^{(1)}$ are close (by the triangle inequality). Since $\left|\Psi^{(b)}\right\rangle = U_{a,N,m}\left|\Phi^{(b)}\right\rangle$ for $b\in\{0,1\}$, it follows that the state $\Psi^{(0)}$ is $\varepsilon^{(1)}$-close to the state

$$
\left|\Psi^{(1)}\right\rangle = c_1\cdot U_{a,N,m}\left(\Pi_{[-1/2,2R-1/2]} e^{-iRP_{\mathrm{A}}}\left|\mathsf{GKP}^{\varepsilon_{\mathrm{A}}}_{\kappa_{\mathrm{A}},\Delta_{\mathrm{A}}}\right\rangle \otimes M_N\left|\mathsf{GKP}^{\varepsilon_{\mathrm{B}}}_{\kappa_{\mathrm{B}},\Delta_{\mathrm{B}}}\right\rangle \otimes \left|\Psi^{\varepsilon_{\mathrm{C}}}_{\Delta_{\mathrm{C}}}\right\rangle \otimes |0\rangle\right)
$$

with an error $\varepsilon^{(1)}$ given in Lemma 4.1. We note that $\left|\Psi^{(1)}\right\rangle$ is simply the output state (i.e., the state before the measurement) of the circuit $\mathcal{W}_{a,N}(c_1\Pi_{[-1/2,2R-1/2]}e^{-iRP}\mathsf{GKP}^{\varepsilon_{\mathrm{A}}}_{\kappa_{\mathrm{A}},\Delta_{\mathrm{A}}}, \mathsf{GKP}^{\varepsilon_{\mathrm{B}}}_{\kappa_{\mathrm{B}},\Delta_{\mathrm{B}}}, \Psi^{\varepsilon_{\mathrm{C}}}_{\Delta_{\mathrm{C}}})$.
Closeness of $\Psi^{(1)}$ and $\Psi^{(2)}$: Arguing that $\Psi^{(1)}$ is close to $\Psi^{(2)}$ is the most technically challenging part of our proof. This is because it involves a detailed analysis of the action of the unitary $U_{a,N,m}$ when applied to states which are primarily supported around (certain) integers in position-space. To derive corresponding approximation results, we introduce a framework for approximate function evaluation by unitaries in Section 5. Here we only sketch the idea. As argued in Section 1.4, the unitary $U_{a,N,m}$ acts as

$$
U_{a,N,m}\left(|x\rangle\otimes|y\rangle\otimes|u\rangle\otimes|0\rangle\right) \ \propto\ \ |x\rangle\otimes|y+f_{a,N,m}(x)\rangle\otimes|u\rangle\otimes|0\rangle
$$

for all $x\in\{0,\ldots,2R-1\}$, $y,u\in\mathbb{R}$, i.e., it shifts the position y of the mode B by the pseudomodular power $f_{a,N,m}(x)$ of the position x of the mode A. We argue that — in a precise sense only

involving L^2-functions (i.e., normalizable states) — this is still approximately the case when the position $x \in \mathbb{R}$ of mode A belongs to the interval $[-1/2, 2R-1/2]$, and is additionally close to an integer. If these conditions are satisfied, the unitary $U_{a,N,m}$ has the effect of (approximately) shifting the position of the mode B by $f_{a,N,m}(\lfloor x \rceil)$, where $\lfloor x \rceil \in \mathbb{Z}$ denotes the integer closest to the position $x \in \mathbb{R}$ of the mode A, see Lemma 5.10 for a rigorous statement.

We can use this to show that $\Psi^{(1)}$ is close to $\Psi^{(2)}$ as follows: First recall that the state $\Psi^{(1)}$ is of the form $\Psi^{(1)} = U_{a,N,m}\Phi^{(1)}$ where $\Phi^{(1)}$ is the product state given in Eq. (37). In this product state, the state $c_1 \cdot \Pi_{[-1/2,2R-1/2]}e^{-iRP}\left|\mathsf{GKP}^{\varepsilon_\mathrm{A}}_{\kappa_\mathrm{A},\Delta_\mathrm{A}}\right\rangle$ on mode A is supported on $[-1/2, 2R-1/2]$ because of the projection $\Pi_{[-1/2,2R-1/2]}$, and additionally, every point x in the support is ε_A-close to an integer (because of the definition of the truncated approximate GKP state $\left|\mathsf{GKP}^{\varepsilon_\mathrm{A}}_{\kappa_\mathrm{A},\Delta_\mathrm{A}}\right\rangle$). Let $M \subseteq \mathbb{R}$, we define the set

$$M(\varepsilon) = M + [-\varepsilon, \varepsilon] = \{x + y \mid x \in M,\ |y| \leq \varepsilon\}\ .$$

In more detail, we have (using $e^{-iRP}|\chi^\varepsilon_\Delta(z)\rangle = |\chi^\varepsilon_\Delta(z+R)\rangle$ and the fact that considered parameter R is an integer)

$$\begin{aligned}\Pi_{[-1/2,2R-1/2]}e^{-iRP_\mathrm{A}}\left|\mathsf{GKP}^{\varepsilon_\mathrm{A}}_{\kappa_\mathrm{A},\Delta_\mathrm{A}}\right\rangle &\propto \sum_{z\in\mathbb{Z}} \eta_{\kappa_\mathrm{A}}(z)\Pi_{[-1/2,2R-1/2]}\left|\chi^{\varepsilon_\mathrm{A}}_{\Delta_\mathrm{A}}(z+R)\right\rangle \\ &= \sum_{z\in\mathbb{Z}} \eta_{\kappa_\mathrm{A}}(z-R)\Pi_{[-1/2,2R-1/2]}\left|\chi^{\varepsilon_\mathrm{A}}_{\Delta_\mathrm{A}}(z)\right\rangle \\ &= \sum_{z=0}^{2R-1} \eta_{\kappa_\mathrm{A}}(z-R)\left|\chi^{\varepsilon_\mathrm{A}}_{\Delta_\mathrm{A}}(z)\right\rangle\ ,\end{aligned} \tag{38}$$

showing that this function has support contained in $\{0, \ldots, 2R-1\}(\varepsilon_\mathrm{A})$. Inserting expression (38) into the definition of $\Phi^{(1)}$ gives

$$\left|\Phi^{(1)}\right\rangle \propto \sum_{z=0}^{2R-1}\sum_{y\in\mathbb{Z}} \eta_{\kappa_\mathrm{A}}(z-R)\eta_{\kappa_\mathrm{B}}(y)\left|\chi^{\varepsilon_\mathrm{A}}_{\Delta_\mathrm{A}}(z)\right\rangle \otimes M_N\left|\chi^{\varepsilon_\mathrm{B}}_{\Delta_\mathrm{B}}(y)\right\rangle \otimes \left|\Psi^{\varepsilon_\mathrm{C}}_{\Delta_\mathrm{C}}\right\rangle \otimes |0\rangle\ . \tag{39}$$

Now consider a state of the form

$$\left|\chi^{\varepsilon_\mathrm{A}}_{\Delta_{\mathrm{B}_A}}(z)\right\rangle \otimes M_N\left|\chi^{\varepsilon_\mathrm{B}}_{\Delta_\mathrm{B}}(y)\right\rangle \otimes \left|\Psi^{\varepsilon_C}_{\Delta_\mathrm{C}}\right\rangle \otimes |0\rangle \qquad \text{where} \qquad z \in \{0, \ldots, 2R-1\} \text{ and } y \in \mathbb{Z}\ .$$

By definition, any position $x \in \mathbb{R}$ occurring in the support of $\chi^{\varepsilon_\mathrm{A}}_{\Delta_\mathrm{A}}(z)$ is ε_A-close to the integer $z = \lfloor x \rceil$. It follows from the discussion of $U_{a,N,m}$ above that for $z \in \{0, \ldots, 2R-1\}$, we have

$$\begin{aligned}&U_{a,N,m}\left(\left|\chi^{\varepsilon_\mathrm{A}}_{\Delta_\mathrm{A}}(z)\right\rangle \otimes M_N\left|\chi^{\varepsilon_\mathrm{B}}_{\Delta_\mathrm{B}}(y)\right\rangle \otimes \left|\Psi^{\varepsilon_C}_{\Delta_\mathrm{C}}\right\rangle \otimes |0\rangle\right) \\ &\qquad \approx \left|\chi^{\varepsilon_\mathrm{A}}_{\Delta_\mathrm{A}}(z)\right\rangle \otimes e^{-if_{a,N,m}(z)P_\mathrm{B}}M_N\left|\chi^{\varepsilon_\mathrm{B}}_{\Delta_\mathrm{B}}(y)\right\rangle \otimes \left|\Psi^{\varepsilon_\mathrm{C}}_{\Delta_\mathrm{C}}\right\rangle \otimes \left|\Psi^{\varepsilon_\mathrm{C}}_{\Delta_\mathrm{C}}\right\rangle \otimes |0\rangle\ ,\end{aligned}$$

where we use the symbol $\approx$ to indicate that these states are close (see Lemma 5.10 for a rigorous statement). Applying this to each term in the superposition (39) and using the pairwise orthogonality of the states $\left\{\left|\chi^{\varepsilon_\mathrm{A}}_{\Delta_\mathrm{A}}(z)\right\rangle\right\}_{z\in\mathbb{Z}}$ we conclude that $\Psi^{(1)} = U_{a,N,m}\Phi^{(1)}$ is $\varepsilon^{(2)}$-close to the state

$$\left|\Psi^{(2)}\right\rangle = c_2\sum_{z=0}^{2R-1} \eta_{\kappa_\mathrm{A}}(z-R)\left|\chi^{\varepsilon_\mathrm{A}}_{\Delta_\mathrm{A}}(z)\right\rangle \otimes e^{-if_{a,N,m}(z)P_\mathrm{B}}M_N\left|\mathsf{GKP}^{\varepsilon_\mathrm{B}}_{\kappa_\mathrm{B},\Delta_\mathrm{B}}\right\rangle \otimes \left|\Psi^{\varepsilon_\mathrm{C}}_{\Delta_\mathrm{C}}\right\rangle \otimes |0\rangle\ ,$$

where an estimate of $\varepsilon^{(2)}$ is given in Lemma 5.11.

Closeness of $\Psi^{(2)}$ and $\Psi^{(3)}$: The state $M_N \left|\mathsf{GKP}^{\varepsilon_B}_{\kappa_B,\Delta_B}\right\rangle$ is approximately invariant under translations by (small) integer multiples of N. This implies that the state

$$e^{-if_{a,N,m}(z)P} M_N \left|\mathsf{GKP}^{\varepsilon_B}_{\kappa_B,\Delta_B}\right\rangle \approx\ e^{-i(f_{a,N,m}(z) \mod N)P_B} M_N \left|\mathsf{GKP}^{\varepsilon_B}_{\kappa_B,\Delta_B}\right\rangle \tag{40}$$

(see Lemma 7.5). Because the pseudomodular power satisfies

$$f_{a,N,m}(z) \equiv a^z \pmod N \qquad \text{for all} \qquad z \in \{0,\ldots,2R-1\}\ ,$$

Eq. (40) can be rewritten as

$$e^{-if_{a,N,m}(z)P_B} M_N \left|\mathsf{GKP}^{\varepsilon_B}_{\kappa_B,\Delta_B}\right\rangle \approx e^{-i(a^z \mod N)P_B} M_N \left|\mathsf{GKP}^{\varepsilon_B}_{\kappa_B,\Delta_B}\right\rangle \tag{41}$$

for all $z \in \{0,\ldots,2R-1\}$. With Eq. (41) and using the pairwise orthogonality of the states $\{\left|\chi^{\varepsilon_A}_{\Delta_A}(z)\right\rangle\}_{z\in\mathbb{Z}}$, we conclude that the state $\Psi^{(2)}$ is $\varepsilon^{(3)}$-close to the state $\Psi^{(3)}$ defined as

$$c_3 \sum_{z=0}^{2R-1} \eta_{\kappa_A}(z-R)\left|\chi^{\varepsilon_A}_{\Delta_A}(z)\right\rangle \otimes e^{-i(a^z \mod N)P_B} M_N \left|\mathsf{GKP}^{\varepsilon_B}_{\kappa_B,\Delta_B}\right\rangle \otimes |0\rangle\ ,$$

with an error $\varepsilon^{(3)}$ bounded by Lemma 4.2.

Closeness of $\Psi^{(3)}$ and $\Psi^{(4)}$: The state $\Psi^{(3)}$ is close to the state

$$\left|\Psi^{(4)}\right\rangle = c_4 \sum_{z\in\mathbb{Z}} \eta_{\kappa_A}(z-R)\left|\chi^{\varepsilon_A}_{\Delta_A}(z)\right\rangle \otimes e^{-i(a^z \mod N)P_B} M_N \left|\mathsf{GKP}^{\varepsilon_B}_{\kappa_B,\Delta_B}\right\rangle \otimes \left|\Psi^{\varepsilon_C}_{\Delta_C}\right\rangle \otimes |0\rangle$$

up to an error $\varepsilon^{(4)}$ estimated in Lemma 4.3. This follows from the fact that for the chosen parameter κ_A, the envelope $z \mapsto \eta_{\kappa_A}(z-R)$ is sufficiently small for $z \notin [0, 2R-1]$.

Closeness of $\Psi^{(4)}$ and $\Psi^{(5)}$: The state $\Psi^{(4)}$ is close to the state

$$\left|\Psi^{(5)}\right\rangle = c_5 \sum_{z\in\mathbb{Z}} \eta_{\kappa_A}(z-R)\left|\chi_{\Delta_A}(z)\right\rangle \otimes e^{-i(a^z \mod N)P_B} M_N \left|\mathsf{GKP}^{\varepsilon_B}_{\kappa_B,\Delta_B}\right\rangle \otimes \left|\Psi^{\varepsilon_C}_{\Delta_C}\right\rangle \otimes |0\rangle$$

up to an error $\varepsilon^{(5)}$ (see Lemma 4.4). Indeed, the states $\Psi^{(4)}$ and $\Psi^{(5)}$ only differ by the fact that the Gaussians in mode A are truncated in the state $\Psi^{(5)} = \Phi_a$. Furthermore, for $\varepsilon > 0$ sufficiently small compared to Δ, a centered Gaussian Ψ_Δ is close to its (normalized) truncation $\Psi^{\varepsilon}_{\Delta}$ to the interval $[-\varepsilon, \varepsilon]$, see Lemma 7.1.

This concludes the proof of Proposition 2, except for the task of establishing bounds $\varepsilon^{(1)}, \ldots, \varepsilon^{(5)}$ on the pairwise distances as in Eq. (35). □

4.1 Distance bounds from properties of approximate GKP states

In this section, we derive upper bounds on the distances

$$\|\Psi^{(0)} - \Psi^{(1)}\|_1,\ \|\Psi^{(2)} - \Psi^{(3)}\|_1,\ \|\Psi^{(3)} - \Psi^{(4)}\|_1 \ \text{ and } \ \|\Psi^{(4)} - \Psi^{(5)}\|_1\ .$$

These are simple consequences of basic properties of approximate GKP states (discussed in Section 2.1). The remaining bound on $\|\Psi^{(1)} - \Psi^{(2)}\|_1$ will be derived in Section 5.5.

The first bound of this kind relies on the fact that the state $|\mathsf{GKP}_{\kappa,\Delta}\rangle$ is close to its truncated version $\left|\mathsf{GKP}^{\varepsilon}_{\kappa,\Delta}\right\rangle$ for suitably chosen parameters (see Lemma 7.3), and the fact that most of the support of the state $\left|\mathsf{GKP}^{\varepsilon}_{\kappa,\Delta}\right\rangle$ is contained in the interval $[-r, r]$ for suitably large $r > 0$ (see Lemma 7.4). Together with the fact that the Gaussian state $|\Psi_\Delta\rangle$ is close to its truncated version $|\Psi^{\varepsilon}_{\Delta}\rangle$ for suitably chosen parameters (see Lemma 7.1).

Lemma 4.1. *Let $n \geq 4$. Let $(m, R, \kappa_\mathrm{A}, \Delta_\mathrm{A}, \kappa_\mathrm{B}, \Delta_\mathrm{B}, \Delta_\mathrm{C})$ be as specified in Supplementary Table 1, and let $\varepsilon_\mathrm{A} = \sqrt{\Delta_\mathrm{A}}$, $\varepsilon_\mathrm{B} = \sqrt{\Delta_\mathrm{B}}$ and $\varepsilon_\mathrm{C} = \sqrt{\Delta_\mathrm{C}}$. Consider the two states*

$$\begin{aligned}
\left|\Psi^{(0)}\right\rangle &= U_{a,N,m}\big(\qquad\qquad\qquad e^{-iRP_\mathrm{A}} \left|\mathsf{GKP}_{\kappa_\mathrm{A},\Delta_\mathrm{A}}\right\rangle \otimes M_N \left|\mathsf{GKP}_{\kappa_\mathrm{B},\Delta_\mathrm{B}}\right\rangle \otimes \left|\Psi_{\Delta_\mathrm{C}}\right\rangle \otimes |0\rangle \big) \\
\left|\Psi^{(1)}\right\rangle &= U_{a,N,m}\big(c_1 \cdot \Pi_{[-1/2,2R-1/2]} e^{-iRP_\mathrm{A}} \left|\mathsf{GKP}^{\varepsilon_\mathrm{A}}_{\kappa_\mathrm{A},\Delta_\mathrm{A}}\right\rangle \otimes M_N \left|\mathsf{GKP}^{\varepsilon_\mathrm{B}}_{\kappa_\mathrm{B},\Delta_\mathrm{B}}\right\rangle \otimes \left|\Psi^{\varepsilon_\mathrm{C}}_{\Delta_\mathrm{C}}\right\rangle \otimes |0\rangle \big) \ .
\end{aligned}$$

Then

$$\left\|\Psi^{(0)} - \Psi^{(1)}\right\|_1 \leq 9 \cdot 2^{-2n} =: \varepsilon^{(1)} \ .$$

Proof. We consider the two states

$$\begin{aligned}
\left|\Phi^{(0)}\right\rangle &= e^{-iRP_\mathrm{A}} \left|\mathsf{GKP}_{\kappa_\mathrm{A},\Delta_\mathrm{A}}\right\rangle \otimes M_N \left|\mathsf{GKP}_{\kappa_\mathrm{B},\Delta_\mathrm{B}}\right\rangle \otimes \left|\Psi_{\Delta_\mathrm{C}}\right\rangle \otimes |0\rangle \\
\left|\widetilde{\Phi}^{(0)}\right\rangle &= e^{-iRP_\mathrm{A}} \left|\mathsf{GKP}^{\varepsilon_\mathrm{A}}_{\kappa_\mathrm{A},\Delta_\mathrm{A}}\right\rangle \otimes M_N \left|\mathsf{GKP}^{\varepsilon_\mathrm{B}}_{\kappa_\mathrm{B},\Delta_\mathrm{B}}\right\rangle \otimes \left|\Psi^{\varepsilon_\mathrm{C}}_{\Delta_\mathrm{C}}\right\rangle \otimes |0\rangle \ .
\end{aligned}$$

Using the unitary of e^{-iRP} and M_N, we then have

$$\begin{aligned}
\left|\left\langle \Phi^{(0)}, \widetilde{\Phi}^{(0)}\right\rangle\right|^2 &= \left|\left\langle \mathsf{GKP}_{\kappa_\mathrm{A},\Delta_\mathrm{A}}, \mathsf{GKP}^{\varepsilon_\mathrm{A}}_{\kappa_\mathrm{A},\Delta_\mathrm{A}}\right\rangle\right|^2 \cdot \left|\left\langle \mathsf{GKP}_{\kappa_\mathrm{B},\Delta_\mathrm{B}}, \mathsf{GKP}^{\varepsilon_\mathrm{B}}_{\kappa_\mathrm{B},\Delta_\mathrm{B}}\right\rangle\right|^2 \cdot \left|\left\langle \Psi_{\Delta_\mathrm{C}}, \Psi^{\varepsilon_\mathrm{C}}_{\Delta_\mathrm{C}}\right\rangle\right|^2 \\
&\geq (1 - 9\Delta_\mathrm{A})(1 - 9\Delta_\mathrm{B})(1 - 2\Delta_\mathrm{C}) \\
&\geq 1 - 9(\Delta_\mathrm{A} + \Delta_\mathrm{B}) - 2\Delta_\mathrm{C} \ , \qquad (42)
\end{aligned}$$

where we used Lemma 7.3 (giving a lower bound on the overlap $|\langle \mathsf{GKP}_{\kappa,\Delta}, \mathsf{GKP}^{\varepsilon}_{\kappa,\Delta}|^2)$ twice and Lemma 7.1 (giving a lower bound on $|\langle \Psi_\Delta, \Psi^\varepsilon_\Delta\rangle|^2$) to obtain the first inequality. Combining (42) with the relation

$$\|\psi - \varphi\|_1 = 2\sqrt{1 - |\langle \psi, \varphi\rangle|^2} \qquad \text{for any two pure states } \psi \text{ and } \varphi \ , \qquad (43)$$

we obtain

$$\begin{aligned}
\left\|\Phi^{(0)} - \widetilde{\Phi}^{(0)}\right\|_1 &\leq 2\sqrt{9(\Delta_\mathrm{A} + \Delta_\mathrm{B}) + 2\Delta_\mathrm{C}} \\
&\leq 6\left(\sqrt{\Delta_\mathrm{A}} + \sqrt{\Delta_\mathrm{B}}\right) + 3\sqrt{\Delta_\mathrm{C}} \ , \qquad (44)
\end{aligned}$$

where we used $\sqrt{x+y} \leq \sqrt{x} + \sqrt{y}$ for all $x, y \geq 0$.

Now consider the state

$$\left|\Phi^{(1)}\right\rangle = c_1 \cdot \Pi_{[-1/2,2R-1/2]} e^{-iRP} \left|\mathsf{GKP}^{\varepsilon_\mathrm{A}}_{\kappa_\mathrm{A},\Delta_\mathrm{A}}\right\rangle \otimes M_N \left|\mathsf{GKP}^{\varepsilon_\mathrm{B}}_{\kappa_\mathrm{B},\Delta_\mathrm{B}}\right\rangle \otimes \left|\Psi^{\varepsilon_\mathrm{C}}_{\Delta_\mathrm{C}}\right\rangle \otimes |0\rangle \ .$$

Then

$$\left|\Phi^{(1)}\right\rangle = c_1 \cdot \Pi_{[-1/2,2R-1/2]} \left|\widetilde{\Phi}^{(0)}\right\rangle$$

by definition. It follows that

$$\begin{aligned}
\left|\left\langle \Phi^{(1)}, \widetilde{\Phi}^{(0)}\right\rangle\right|^2 &= c_1^2 \cdot \left|\langle \mathsf{GKP}^{\varepsilon_\mathrm{A}}_{\kappa_\mathrm{A},\Delta_\mathrm{A}}, e^{iRP} \Pi_{[-1/2,2R-1/2]} e^{-iRP} \left|\mathsf{GKP}^{\varepsilon_\mathrm{A}}_{\kappa_\mathrm{A},\Delta_\mathrm{A}}\right\rangle\right|^2 \\
&= c_1^2 \cdot \left|\langle \mathsf{GKP}^{\varepsilon_\mathrm{A}}_{\kappa_\mathrm{A},\Delta_\mathrm{A}}, \Pi_{[-R-1/2,R-1/2]} \left|\mathsf{GKP}^{\varepsilon_\mathrm{A}}_{\kappa_\mathrm{A},\Delta_\mathrm{A}}\right\rangle\right|^2 \\
&= \|\Pi_{[-R-1/2,R-1/2]} \mathsf{GKP}^{\varepsilon_\mathrm{A}}_{\kappa_\mathrm{A},\Delta_\mathrm{A}}\|^2 \ , \\
&\geq \|\Pi_{[-R/2,R/2]} \mathsf{GKP}^{\varepsilon_\mathrm{A}}_{\kappa_\mathrm{A},\Delta_\mathrm{A}}\|^2 \\
&\geq 1 - 2e^{-\frac{1}{4}\kappa_\mathrm{A}^2 R^2} \ ,
\end{aligned}$$

where we used that

$$e^{iRP}\Pi_{[-1/2,2R-1/2]}e^{-iRP} = \Pi_{[-R-1/2,R-1/2]} \ .$$

in the second step, the fact that

$$c_1^2 = \|\Pi_{[-1/2,2R-1/2]}e^{-iRP}\mathsf{GKP}^{\varepsilon_\mathrm{A}}_{\kappa_\mathrm{A},\Delta_\mathrm{A}}\|^{-2} = \|\Pi_{[-R-1/2,R-1/2]}\mathsf{GKP}^{\varepsilon_\mathrm{A}}_{\kappa_\mathrm{A},\Delta_\mathrm{A}}\|^{-2} \ .$$

in the third identity, the inclusion $[-R/2, R/2] \subseteq [-R-1/2, R-1/2]$ to obtain the first inequality, and Lemma 7.4 (giving a lower bound on $\|\Pi_{[}-r,r]\mathsf{GKP}^{\varepsilon}_{\kappa,\Delta}\|^2$) to obtain the second inequality. With the relation (43) we conclude that

$$\left\|\Phi^{(1)} - \widetilde{\Phi}^{(0)}\right\|_1 \leq 2\cdot\sqrt{2}e^{-\frac{1}{8}\kappa_\mathrm{A}^2R^2} \leq 3e^{-\frac{1}{8}\kappa_\mathrm{A}^2R^2} \ . \tag{45}$$

Combining (44) and (45) with the triangle inequality yields

$$\begin{aligned}\left\|\Psi^{(0)} - \Psi^{(1)}\right\|_1 &\leq 6\left(\sqrt{\Delta_\mathrm{A}} + \sqrt{\Delta_\mathrm{B}}\right) + 3\sqrt{\Delta_\mathrm{C}} + 3e^{-\frac{1}{8}\kappa_\mathrm{A}^2R^2} \\ &= 6\left(2^{-8n} + 2^{-9n^2}\right) + 3\cdot 2^{-25n} + 3e^{-\frac{1}{32}2^{2n}} \\ &\leq 6\cdot 2^{-2n} + 2^{-2n} + 2^{-2n} + 2^{-2n} \\ &\leq 9\cdot 2^{-2n}\end{aligned}$$

where we used the parameters $(\kappa_\mathrm{A}, \Delta_\mathrm{A}, \kappa_\mathrm{B}, \Delta_\mathrm{B}, \Delta_\mathrm{C}, R)$ from Supplementary Table 1 and the assumption that $n \geq 4$. The claim follows from this since we have $\Psi^{(b)} = U_{a,N,m}\Phi^{(b)}$ for $b = 0, 1$ by definition of these states and because the L^1-distance is invariant under unitaries. □

The following lemma is a consequence of the fact that the state $\left|\mathsf{GKP}^{\varepsilon}_{\kappa,\Delta}\right\rangle$ is approximately invariant under small integer shifts, see Lemma 7.5 for a detailed statement.

Lemma 4.2. *Assume $n \geq 3$. Let $(m, R, \kappa_\mathrm{A}, \Delta_\mathrm{A}, \kappa_\mathrm{B}, \Delta_\mathrm{B}, \Delta_\mathrm{C})$ be as specified in Supplementary Table 1. Let $\varepsilon_\mathrm{A} = \sqrt{\Delta_\mathrm{A}}$, $\varepsilon_\mathrm{B} = \sqrt{\Delta_\mathrm{B}}$ and $\varepsilon_\mathrm{C} = \sqrt{\Delta_\mathrm{C}}$. Consider the two states*

$$\begin{aligned}\left|\Psi^{(2)}\right\rangle &= c_2\sum_{z=0}^{2R-1}\eta_{\kappa_\mathrm{A}}(z-R)\left|\chi^{\varepsilon_\mathrm{A}}_{\Delta_\mathrm{A}}(z)\right\rangle \otimes e^{-if_{a,N,m}(z)P_\mathrm{B}}M_N\left|\mathsf{GKP}^{\varepsilon_\mathrm{B}}_{\kappa_\mathrm{B},\Delta_\mathrm{B}}\right\rangle \otimes \left|\Psi^{\varepsilon_\mathrm{C}}_{\Delta_\mathrm{C}}\right\rangle \otimes |0\rangle \ , \\ \left|\Psi^{(3)}\right\rangle &= c_3\sum_{z=0}^{2R-1}\eta_{\kappa_\mathrm{A}}(z-R)\left|\chi^{\varepsilon_\mathrm{A}}_{\Delta_\mathrm{A}}(z)\right\rangle \otimes e^{-i(a^z \mod N)P_\mathrm{B}}M_N\left|\mathsf{GKP}^{\varepsilon_\mathrm{B}}_{\kappa_\mathrm{B},\Delta_\mathrm{B}}\right\rangle \otimes \left|\Psi^{\varepsilon_\mathrm{C}}_{\Delta_\mathrm{C}}\right\rangle \otimes |0\rangle \ ,\end{aligned}$$

where $f_{a,N,m}(z)$ is the pseudomodular power (cf. Eq. (14)). Then

$$\left\|\Psi^{(2)} - \Psi^{(3)}\right\|_1 \leq 2^{-2n} =: \varepsilon^{(3)} \ .$$

Proof. By the pairwise orthogonality of the states $\{\chi^{\varepsilon_\mathrm{A}}_{\Delta_\mathrm{A}}(z)\}_{z\in\mathbb{Z}}$, we have

$$\begin{aligned}\left\langle\Psi^{(2)}, \Psi^{(3)}\right\rangle &= c_2c_3\sum_{z=0}^{2R-1}\eta_{\kappa_\mathrm{A}}(z-R)^2\left\langle M_N\mathsf{GKP}^{\varepsilon_\mathrm{B}}_{\kappa_\mathrm{B},\Delta_\mathrm{B}}, e^{i(f_{a,N,m}(z)-(a^z \mod N))P_\mathrm{B}}M_N\mathsf{GKP}^{\varepsilon_\mathrm{B}}_{\kappa_\mathrm{B},\Delta_\mathrm{B}}\right\rangle \\ &= c_2c_3\sum_{z=0}^{2R-1}\eta_{\kappa_\mathrm{A}}(z-R)^2\langle\mathsf{GKP}^{\varepsilon_\mathrm{B}}_{\kappa_\mathrm{B},\Delta_\mathrm{B}}, e^{id(z)P_\mathrm{B}}\mathsf{GKP}^{\varepsilon_\mathrm{B}}_{\kappa_\mathrm{B},\Delta_\mathrm{B}}\rangle\end{aligned} \tag{46}$$

where we used that $M_N^\dagger PM_N = P/N$ and introduced the abbreviation

$$d(z) = \frac{f_{a,N,m}(z) - (a^z \mod N)}{N} \ .$$

The following properties of $d(z)$ will be important for our analysis.

Claim 4.1. *Let $z \in \{0, \ldots, 2R-1\}$ be arbitrary. Then*

(i) $d(z) \in \mathbb{Z}$, i.e., $d(z)$ is an integer and

(ii) $|d(z)| \leq N^m$.

Proof. Statement (i) follows from the fact that the pseudomodular power satisfies the relation

$$f_{a,N,m}(z) \equiv a^z \mod N \qquad \text{for} \qquad z \in \{0, \ldots, 2^m - 1\} \ .$$

For our choice of parameters (m, R) (see Supplementary Table 1), we have the relation $2^m - 1 = 2R - 1$, implying that

$$f_{a,N,m}(z) \equiv a^z \mod N \qquad \text{for} \qquad z \in \{0, \ldots, 2R - 1\} \ .$$

This shows that $f_{a,N,m}(z) - a^z$ is an integer multiple of N proving (i).

To prove (i), recall that for any integer $z \in \mathbb{N}_0$, the pseudomodular power $f_{a,N,m}(z)$ (see Eq. (14)) is a product of m factors, each belonging to the set $\mathbb{Z}_N = \{0, \ldots, N-1\}$. Thus

$$f_{a,N,m}(z) \leq N^m \qquad \text{for every} \qquad z \in \mathbb{N}_0 \ .$$

In particular, we have

$$d(z) \leq \left| \frac{f_{a,N,m}(z)}{N} \right| + |a^z \mod N| \leq \frac{1}{N}(N^m + N) \leq N^m$$

as claimed. ∎

It follows from property (i) and Lemma 7.5 (giving a lower bound on $\langle \mathsf{GKP}^{\varepsilon}_{\kappa,\Delta}, e^{idP} \mathsf{GKP}^{\varepsilon}_{\kappa,\Delta} \rangle$ for integer d) that

$$\begin{aligned} \langle \mathsf{GKP}^{\varepsilon_{\mathrm{B}}}_{\kappa_{\mathrm{B}},\Delta_{\mathrm{B}}}, e^{id(z)P_{\mathrm{B}}} \mathsf{GKP}^{\varepsilon_{\mathrm{B}}}_{\kappa_{\mathrm{B}},\Delta_{\mathrm{B}}} \rangle &\geq 1 - \frac{\kappa_{\mathrm{B}}^2}{2} d(z)^2 \\ &\geq 1 - \frac{\kappa_{\mathrm{B}}^2}{2} N^{2m} \qquad \text{for all } z \in \{0, \ldots, 2R-1\} \ . \end{aligned} \tag{47}$$

where we used property (ii) to obtain the second inequality.

Applying inequality (47) to every summand in Eq. (46) gives

$$\left\langle \Psi^{(2)}, \Psi^{(3)} \right\rangle \geq c_2 c_3 \left(1 - \kappa_{\mathrm{B}}^2 N^{2m}/2\right) \cdot \sum_{z=0}^{2R-1} \eta_{\kappa_{\mathrm{A}}}(z - R)^2 \ .$$

Note that by the pairwise orthogonality of the states $\{\chi^{\varepsilon_{\mathrm{A}}}_{\Delta_{\mathrm{A}}}(z)\}_{z \in \mathbb{Z}}$ and the definition of the states $\Psi^{(2)}$ and $\Psi^{(3)}$, we have

$$c_2^{-2} = c_3^{-2} = \sum_{z=0}^{2R-1} \eta_{\kappa_{\mathrm{A}}}(z - R)^2 \ .$$

Thus

$$\left\langle \Psi^{(2)}, \Psi^{(3)} \right\rangle \geq 1 - \kappa_{\mathrm{B}}^2 N^{2m}/2 \ ,$$

The inequality $(1-x)^2 \geq 1-2x$ for all $x \in \mathbb{R}$ and the choice of (m, κ_{B}) from Supplementary Table 1 give

$$\begin{aligned}\left|\left\langle \Psi^{(2)}, \Psi^{(3)} \right\rangle\right|^2 &\geq 1 - \kappa_{\mathrm{B}}^2 N^{2m} \\ &\geq 1 - 2^{-2n^2} \ .\end{aligned}$$

Hence the relation between the overlap and the L^1-distance (cf. Eq. (43)) implies that

$$\begin{aligned}\left\|\Psi^{(2)} - \Psi^{(3)}\right\|_1 &\leq 2 \cdot 2^{-n^2} \\ &\leq 2^{-2n} \qquad \text{for } n \geq 3 \ .\end{aligned}$$

□

Another application of the fact that $\mathsf{GKP}^{\varepsilon}_{\kappa,\Delta} \in L^2(\mathbb{R})$ has most of its support in an interval of the form $[-r, r]$ (with suitably chosen r, see Lemma 7.4) is the following statement.

Lemma 4.3. *Let $n \geq 4$. Let $(m, R, \kappa_{\mathrm{A}}, \Delta_{\mathrm{A}}, \kappa_{\mathrm{B}}, \Delta_{\mathrm{B}}, \Delta_{\mathrm{C}})$ be as specified in Supplementary Table 1. Set $\varepsilon_{\mathrm{A}} = \sqrt{\Delta_{\mathrm{A}}}$, $\varepsilon_{\mathrm{B}} = \sqrt{\Delta_{\mathrm{B}}}$ and $\varepsilon_{\mathrm{C}} = \sqrt{\Delta_{\mathrm{C}}}$. Consider the two states*

$$\begin{aligned}\left|\Psi^{(3)}\right\rangle &= c_3 \textstyle\sum_{z=0}^{2R-1} \eta_{\kappa_{\mathrm{A}}}(z-R) \left|\chi^{\varepsilon_{\mathrm{A}}}_{\Delta_{\mathrm{A}}}(z)\right\rangle \otimes e^{-i(a^z \bmod N)P_{\mathrm{B}}} M_N \left|\mathsf{GKP}^{\varepsilon_{\mathrm{B}}}_{\kappa_{\mathrm{B}},\Delta_{\mathrm{B}}}\right\rangle \otimes \left|\Psi^{\varepsilon_{\mathrm{C}}}_{\Delta_{\mathrm{C}}}\right\rangle \otimes |0\rangle \\ \left|\Psi^{(4)}\right\rangle &= c_4 \textstyle\sum_{z\in\mathbb{Z}} \eta_{\kappa_{\mathrm{A}}}(z-R) \left|\chi^{\varepsilon_{\mathrm{A}}}_{\Delta_{\mathrm{A}}}(z)\right\rangle \otimes e^{-i(a^z \bmod N)P_{\mathrm{B}}} M_N \left|\mathsf{GKP}^{\varepsilon_{\mathrm{B}}}_{\kappa_{\mathrm{B}},\Delta_{\mathrm{B}}}\right\rangle \otimes \left|\Psi^{\varepsilon_{\mathrm{C}}}_{\Delta_{\mathrm{C}}}\right\rangle \otimes |0\rangle\end{aligned} \ .$$

Then

$$\left\|\Psi^{(3)} - \Psi^{(4)}\right\|_1 \leq 2^{-n} =: \varepsilon^{(4)} \ .$$

Proof. Due to the pairwise orthogonality of the states $\{\chi^{\varepsilon_{\mathrm{A}}}_{\Delta_{\mathrm{A}}}(z)\}_{z\in\mathbb{Z}}$ we have

$$\begin{aligned}c_3^{-2} &= \sum_{z=0}^{2R-1} \eta_{\kappa_{\mathrm{A}}}(z-R)^2 \\ c_4^{-2} &= \sum_{z\in\mathbb{Z}} \eta_{\kappa_{\mathrm{A}}}(z-R)^2 \ .\end{aligned}$$

It follows (again using orthogonality) that

$$\begin{aligned}|\langle \Psi^{(3)}, \Psi^{(4)} \rangle|^2 &= c_3^2 c_4^2 \left(\sum_{z=0}^{2R-1} \eta_{\kappa_{\mathrm{A}}}(z-R)^2 \right)^2 \\ &= \frac{\sum_{z=0}^{2R-1} \eta_{\kappa_{\mathrm{A}}}(z-R)^2}{\sum_{z\in\mathbb{Z}} \eta_{\kappa_{\mathrm{A}}}(z-R)^2} \\ &= \frac{\sum_{z=-R}^{R-1} \eta_{\kappa_{\mathrm{A}}}(z)^2}{\sum_{z\in\mathbb{Z}} \eta_{\kappa_{\mathrm{A}}}(z)^2} \ .\end{aligned} \tag{48}$$

Recalling the definition

$$\left|\mathsf{GKP}^{\varepsilon_{\mathrm{A}}}_{\kappa_{\mathrm{A}},\Delta_{\mathrm{A}}}\right\rangle = C_{\kappa_{\mathrm{A}},\Delta_{\mathrm{A}}} \sum_{z\in\mathbb{Z}} \eta_{\kappa_A}(z) \left|\chi^{\varepsilon_{\mathrm{A}}}_{\Delta_{\mathrm{A}}}(z)\right\rangle$$

and observing that $\chi^{\varepsilon_{\mathrm{A}}}_{\Delta_{\mathrm{A}}}(z)$ has support contained in $(z-1/2, z+1/2)$, we can write (48) as

$$\begin{aligned}|\langle \Psi^{(3)}, \Psi^{(4)} \rangle|^2 &= \left\|\Pi_{[-R-1/2,R-1/2]} \mathsf{GKP}^{\varepsilon_{\mathrm{A}}}_{\kappa_{\mathrm{A}},\Delta_{\mathrm{A}}}\right\|^2 \\ &\geq \|\Pi_{[-R/2,R/2]} \mathsf{GKP}^{\varepsilon_{\mathrm{A}}}_{\kappa_{\mathrm{A}},\Delta_{\mathrm{A}}}\|^2\end{aligned}$$

where we used that $[-R/2, R/2] \subseteq [-R-1/2, R-1/2]$. With Lemma 7.4 (bounding the norm of $\Pi_{[-r,r]}\mathsf{GKP}^{\varepsilon}_{\kappa,\Delta}$) it follows that

$$\left|\left\langle \Psi^{(3)}, \Psi^{(4)} \right\rangle\right|^2 \geq 1 - 2e^{-\frac{1}{4}\kappa_{\mathrm{A}}^2 R^2} \ .$$

Hence by the relation (43) between the overlap and the L^1-norm distance it follows that

$$\left\|\Psi^{(3)} - \Psi^{(4)}\right\|_1 \leq 2 \cdot \sqrt{2} e^{-\frac{1}{8}\kappa_{\mathrm{A}}^2 R^2} \leq 3e^{-\frac{1}{8}\kappa_{\mathrm{A}}^2 R^2} = 3e^{-\frac{1}{32}2^{2n}} \leq 2^{-2n}$$

for the choice of (κ_{A}, R) as in Supplementary Table 1 and $n \geq 4$, as claimed. □

The following result only relies on the fact that the normalization constants $C_{\kappa,\Delta}$ and C_κ in the definition of $|\mathsf{GKP}_{\kappa,\Delta}\rangle$ respectively $\left|\mathsf{GKP}^{\varepsilon}_{\kappa,\Delta}\right\rangle$ are close to each other (see Lemma 7.2).

Lemma 4.4. *Let $n \geq 1$. Let $(m, R, \kappa_{\mathrm{A}}, \Delta_{\mathrm{A}}, \kappa_{\mathrm{B}}, \Delta_{\mathrm{B}}, \Delta_{\mathrm{C}})$ be as specified in Supplementary Table 1. Set $\varepsilon_{\mathrm{A}} = \sqrt{\Delta_{\mathrm{A}}}$, $\varepsilon_{\mathrm{B}} = \sqrt{\Delta_{\mathrm{B}}}$, $\varepsilon_{\mathrm{C}} = \sqrt{\Delta_{\mathrm{C}}}$. Consider the two states*

$$\begin{aligned} \left|\Psi^{(4)}\right\rangle &= c_4 \textstyle\sum_{z\in\mathbb{Z}} \eta_{\kappa_{\mathrm{A}}}(z-R) \left|\chi^{\varepsilon_{\mathrm{A}}}_{\Delta_{\mathrm{A}}}(z)\right\rangle \otimes e^{-i(a^z \mod N)P_{\mathrm{B}}} M_N \left|\mathsf{GKP}^{\varepsilon_{\mathrm{B}}}_{\kappa_{\mathrm{B}},\Delta_{\mathrm{B}}}\right\rangle \otimes \left|\Psi^{\varepsilon_{\mathrm{C}}}_{\Delta_{\mathrm{C}}}\right\rangle \otimes |0\rangle \ , \\ \left|\Psi^{(5)}\right\rangle &= c_5 \textstyle\sum_{z\in\mathbb{Z}} \eta_{\kappa_{\mathrm{A}}}(z-R) \left|\chi_{\Delta_{\mathrm{A}}}(z)\right\rangle \otimes e^{-i(a^z \mod N)P_{\mathrm{B}}} M_N \left|\mathsf{GKP}^{\varepsilon_{\mathrm{B}}}_{\kappa_{\mathrm{B}},\Delta_{\mathrm{B}}}\right\rangle \otimes \left|\Psi^{\varepsilon_{\mathrm{C}}}_{\Delta_{\mathrm{C}}}\right\rangle \otimes |0\rangle \ . \end{aligned}$$

Then

$$\left\|\Psi^{(4)} - \Psi^{(5)}\right\|_1 \leq 2^{-2n} =: \varepsilon^{(5)} \ .$$

Proof. For brevity, let us introduce the states

$$|\theta(z)\rangle = e^{-i(a^z \mod N)P_{\mathrm{B}}} M_N \left|\mathsf{GKP}^{\varepsilon_{\mathrm{B}}}_{\kappa_{\mathrm{B}},\Delta_{\mathrm{B}}}\right\rangle \otimes \left|\Psi^{\varepsilon_{\mathrm{C}}}_{\Delta_{\mathrm{C}}}\right\rangle \qquad \text{for} \qquad z \in \mathbb{Z} \ .$$

It is easy to check that

$$0 \leq \langle\theta(z), \theta(z')\rangle \leq 1 \qquad \text{for all} \qquad z, z' \in \mathbb{Z} \ . \tag{49}$$

(In particular, these inner products are real-valued.) In terms of these states, we have

$$\begin{aligned} \left|\Psi^{(4)}\right\rangle &= c_4 \sum_{z\in\mathbb{Z}} \eta_{\kappa_{\mathrm{A}}}(z-R) \left|\chi^{\varepsilon_{\mathrm{A}}}_{\Delta_{\mathrm{A}}}(z)\right\rangle \otimes |\theta(z)\rangle \\ \left|\Psi^{(5)}\right\rangle &= c_5 \sum_{z\in\mathbb{Z}} \eta_{\kappa_{\mathrm{A}}}(z-R) \left|\chi_{\Delta_{\mathrm{A}}}(z)\right\rangle \otimes |\theta(z)\rangle \ . \end{aligned}$$

Thus

$$\begin{aligned} \langle\Psi^{(4)}, \Psi^{(5)}\rangle &= c_4 c_5 \sum_{z,z'\in\mathbb{Z}} \eta_{\kappa_{\mathrm{A}}}(z-R)\eta_{\kappa_{\mathrm{A}}}(z'-R)\langle\chi^{\varepsilon_{\mathrm{A}}}_{\Delta_{\mathrm{A}}}(z), \chi_{\Delta_{\mathrm{A}}}(z')\rangle \cdot \langle\theta(z), \theta(z')\rangle \\ &\geq c_4 c_5 \sum_{z\in\mathbb{Z}} \eta_{\kappa_{\mathrm{A}}}(z-R)^2 \langle\chi^{\varepsilon_{\mathrm{A}}}_{\Delta_{\mathrm{A}}}(z), \chi_{\Delta_{\mathrm{A}}}(z)\rangle \end{aligned} \tag{50}$$

where we used (49) and the fact that

$$\langle\chi^{\varepsilon_{\mathrm{A}}}_{\Delta_{\mathrm{A}}}(z), \chi^{\varepsilon_{\mathrm{A}}}_{\Delta_{\mathrm{A}}}(z')\rangle \geq 0 \qquad \text{for all} \qquad z, z' \in \mathbb{Z} \ . \tag{51}$$

By the pairwise orthogonality of the states $\left\{\left|\chi^{\varepsilon_{\mathrm{A}}}_{\Delta_{\mathrm{A}}}(z)\right\rangle\right\}_{z\in\mathbb{Z}}$, we have

$$c_4^{-2} = \sum_{z\in\mathbb{Z}} \eta_{\kappa_{\mathrm{A}}}(z-R)^2 = \sum_{z\in\mathbb{Z}} \eta_{\kappa_{\mathrm{A}}}(z)^2$$

and we conclude that

$$c_4 = C_{\kappa_A} \tag{52}$$

is equal to the normalization constant of the state $\mathsf{GKP}^{\varepsilon_A}_{\kappa_A,\Delta_A}$. On the other hand, we have

$$\begin{aligned}
c_5^{-2} &= \sum_{z,z'\in\mathbb{Z}} \eta_{\kappa_A}(z-R)\eta_{\kappa_A}(z'-R)\langle\chi_{\Delta_A}(z),\chi_{\Delta_A}(z')\rangle\cdot\langle\theta(z),\theta(z')\rangle \\
&\leq \sum_{z,z'\in\mathbb{Z}} \eta_{\kappa_A}(z-R)\eta_{\kappa_A}(z'-R)\langle\chi_{\Delta_A}(z),\chi_{\Delta_A}(z')\rangle \\
&= \sum_{z,z'\in\mathbb{Z}} \eta_{\kappa_A}(z)\eta_{\kappa_A}(z')\langle e^{iRP_A}\chi_{\Delta_A}(z), e^{iRP_A}\chi_{\Delta_A}(z')\rangle \\
&= \sum_{z,z'\in\mathbb{Z}} \eta_{\kappa_A}(z)\eta_{\kappa_A}(z')\langle\chi_{\Delta_A}(z),\chi_{\Delta_A}(z')\rangle = C^{-2}_{\kappa_A,\Delta_A} ,
\end{aligned}$$

where the inequality follows from (49) and (51), and where we used the fact that e^{iRP} is unitary. We conclude that c_5 is related to the normalization constant C_{κ_A,Δ_A} of the state $\mathsf{GKP}_{\kappa_A,\Delta_A}$ by

$$c_5 \geq C_{\kappa_A,\Delta_A} . \tag{53}$$

Combining Eqs. (52), (53) and (50) gives

$$\begin{aligned}
\langle\Psi^{(4)},\Psi^{(5)}\rangle &\geq C_{\kappa_A}C_{\kappa_A,\Delta_A}\sum_{z\in\mathbb{Z}}\eta_{\kappa_A}(z-R)^2\langle\chi^{\varepsilon_A}_{\Delta_A}(z),\chi_A(z)\rangle \\
&= C_{\kappa_A}C_{\kappa_A,\Delta_A}\sum_{z\in\mathbb{Z}}\eta_{\kappa_A}(z)^2\langle\chi^{\varepsilon_A}_{\Delta_A}(z+R),\chi_A(z+R)\rangle \\
&= C_{\kappa_A}C_{\kappa_A,\Delta_A}\sum_{z\in\mathbb{Z}}\eta_{\kappa_A}(z)^2\langle\chi^{\varepsilon_A}_{\Delta_A}(z),\chi_A(z)\rangle ,
\end{aligned}$$

where we used that $\left|\chi^{\varepsilon_A}_{\Delta_A}(z+R)\right\rangle = e^{iRP_A}|\chi^{\varepsilon_A}_{\Delta_A}(z)\rangle$ and similarly $|\chi_{\Delta_A}(z+R)\rangle = e^{iRP_A}|\chi_{\Delta_A}(z)\rangle$, as well as the fact that e^{iRP} is unitary. Using that $\langle\chi^{\varepsilon_A}_{\Delta_A}(z),\chi_A(z)\rangle = \langle\Psi^{\varepsilon_A}_{\Delta_A},\Psi_{\Delta_A}\rangle \geq \sqrt{1-2\Delta_A}$ again by the unitarity of translations, as well as Lemma 7.1, we conclude that

$$\begin{aligned}
\langle\Psi^{(4)},\Psi^{(5)}\rangle &\geq \sqrt{1-2\Delta_A}\cdot C_{\kappa_A}C_{\kappa_A,\Delta_A}\sum_{z\in\mathbb{Z}}\eta_{\kappa_A}(z)^2 \\
&= \sqrt{1-2\Delta_A}\cdot\frac{C_{\kappa_A,\Delta_A}}{C_{\kappa_A}}
\end{aligned}$$

by definition of C_{κ_A}. Since the choice of parameters (κ_A,Δ_A) (see Supplementary Table 1) ensures that $\kappa_A,\Delta_A\in(0,1/4)$ we can apply Lemma 7.2 to bound the fraction $\frac{C_{\kappa_A,\Delta_A}}{C_{\kappa_A}}$ of normalization constants, obtaining

$$\begin{aligned}
\left|\left\langle\Psi^{(4)},\Psi^{(5)}\right\rangle\right|^2 &\geq (1-2\Delta_A)\frac{(C_{\kappa_A,\Delta_A})^2}{(C_{\kappa_A})^2} \\
&\geq (1-2\Delta_A)(1-7\Delta_A) \\
&\geq 1-9\Delta_A ,
\end{aligned}$$

where we used the inequality $(1-x)(1-y)\geq 1-x-y$ for all $x,y>0$. The relation (43) relating the L^1-distance and the overlap therefore implies that

$$\left\|\Psi^{(4)}-\Psi^{(5)}\right\|_1 \leq 6\sqrt{\Delta_A} = 6\cdot 2^{-8n} \leq 2^{-2n} ,$$

where we used the choice of parameter Δ_A from Supplementary Table 1 and the assumption $n\geq 1$. This is the claim. □

It remains to establish an upper bound on the distance $\left\|\Psi^{(3)} - \Psi^{(4)}\right\|_1$. This requires an analysis of the unitary $U_{a,N,m}$, which we provide in the next section.

5 Approximate function evaluation for CV systems

The goal of this section is to analyze the action of the unitary $U_{a,N,m}$ (defined in Eq. (19)) on states that have support on non-integer positions. We will show that — in a sense made precise in this section — this unitary still (approximately) computes the pseudomodular power even if the positions of modes A and C are only close to integers. The corresponding statement is given in Lemma 5.10. Its formulation relies on a framework we introduce to formalize the notion of a unitary on a CV system approximately computing a function. In particular, we establish continuity bounds describing, e.g., what our LSB-gate does when applied to (position-encoded) reals that are only close to integers.

In the following, we are interested in unitaries acting on $L^2(\mathcal{X})$ where $\mathcal{X}$ is a measure space. In particular, we consider the case $\mathcal{X} = \mathbb{R}^m \times \{0,1\}$ (note that $L^2(\mathbb{R}^m) \cong L^2(\mathbb{R})^{\otimes m}$) and use that

$$L^2(\mathbb{R}^m \times \{0,1\}) \cong L^2(\mathbb{R}^m) \otimes \mathbb{C}^2 \cong L^2(\mathbb{R}^m, \mathbb{C}^2) \ .$$

More concretely, the isomorphism between the first two of these spaces is characterized by the following construction, using an orthonormal basis $\{|0\rangle, |1\rangle\}$ of $\mathbb{C}^2$. A function $\Psi \in L^2(\mathbb{R}^m \times \{0,1\})$ is mapped to $|\Psi_0\rangle \otimes |0\rangle + |\Psi_1\rangle \otimes |1\rangle$, equivalently referred to as a spinor (Ψ_0, Ψ_1), where $\Psi_i = \Psi(\cdot, i) \in L^2(\mathbb{R}^m)$ for $i \in \{0,1\}$. For two spinors (Ψ_0, Ψ_1), (Φ_0, Φ_1) the inner product on $L^2(\mathbb{R}^m) \otimes \mathbb{C}^2$ is then given by $\langle (\Psi_0, \Psi_1), (\Phi_0, \Phi_1) \rangle = \langle \Psi_0, \Phi_0 \rangle + \langle \Psi_1, \Phi_1 \rangle$, where $\langle \Psi_i, \Phi_i \rangle$ for $i \in \{0,1\}$ is the usual inner product on $L^2(\mathbb{R}^m)$. Finally, an element $\Psi \in L^2(\mathbb{R}^m, \mathbb{C}^2)$ corresponds to the spinor (Ψ_0, Ψ_1) where $\Psi_i(x) = \langle i, \Psi(x) \rangle$ is the coefficient of $|i\rangle$ in $|\Psi(x)\rangle \in \mathbb{C}^2$ for $i = 0, 1$ and $x \in \mathbb{R}^m$.

In the following, we use these isomorphic spaces interchangeably. Moreover, we will write an element Ψ in one of these spaces as

$$|\Psi\rangle = \int_{\mathbb{R}^m} dx \left(\Psi_0(x) |x\rangle \otimes |0\rangle + \Psi_1(x) |x\rangle \otimes |1\rangle \right) \ ,$$

where $|x\rangle$ is the (formal) tensor product of position-eigenstates $|x\rangle = |x_1\rangle \otimes \cdots \otimes |x_m\rangle$ for $x \in \mathbb{R}^m$. We define the support of a function $\Psi \in L^2(\mathbb{R}^m \times \{0,1\})$ as

$$\mathrm{supp}(\Psi) = \overline{\{(x,b) \in \mathbb{R}^m \times \{0,1\} \mid \Psi(x,b) \neq 0\}}$$

where $\overline{A}$ denotes the closure of a set A.

5.1 Exact function evaluation

In the following, we consider injective functions of the form

$$\begin{array}{rccc} f: & A \times B & \to & \mathbb{R}^m \times \{0,1\} \\ & (x,b) & \mapsto & f(x,b) = (f_1(x,b), f_2(x,b)) \ . \end{array}$$

where $A \subseteq \mathbb{R}^m$ is closed and $B \subseteq \{0,1\}$. Typically, we consider the case where $A = A_1 \times A_2 \times \cdots \times A_m$ is also a product set. Furthermore, we assume that for each $b \in B$, the function $f_1(\cdot, b) : A \to \mathbb{R}^m$ is a diffeomorphism. Such a function f defines an isometry

$$U_f : L^2(A \times B) \ \to \ U_f L^2(A \times B) \subseteq L^2(\mathbb{R}^m \times \{0,1\})$$

on the subspace $L^2(A \times B) \subseteq L^2(\mathbb{R}^m \times \{0,1\})$ of functions with support contained in $A \times B$. That is, the isometry U_f is defined by its action

$$U_f \left(|x\rangle \otimes |b\rangle \right) = J_{f_1(\cdot,b)}(x)^{1/2} \left(|f_1(x,b)\rangle \otimes |f_2(x,b)\rangle \right) \qquad \text{for } (x,b) \in A \times B \ , \tag{54}$$

where $J_{f_1(\cdot,b)}(x) = |\det D_{f_1(\cdot,b)}(x)|$ denotes the determinant of the Jacobi-matrix $D_{f_1(\cdot,b)}$ of $f_1(\cdot,b)$ at the point x. The factor $J_{f_1(\cdot,b)}(x)^{1/2}$ in Eq. (54) ensures that U_f maps the subspace $L^2(A\times B)$ isometrically to the image $U_f L^2(A\times B)$.

We note that the image of

$$|\Psi\rangle = \sum_{b\in B}\int_A dx\, \Psi(x,b)\, |x\rangle \otimes |b\rangle \qquad \text{for} \qquad \Psi \in L^2(A\times B)$$

is

$$U_f\,|\Psi\rangle = \sum_{c\in\{0,1\}}\int_{\mathbb{R}^m} dy\ (U_f\Psi)\,(y,c)\, |y\rangle \otimes |c\rangle$$

where we introduced the function $(U_f\Psi) \in L^2(\mathbb{R}^m \times \{0,1\})$ defined as

$$(U_f\Psi)\,(y,c) = \begin{cases} (J_{f_1}(\cdot,b))(x)^{-1/2}\Psi(x)\big|_{(x,b)=f^{-1}(y,c)} & \text{if } (y,c)\in f(A\times B) \\ 0 & \text{otherwise} \end{cases} .$$

Note that by construction we have $\mathrm{supp}(U_f\Psi) \subseteq \overline{f(A\times B)}$ whenever $\mathrm{supp}(\Psi) \subseteq A\times B$, i.e.,

$$U_f\Psi \in L^2(\overline{f(A\times B)}) \qquad \text{for every} \qquad \Psi \in L^2(A\times B)\ . \tag{55}$$

Note that the x-dependent prefactor $J_{f_1(\cdot,b)}(x)^{1/2}$ in Eq. (54) also implies that the composition is well-defined, i.e., for two injective functions $f : A\times B \to A'\times B' \subseteq \mathbb{R}^m \times \{0,1\}$, $g : A'\times B' \to \mathbb{R}^m\times\{0,1\}$ with $A\times B \subseteq \mathbb{R}^m\times\{0,1\}$ and diffeomorphic restrictions $f_1(\cdot,b)$, $g_1(\cdot,b')$ for $b\in B$ respectively $b'\in B'$, we have

$$U_g \cdot U_f = U_{g\circ f}\ :\ L^2(A\times B)\ \to\ U_{f\circ g}L^2(A\times B) \subseteq L^2(\mathbb{R}^m\times\{0,1\}) \tag{56}$$

Two special types of functions f are of particular interest for our analysis. First, suppose $f = g\times \mathsf{id}$ takes the form $f(x,b) = (g(x),b)$ with a diffeomorphism $g : A \to g(A)$. Then $U_{g\times\mathsf{id}}$ acts trivially on the qubit and

$$\begin{aligned} U_{g\times\mathsf{id}}\,|\Psi\rangle &= \sum_{b\in\{0,1\}}\int_{g(A)} dy\, J_g(g^{-1}(y))^{-1/2}\Psi(g^{-1}(y),b)\,|y\rangle\otimes|b\rangle \\ &= \sum_{b\in\{0,1\}}\int_{g(A)} dy\, J_{g^{-1}}(y)^{1/2}\Psi(g^{-1}(y),b)\,|y\rangle\otimes|b\rangle \end{aligned}$$

for $\Psi\in L^2(A\times\{0,1\})$.

Another case of interest is where $f = \mathsf{id}\times(h\oplus\mathsf{id})$ takes the form $f(x,b) = (x, h(x)\oplus b)$ for a function $h:\mathbb{R}^m\to\{0,1\}$. In this case we have

$$U_{\mathsf{id}\times(h\oplus\mathsf{id})}\,|\Psi\rangle = \sum_{b\in\{0,1\}}\int_{\mathbb{R}^m} dx\,\Psi(x,b)\,|x\rangle\otimes|h(x)\oplus b\rangle\ .$$

This case captures functions that extract information from a CV system into a qubit system.

5.2 Framework for approximate function evaluation

We will be interested in unitaries that approximately compute certain functions on states that are supported on the corresponding domain. To formalize this notion, we introduce the following definition.

Definition 5.1. *Let $A \times B \subseteq \mathbb{R}^m \times \{0,1\}$ be a closed set. Let $f : A \times B \to \mathbb{R}^m \times \{0,1\}$ be injective with diffeomorphic restrictions $f_1(\cdot, b)$ for $b \in B$. Let $\varepsilon \geq 0$. Let U be a unitary on $L^2(\mathbb{R}^m \times \{0,1\})$. We say that the unitary U ε-approximately computes f on $A \times B$ if*

$$\|U\Psi - U_f\Psi\|_1 \leq \varepsilon \qquad \text{for any state} \qquad |\Psi\rangle \in L^2(A \times B) \ . \tag{57}$$

We will call a real $\varepsilon > 0$ satisfying (57) an error bound for U.

In the following, we will use diagrammatic notation to specify the domain and range of a unitary U which ε-approximately computes a function $f : A \times B \to A' \times B'$ for $A, A' \subseteq \mathbb{R}^m$ and $B, B' \subseteq \{0,1\}$: We write

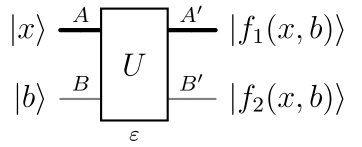

We often consider product sets $A = A_1 \times \cdots \times A_m$. If U ε-approximately computes a function $f : (A_1 \times \cdots \times A_m) \times B \to (A'_1 \times \cdots \times A'_m) \times B'$ in the sense of Definition 5.1, we specify the sets $A_1, \ldots, A_m \subseteq \mathbb{R}$, $B \in \{0,1\}$ corresponding to the domain of f, and the sets $A'_1, \ldots, A'_m \subseteq \mathbb{R}$, $B' \in \{0,1\}$ corresponding to the range of f by labeling the associated input- and output wires, as follows:

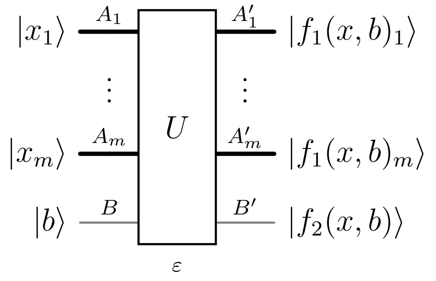

(58)

We will omit ε whenever U computes the function f exactly on $A \times B$.

The composition property (56) leads to the following lemma for the composition of two unitaries approximately computing two functions with compatible ranges and domains.

Lemma 5.1. *For $i \in \{1,2\}$, let $A_i \times B_i \subseteq \mathbb{R}^m \times \{0,1\}$ be a closed set, let $f^{(i)} : A_i \times B_i \to \mathbb{R}^m \times \{0,1\}$ and let $U^{(i)}$ be a unitary on $L^2(\mathbb{R}^m \times \{0,1\})$ which $\varepsilon^{(i)}$-approximately $f^{(i)}$ on $A_i \times B_i$. Assume further that*

$$f^{(1)}(A_1 \times B_1) \subseteq A_2 \times B_2 \ .$$

Then the unitary $U^{(2)}U^{(1)}$ $(\varepsilon^{(1)} + \varepsilon^{(2)})$-approximately computes the function $f^{(2)} \circ f^{(1)}$ on $A_1 \times B_1$.

Proof. Let us consider an arbitrary state Ψ with $\mathrm{supp}(\Psi) \subseteq A_1 \times B_1$. Using (55) we have $\mathrm{supp}(U_{f^{(1)}}\Psi) \subseteq \overline{f^{(1)}(A_1 \times B_1)} \subseteq A_2 \times B_2$ by the assumption that $A_2 \times B_2$ is closed. Using the assumption about $U^{(2)}$, we therefore have

$$\left\| U^{(2)}\left(U_{f^{(1)}}\Psi\right) - U_{f^{(2)}}\left(U_{f^{(1)}}\Psi\right) \right\|_1 \leq \varepsilon_2 \ . \tag{59}$$

By applying the triangle inequality and the invariance of the L^1-norm under unitaries, we obtain

$$\begin{aligned}\left\|U^{(2)}\left(U^{(1)}\Psi\right)-U_{f^{(2)}\circ f^{(1)}}\Psi\right\|_1 &\leq \left\|U^{(2)}\left(U^{(1)}\Psi\right)-U^{(2)}\left(U_{f^{(1)}}\Psi\right)\right\|_1+\left\|U^{(2)}\left(U_{f^{(1)}}\Psi\right)-U_{f^{(2)}\circ f^{(1)}}\Psi\right\|_1\\ &=\left\|U^{(1)}\Psi-U_{f^{(1)}}\Psi\right\|_1+\left\|U^{(2)}\left(U_{f^{(1)}}\Psi\right)-U_{f^{(2)}}\left(U_{f^{(1)}}\Psi\right)\right\|_1\\ &\leq \varepsilon_1+\varepsilon_2\ ,\end{aligned}$$

where we used that $U_{f^{(2)}}\left(U_{f^{(1)}}\Psi\right)=U_{f^{(2)}\circ f^{(1)}}\Psi$ (see Eq. (56)), and where the last inequality follows from the assumption on $U^{(1)}$ combined with (59). □

5.3 Approximate evaluation of functions $h:\mathbb{R}^m\to\{0,1\}$

In this section, we present a sufficient condition for a unitary U to ε-approximately compute a function $f:\mathbb{R}^m\times\{0,1\}\to\mathbb{R}^m\times\{0,1\}$ of the form $f=\mathsf{id}\times(h\oplus\mathsf{id})$ with $h:\mathbb{R}^m\to\{0,1\}$. Such a family of functions is able to extract information about the CV system into the qubit while keeping the CV system unchanged. An example is our LSB-extraction gate (cf. Section 1.1.4). We will use statements from this section to analyze its action on inputs close to integers (see Section 5.4.1).

To establish this sufficient condition, we need the following

Lemma 5.2. *Let $\mathcal{X}$ be a measure space. Let $0<\varepsilon\leq 1/2$ and $\phi\in L^2(\mathcal{X})$ with $\|\phi\|=1$ be arbitrary. For each $x\in\mathcal{X}$, let $\Psi(x),\Psi'(x)\in\mathcal{H}$ be normalized elements of a Hilbert space $\mathcal{H}$. Assume that*

$$|1-\langle\Psi'(x),\Psi(x)\rangle|\leq\varepsilon \qquad \textit{for all} \qquad x\in\mathcal{X}\ . \tag{60}$$

Consider the states

$$\begin{aligned}|\Phi\rangle&=\int_{\mathcal{X}}dx\ \phi(x)\,|x\rangle\otimes|\Psi(x)\rangle\\ |\Phi'\rangle&=\int_{\mathcal{X}}dx\ \phi(x)\,|x\rangle\otimes|\Psi'(x)\rangle\end{aligned}$$

in the Hilbert space $L^2(\mathcal{X},\mathcal{H})$. Then

$$|\langle\Phi,\Phi'\rangle|\geq 1-2\varepsilon\ .$$

Proof. Eq. (60) implies the bounds

$$\begin{aligned}\mathrm{Re}\langle\Psi'(x),\Psi(x)\rangle&\geq 1-\varepsilon \qquad (61)\\ \left|\mathrm{Im}\langle\Psi'(x),\Psi(x)\rangle\right|&\leq\varepsilon\end{aligned}$$

on the real- and imaginary parts of the overlap $\langle\Psi'(x),\Psi(x)\rangle$ for $x\in\mathcal{X}$. Hence we can bound

$$\begin{aligned}\left|\langle\Phi',\Phi\rangle\right| &= \left|\int dx|\phi(x)|^2\langle\Psi'(x),\Psi(x)\rangle\right|\\ &= \left|\int dx|\phi(x)|^2\left(\mathrm{Re}\langle\Psi'(x),\Psi(x)\rangle+i\mathrm{Im}\langle\Psi'(x),\Psi(x)\rangle\right)\right|\\ &\geq \left|\int dx|\phi(x)|^2\mathrm{Re}\langle\Psi'(x),\Psi(x)\rangle\right|-\left|\int dx|\phi(x)|^2\mathrm{Im}\langle\Psi'(x),\Psi(x)\rangle\right|\\ &\geq \left|\int dx|\phi(x)|^2\mathrm{Re}\langle\Psi'(x),\Psi(x)\rangle\right|-\int dx|\phi(x)|^2\left|\mathrm{Im}\langle\Psi'(x),\Psi(x)\rangle\right|\\ &\geq 1-2\varepsilon\ ,\end{aligned}$$

where the first inequality follows from the triangle inequality on $\mathbb{C}$ and the last inequality from (61). □

The following lemma gives the desired sufficient condition. For ease of later application, we give a "stable" version including an additional system $L^2(\mathbb{R}^\ell)$ where the function acts as the identity.

Lemma 5.3. *Let $0 < \varepsilon \leq 1/2$, let $A \subseteq \mathbb{R}^m$ be a closed set and $f : A \times \{0,1\} \to \mathbb{R}^m \times \{0,1\}$ be a function of the form $f = \mathsf{id} \times (h \oplus \mathsf{id})$, i.e., $f(x,b) = (x, h(x) \oplus b)$. Let $\{|\Omega(x,b)\rangle\}_{(x,b)\in A\times\{0,1\}} \subseteq \mathbb{C}^2$ be a family of qubit states such that*

$$|1 - \langle h(x) \oplus b, \Omega(x,b)\rangle| \leq \varepsilon \quad \text{for all} \quad (x,b) \in A \times \{0,1\} \ . \tag{62}$$

Let U be a unitary acting on $L^2(\mathbb{R}^m) \otimes \mathbb{C}^2$ as

$$U\left(|x\rangle \otimes |b\rangle\right) = |x\rangle \otimes |\Omega(x,b)\rangle \qquad \text{for} \qquad (x,b) \in A \times \{0,1\} \ .$$

Let $\ell \in \mathbb{N}_0$ be arbitrary. Then the unitary $I_{L^2(\mathbb{R}^\ell)} \otimes U$ $(6\varepsilon^{1/4})$-approximately computes the function

$$\begin{array}{rccc} \mathsf{id} \times f : & \mathbb{R}^\ell \times A \times \{0,1\} & \to & \mathbb{R}^\ell \times \mathbb{R}^m \times \{0,1\} \\ & (w,x,b) & \mapsto & (w, f(x,b)) = (w, x, h(x) \oplus b) \end{array}$$

on the set $\mathbb{R}^\ell \times A \times \{0,1\}$.

Proof. We first consider the case $\ell = 0$. Let Ψ be a state with $\mathrm{supp}(\Psi) \subseteq A \times \{0,1\}$. Then we have

$$\begin{aligned} U_f |\Psi\rangle &= \sum_{b\in\{0,1\}} \int_{\mathbb{R}^m} dx \Psi(x,b) \, |x\rangle \otimes |h(x) \oplus b\rangle \\ U |\Psi\rangle &= \sum_{b\in\{0,1\}} \int_{\mathbb{R}^m} dx \Psi(x,b) \, |x\rangle \otimes |\Omega(x,b)\rangle \end{aligned}$$

by definition. It follows that

$$\langle U_f \Psi, U\Psi\rangle = \sum_{b,b'\in\{0,1\}} \int_{\mathbb{R}^m} \overline{\Psi(x,b')} \Psi(x,b) \langle h(x) \oplus b', \Omega(x,b)\rangle \ .$$

Separating the pairs $(b,b') \in \{0,1\}^2$ into those where $b = b'$ and those where $b \neq b' = b \oplus 1$, we obtain

$$\langle U_f \Psi, U\Psi\rangle = I_1 + I_2 \tag{63}$$

where

$$\begin{aligned} I_1 &:= \sum_{b\in\{0,1\}} \int_{\mathbb{R}^m} dx \, |\Psi(x,b)|^2 \langle h(x) \oplus b, \Omega(x,b)\rangle \\ I_2 &:= \sum_{b\in\{0,1\}} \int_{\mathbb{R}^m} dx \, \overline{\Psi(x,b\oplus 1)} \Psi(x,b) \langle h(x) \oplus b \oplus 1, \Omega(x,b)\rangle \ . \end{aligned}$$

We can rewrite the term I_1 as an inner product

$$I_1 = \left\langle \sum_{b\in\{0,1\}} \int_{\mathbb{R}^m} dx \Psi(x,b) \, |x\rangle \otimes |b\rangle \otimes |h(x) \oplus b\rangle \, , \sum_{b'\in\{0,1\}} \int_{\mathbb{R}^m} dx' \Psi(x',b') \, |x'\rangle \otimes |b'\rangle \otimes |\Omega(x',b')\rangle \right\rangle \ .$$

By Lemma 5.2 setting $\mathcal{X} = \mathbb{R}^m \times \{0,1\}$, we obtain (by the assumption (62)) that

$$|I_1| \geq 1 - 2\varepsilon \ . \tag{64}$$

Note that because $|\Omega(x,b)\rangle \in \mathbb{C}^2$ has unit norm, we have

$$|\langle h(x) \oplus b, \Omega(x,b)\rangle|^2 + |\langle h(x) \oplus b \oplus 1, \Omega(x,b)\rangle|^2 = 1 \qquad \text{for all} \qquad (x,b) \in A \times \{0,1\} \ .$$

Since $|1 - \langle h(x) \oplus b, \Omega(x,b)\rangle| \leq \varepsilon$ that implies $|\langle \Omega(x,b), h(x) \oplus b\rangle|^2 \geq 1 - 2\varepsilon$, we have

$$|\langle h(x) \oplus b \oplus 1, \Omega(x,b)\rangle| \leq \sqrt{2\varepsilon} \qquad \text{for all} \qquad (x,b) \in A \times \{0,1\} \ .$$

Therefore we obtain

$$|I_2| \leq \sqrt{2\varepsilon} \left(\sum_{b\in\{0,1\}} \int_{\mathbb{R}^m} dx\, |\Psi(x, b\oplus 1)| \cdot |\Psi(x,b)| \right) \ .$$

By the Cauchy-Schwarz inequality applied first to $L^2(\mathbb{R}^m)$ and then to $\mathbb{R}^2$, we can further bound

$$\begin{aligned}
\sum_{b\in\{0,1\}} \int_{\mathbb{R}^m} dx\, |\Psi(x, b\oplus 1)| \cdot |\Psi(x,b)| &\leq \sum_{b\in\{0,1\}} \left(\int_{\mathbb{R}^m} dx\, |\Psi(x, b\oplus 1)|^2 \right)^{1/2} \left(\int_{\mathbb{R}^m} dx\, |\Psi(x,b)|^2 \right)^{1/2} \\
&= \sum_{b\in\{0,1\}} \|\Psi(\cdot, b\oplus 1)\|_{L^2(\mathbb{R}^m)} \|\Psi(\cdot, b)\|_{L^2(\mathbb{R}^m)} \\
&\leq \left(\sum_{b\in\{0,1\}} \|\Psi(\cdot, b)\|^2_{L^2(\mathbb{R}^m)} \right)^{1/2} \left(\sum_{b\in\{0,1\}} \|\Psi(\cdot, b\oplus 1)\|^2_{L^2(\mathbb{R}^m)} \right)^{1/2} \\
&= \sum_{b\in\{0,1\}} \|\Psi(\cdot, b)\|^2_{L^2(\mathbb{R}^m)} \\
&= \|\Psi\|^2 = 1 \ .
\end{aligned}$$

The last step uses the fact that Ψ is normalized. Thus

$$|I_2| \leq \sqrt{2\varepsilon} \ . \tag{65}$$

It follows from (63) combined with (64) and (65) that

$$\begin{aligned}
|\langle U_f \Psi, U\Psi\rangle| &\geq |I_1| - |I_2| \\
&\geq 1 - (2\varepsilon + \sqrt{2\varepsilon}) \ .
\end{aligned}$$

The relation between the overlap and the L^1-norm distance stated in Eq. (43) and the bound $\varepsilon \leq \sqrt{\varepsilon}$ for $0 < \varepsilon \leq 1/2$ yield

$$\|U_f\Psi - U\Psi\|_1 \leq 2\sqrt{2(2\varepsilon + \sqrt{2\varepsilon})} < 6\varepsilon^{1/4} \ .$$

This proves the claim for $\ell = 0$.

Finally, we prove the "stable" version. Let $\ell > 0$. Let us define the function

$$\begin{array}{rccc}
g: & \mathbb{R}^\ell \times A \times \{0,1\} & \to & \mathbb{R}^\ell \times \mathbb{R}^m \times \{0,1\} \\
& (w,x,b) & \mapsto & g(w,x,b) = (w,x,h'(w,x) \oplus b)
\end{array}$$

where $h'(w,x) = h(x)$. The unitary $I_{L^2(\mathbb{R}^\ell)} \otimes U$ acts as $(I_{L^2(\mathbb{R}^\ell)} \otimes U)\,(|w\rangle \otimes |x\rangle \otimes |b\rangle) = |w\rangle \otimes |x\rangle \otimes |\Omega'(w,x,b)\rangle$, where $|\Omega'(w,x,b)\rangle := |\Omega(x,b)\rangle$. Setting $A' := \mathbb{R}^\ell \times A$, we have

$$|1 - \langle h'(w,x) \oplus b, \Omega'(w,x,b)\rangle| \leq \varepsilon \qquad \text{for all} \qquad (w,x,b) \in A' \times \{0,1\} \ ,$$

Thus the argument for $\ell > 0$ reduces to the one for the case $\ell = 0$. □

5.4 Approximate function evaluation by various building blocks

The analysis of the circuit $\mathcal{Q}_{a,N}$ relies on our framework for approximate function evaluation: We repeatedly apply Lemma 5.1 to evaluate how certain composed unitaries act on approximate position-eigenstates.

5.4.1 Approximate evaluation of the least significant bit extraction

Consider the following unitary $U^{\mathsf{LSB}}_{\mathrm{A}\to\mathrm{Q}}$ acting on a single bosonic mode A and a qubit Q.

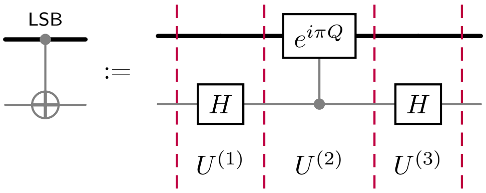

This unitary is decomposed as

$$U^{\mathsf{LSB}}_{\mathrm{A}\to\mathrm{Q}} = U^{(3)}U^{(2)}U^{(1)} \qquad \text{where} \qquad \begin{aligned} U^{(1)} &= I_{\mathrm{A}} \otimes H_{\mathrm{Q}} \\ U^{(2)} &= \mathsf{ctrl}_{\mathrm{Q}} e^{i\pi Q_{\mathrm{A}}} \\ U^{(3)} &= I_{\mathrm{A}} \otimes H_{\mathrm{Q}} \ . \end{aligned} \tag{66}$$

In Section 1.1.4 we argued that when applied to a position-eigenstate $|x\rangle$ with an integer position $x \in \mathbb{Z}$ and a qubit in a computational basis state $\{|0\rangle, |1\rangle\}$, the unitary $U^{\mathsf{LSB}}_{\mathrm{A}\to\mathrm{Q}}$ computes the LSB of x into the qubit. That is, it acts as

$$U^{\mathsf{LSB}}_{\mathrm{A}\to\mathrm{Q}}\left(|x\rangle_{\mathrm{A}} \otimes |b\rangle_{\mathrm{Q}}\right) = |x\rangle_{\mathrm{A}} \otimes |x_0 \oplus b\rangle_{\mathrm{Q}} \qquad \text{for all} \qquad x \in \mathbb{Z} \text{ and } b \in \{0,1\} \ ,$$

where we used $x_0 = x \bmod 2$ to denote the least significant bit of the integer $x \in \mathbb{Z}$.

We now consider the non-ideal case, where the input state is a position-eigenstate $|x\rangle$ such that $x \in \mathbb{R}$ is not necessarily an integer, but is close to an integer up to an error $\varepsilon < 1/2$, that is, $\min_{y\in\mathbb{Z}} |x-y| \leq \varepsilon$.

We will show that $U^{\mathsf{LSB}}_{\mathrm{A}\to\mathrm{Q}}$ approximately computes the LSB $\lfloor x \rceil_0$ of the rounded number $\lfloor x \rceil \in \mathbb{Z}$. Here we denote by $\lfloor x \rceil \in \mathbb{Z}$ the closest integer to $x \in \mathbb{R}$ (for $x = x' + 1/2$, where $x' \in \mathbb{Z}$, we set $\lfloor x \rceil = x' + 1$). Recall that for $\varepsilon > 0$ and a set $M \subseteq \mathbb{Z}$, we define the set

$$M(\varepsilon) := \{x \in \mathbb{R} \mid \exists y \in M : |x-y| \leq \varepsilon\} \ ,$$

which is the Minkowski sum $M(\varepsilon) = M + [-\varepsilon, \varepsilon]$ of the set M and the interval $[-\varepsilon, \varepsilon]$.

The key property of the unitary $U^{\mathsf{LSB}}_{\mathrm{A}\to\mathrm{Q}}$ we use is the following.

Lemma 5.4. *Let $U^{\mathsf{LSB}}_{\mathrm{A}\to\mathrm{Q}}$ be defined by Eq.* (66)*. Let $(x,b) \in \mathbb{Z}(\varepsilon) \times \{0,1\}$ be arbitrary. Then there is a state $|\Omega(x,b)\rangle \in \mathbb{C}^2$ such that*

$$U^{\mathsf{LSB}}_{\mathrm{A}\to\mathrm{Q}}\left(|x\rangle_{\mathrm{A}} \otimes |b\rangle_{\mathrm{Q}}\right) = |x\rangle_{\mathrm{A}} \otimes |\Omega(x,b)\rangle_{\mathrm{Q}} \qquad \textit{and} \qquad |1 - \langle \lfloor x \rceil_0 \oplus b, \Omega(x,b)\rangle| \leq \frac{\varepsilon\pi}{2} \ .$$

Similarly, there is a state $|\Omega'(x,b)\rangle$ such that

$$\left(U^{\mathsf{LSB}}_{\mathrm{A}\to\mathrm{Q}}\right)^{\dagger}\left(|x\rangle_{\mathrm{A}} \otimes |b\rangle_{\mathrm{Q}}\right) = |x\rangle_{\mathrm{A}} \otimes |\Omega'(x,b)\rangle_{\mathrm{Q}} \qquad \textit{and} \qquad |1 - \langle \lfloor x \rceil_0 \oplus b, \Omega'(x,b)\rangle| \leq \frac{\varepsilon\pi}{2}.$$

Lemma 5.4 shows that the unitary $U^{\mathsf{LSB}}_{A\to Q}$ satisfies the sufficient condition for approximate function evaluation given in Lemma 5.3. We can therefore conclude that $U^{\mathsf{LSB}}_{A\to Q}$ computes the LSB of the position x of mode A even if x is only ε-close to an integer, for $\varepsilon \in [0, 1/2)$. We argue that this is the case even for a "stabilized" version of $U^{\mathsf{LSB}}_{A\to Q}$, i.e., when additional modes are involved, see Corollary 5.2.

Proof. By definition, $U^{\mathsf{LSB}}_{A\to Q}$ consists of three unitaries $U^{\mathsf{LSB}}_{A\to Q} = U^{(3)}U^{(2)}U^{(1)}$ (see Eq. (66)). We apply this to the state $|x\rangle_A \otimes |b\rangle_Q$. After applying the first unitary $U^{(1)} = I_A \otimes H_Q$ (i.e., a Hadamard on the qubit) we have

$$\begin{aligned} U^{(1)}\left(|x\rangle_A \otimes |b\rangle_Q\right) &= |x\rangle_A \otimes \left(H_Q\, |b\rangle_Q\right) \\ &= |x\rangle_A \otimes \frac{1}{\sqrt{2}}\left(|0\rangle_Q + e^{-i\pi b}\, |1\rangle_Q\right) \ . \end{aligned}$$

Second, we apply $U^{(2)} = I_B \otimes |0\rangle\langle 0|_Q + e^{i\pi Q_A} \otimes |1\rangle\langle 1|_Q$ and get

$$U^{(2)}U^{(1)}\left(|x\rangle_A \otimes |b\rangle_Q\right) = |x\rangle_A \otimes \frac{1}{\sqrt{2}}\left(|0\rangle_Q + e^{i\pi(x-b)}\, |1\rangle_Q\right) \ .$$

Finally, application of the unitary $U^{(3)} = I_A \otimes H_Q$ leads to

$$\begin{aligned} U^{(3)}U^{(2)}U^{(1)}\left(|x\rangle_A \otimes |b\rangle_Q\right) &= |x\rangle_A \otimes \frac{1}{\sqrt{2}}\left(|+\rangle_Q + e^{i\pi(x-b)}\, |-\rangle_Q\right) \\ &= |x\rangle_A \otimes \frac{1}{2}\left(\left(1 + e^{i\pi(x-b)}\right)|0\rangle_Q + \left(1 - e^{i\pi(x-b)}\right)|1\rangle_Q\right) \\ &= |x\rangle_A \otimes |\Omega(x,b)\rangle_Q \end{aligned}$$

where we introduced the single-qubit state

$$|\Omega(x,b)\rangle = \frac{1}{2}\left(\left(1 + e^{i\pi(x-b)}\right)|0\rangle_Q + \left(1 - e^{i\pi(x-b)}\right)|1\rangle_Q\right) \ .$$

Since we can write

$$|\lfloor x\rceil_0 \oplus b\rangle = \frac{1}{2}\left((1 + e^{i\pi(\lfloor x\rceil_0 + b)})\,|0\rangle + (1 - e^{i\pi(\lfloor x\rceil_0 + b)})\,|1\rangle\right) \ , \tag{67}$$

the overlap of the state $|\Omega(x,b)\rangle$ and the state $|\lfloor x\rceil_0 \oplus b\rangle$ is

$$\begin{aligned} \langle \lfloor x\rceil_0 \oplus b, \Omega(x,b)\rangle &= \frac{1}{2}\left(1 + e^{i\pi(x-\lfloor x\rceil_0 - 2b)}\right) \\ &= \frac{1}{2}\left(1 + e^{i\pi(x-\lfloor x\rceil_0)}\right) \\ &= \frac{1}{2}\left(1 + e^{i\pi(x-\lfloor x\rceil_0)} \cdot e^{-i\pi(\lfloor x\rceil - \lfloor x\rceil_0)}\right) \\ &= \frac{1}{2}\left(1 + e^{i\pi\,\mathrm{dev}(x)}\right) \ , \end{aligned}$$

where we used that $\lfloor x\rceil - \lfloor x\rceil_0$ is even to obtain the penultimate step and where $\mathrm{dev}(x) := x - \lfloor x\rceil$ is the deviation from the closest integer. We are interested in $(x,b) \in \mathbb{Z}(\varepsilon) \times \{0,1\}$. By definition $|\mathrm{dev}(x)| \leq \varepsilon$ for $x \in \mathbb{Z}(\varepsilon)$ thus we have

$$|1 - \langle \lfloor x\rceil_0 \oplus b, \Omega(x,b)\rangle| = \left|\frac{1 - e^{i\pi\,\mathrm{dev}(x)}}{2}\right| = \left|\sin\frac{\pi\,\mathrm{dev}(x)}{2}\right| \leq \left|\sin\frac{\pi\varepsilon}{2}\right| \leq \frac{\pi\varepsilon}{2} \ , \tag{68}$$

where we used the identity $\left|\frac{1-e^{ix}}{2}\right| = \left|\sin\frac{x}{2}\right|$, the fact that $x \mapsto \sin x$ is odd on $\mathbb{R}$ and monotonically increasing on $[0,\pi/2]$, and $|\sin x| \leq |x|$ for all $x \in \mathbb{R}$. This concludes the proof of the claim for the unitary $U^{\mathsf{LSB}}_{\mathrm{A}\to\mathrm{Q}}$.

Now consider the adjoint $(U^{\mathsf{LSB}}_{\mathrm{A}\to\mathrm{Q}})^\dagger = (U^{(1)})^\dagger(U^{(2)})^\dagger(U^{(3)})^\dagger$. By the same line of reasoning as for $U^{\mathsf{LSB}}_{\mathrm{A}\to\mathrm{Q}}$ we obtain

$$\begin{aligned}(U^{(3)})^\dagger(U^{(2)})^\dagger(U^{(1)})^\dagger\left(|x\rangle_\mathrm{A}\otimes|b\rangle_\mathrm{Q}\right) &= |x\rangle_\mathrm{A}\otimes\frac{1}{2}\left(\left(1+e^{-i\pi(x+b)}\right)|0\rangle_\mathrm{Q}+\left(1-e^{-i\pi(x+b)}\right)|1\rangle_\mathrm{Q}\right)\\ &= |x\rangle_\mathrm{A}\otimes\left|\Omega'(x,b)\right\rangle_\mathrm{Q}\ ,\end{aligned}$$

where

$$\left|\Omega'(x,b)\right\rangle = \frac{1}{2}\left(\left(1+e^{-i\pi(x+b)}\right)|0\rangle_\mathrm{Q}+\left(1-e^{-i\pi(x+b)}\right)|1\rangle_\mathrm{Q}\right)\ .$$

By (67) the overlap satisfies

$$\begin{aligned}\langle\lfloor x\rceil_0\oplus b,\Omega'(x,b)\rangle &= \frac{1}{2}\left(1+e^{-i\pi(x+\lfloor x\rceil_0)}\right)\\ &= \frac{1}{2}\left(1+e^{-i\pi(x+\lfloor x\rceil_0)}\cdot e^{i\pi(\lfloor x\rceil+\lfloor x\rceil_0)}\right)\\ &= \frac{1}{2}\left(1+e^{-i\pi\,\mathrm{dev}(x)}\right)\ ,\end{aligned}$$

where we obtained the second equality by noting that $\lfloor x\rceil + \lfloor x\rceil_0$ is even for $x \in \mathbb{R}$. It follows (as in Eq. (68)) that $|1-\langle\lfloor x\rceil_0\oplus b,\Omega'(x,b)\rangle| \leq \frac{\varepsilon\pi}{2}$ for $(x,b)\in\mathbb{Z}(\varepsilon)\times\{0,1\}$. □

Corollary 5.2. *Let $\varepsilon\in(0,1/2)$ and let $\ell\in\mathbb{N}_0$. Consider the unitary $U = I_\mathrm{A}\otimes U^{\mathsf{LSB}}_{\mathrm{B}\to\mathrm{Q}}$, where* A *denotes an ℓ-mode system,* B *is a single mode, and* Q *denotes a qubit system. Then*

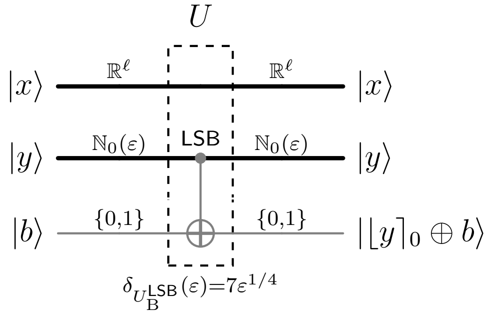

in the diagrammatic notation (cf. (58)). Furthermore, the unitary $U^\dagger = I_\mathrm{A}\otimes(U^{\mathsf{LSB}}_{\mathrm{B}\to\mathrm{Q}})^\dagger$ satisfies

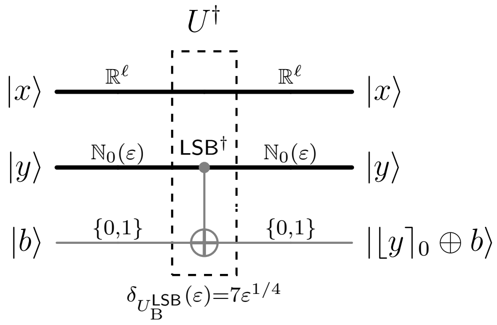

Proof. Both claims follow directly from Lemma 5.3 applied to Lemma 5.4 along with the fact that $6(\pi/2)^{1/4}\leq 7$. □

5.4.2 Approximate function evaluation by the unitaries V_α, $V_{a,N,m}$, and $U_{a,N,m}$

In this section we use our framework for approximate function evaluation to analyze the composed unitaries V_α, $V_{a,N,m}$, and $U_{a,N,m}$ used in our algorithm.

Let us recall the unitary V_α defined in Figure 2 of the main text for $\alpha > 0$. It is given by the circuit

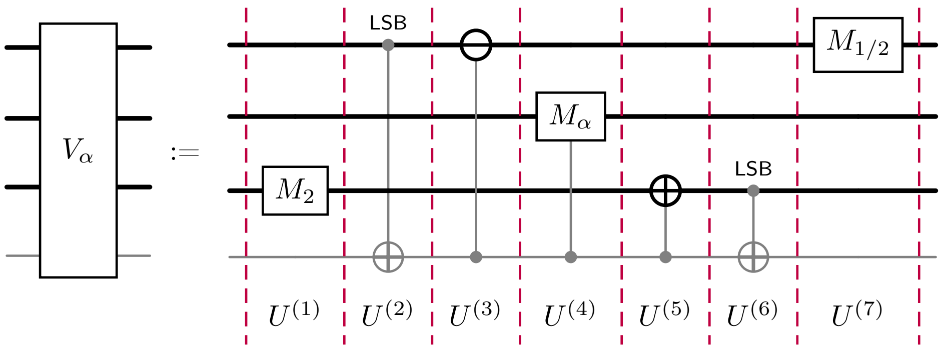

(69)

i.e.,

$$(V_\alpha)_{\mathrm{ABCQ}} = U^{(7)}U^{(6)}U^{(5)}U^{(4)}U^{(3)}U^{(2)}U^{(1)}$$

where

$$\begin{aligned} U^{(1)} &= (M_2)_{\mathrm{C}} & U^{(5)} &= \mathsf{ctrl}_{\mathrm{Q}} e^{-iP_{\mathrm{C}}} \\ U^{(2)} &= U^{\mathsf{LSB}}_{\mathrm{A}\to\mathrm{Q}} & U^{(6)} &= U^{\mathsf{LSB}}_{\mathrm{C}\to\mathrm{Q}} \\ U^{(3)} &= \mathsf{ctrl}_{\mathrm{Q}} e^{iP_{\mathrm{A}}} & U^{(7)} &= (M_{2^{-1}})_{\mathrm{A}} \ . \\ U^{(4)} &= \mathsf{ctrl}_{\mathrm{Q}}(M_\alpha)_{\mathrm{B}} \end{aligned} \tag{70}$$

We depict the actions of the constituting unitaries on inputs that are close to integers on the modes A and C by the diagrams in Supplementary Figure 5 (cf. (58) for an explanation of the diagrammatic notation). These actions are obtained directly from the definitions of the respective unitaries and from Corollary 5.2 for the LSB gates (by relabeling modes).

Using the elements expressed by Supplementary Figure 5, we can show the following:

Lemma 5.5. *Let $\varepsilon_{\mathrm{A}} \in (0, 1/2)$ and $\varepsilon_{\mathrm{C}} \in (0, 1/4)$. Let $\alpha > 0$. Consider the unitary V_α defined by the circuit in Figure 2 of the main text. Then*

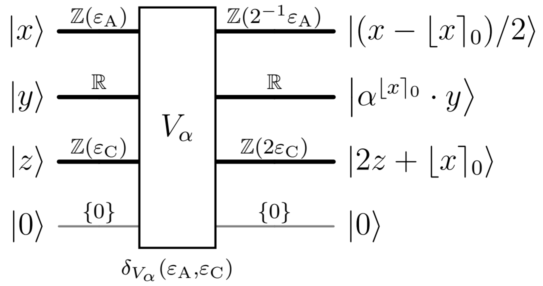

where $\delta_{V_\alpha}(\varepsilon_{\mathrm{A}}, \varepsilon_{\mathrm{C}}) = 7\varepsilon_{\mathrm{A}}^{1/4} + 7(2\varepsilon_{\mathrm{C}})^{1/4}$.

We note that the error bound $\delta_{V_\alpha}(\varepsilon_{\mathrm{A}}, \varepsilon_{\mathrm{C}})$ does not depend on the parameter α of the unitary V_α. Below we will only use the action of V_α restricted to inputs close to non-negative integers

in modes A and C, i.e.,

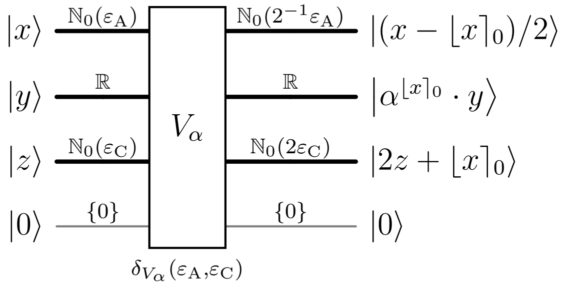

(71)

This is well-defined since $x\in\mathbb{N}_0(\varepsilon_{\mathrm{A}})$ implies that $(x-\lfloor x\rceil_0)/2\in\mathbb{N}_0(2^{-1}\varepsilon_{\mathrm{A}})$, and similarly, $z\in\mathbb{N}_0(\varepsilon_{\mathrm{A}})$ implies that $2z+\lfloor x\rceil_0\in\mathbb{N}_0(2\varepsilon_{\mathrm{C}})$ for any $x\in\mathbb{N}_0$.

Proof of Lemma 5.5. Let us write $V^{(T)}=U^{(T)}\cdots U^{(1)}$ for $T\in\{2,\ldots,7\}$. We obtained the approximate action of each unitary $V^{(T)}$ by Lemma 5.1 and list them in Supplementary Figure 6. The claim follows by noting that $V^{(7)}=V_\alpha$ and by setting (see Supplementary Figure 6f)

$$\begin{aligned}\delta_{V_\alpha}&=\delta_{U_{\mathrm{A}}^{\mathsf{LSB}}}(\varepsilon_{\mathrm{A}})+\delta_{U_{\mathrm{C}}^{\mathsf{LSB}}}(2\varepsilon_{\mathrm{C}})\\&=7\varepsilon_{\mathrm{A}}^{1/4}+7(2\varepsilon_{\mathrm{C}})^{1/4}\ .\end{aligned}$$

□

We also need the approximate action of the unitary $V_\alpha^\dagger$.

Lemma 5.6. *Let $\varepsilon_{\mathrm{A}}\in(0,1/4)$ and $\varepsilon_{\mathrm{C}}\in(0,1/2)$. Let $\alpha>0$. Consider the unitary $V_\alpha^\dagger$, we have*

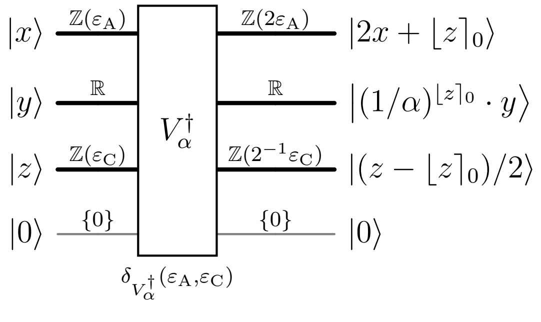

where $\delta_{V_\alpha^\dagger}(\varepsilon_{\mathrm{A}},\varepsilon_{\mathrm{C}})=7(2\varepsilon_{\mathrm{A}})^{1/4}+7\varepsilon_{\mathrm{C}}^{1/4}$.

Again, the error bound $\delta_{V_\alpha^\dagger}(\varepsilon_{\mathrm{A}},\varepsilon_{\mathrm{C}})$ does not depend on the parameter α. We will only need the action of $V_\alpha^\dagger$ restricted to inputs close to non-negative integers in modes A and C, i.e.

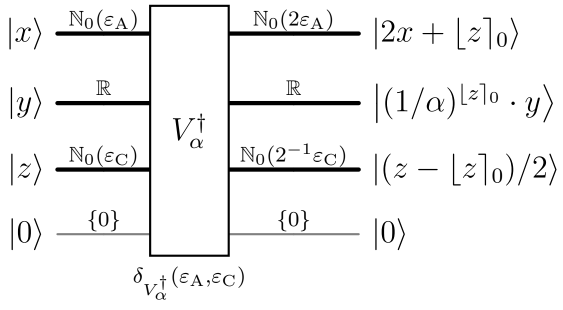

(72)

This action is well-defined since $x\in\mathbb{N}_0(\varepsilon_{\mathrm{A}})$ implies that $2x+\lfloor z\rceil_0\in\mathbb{N}_0(2\varepsilon_{\mathrm{A}})$ for any $z\in\mathbb{N}_0(\varepsilon_{\mathrm{C}})$, and $z\in\mathbb{N}_0(\varepsilon_{\mathrm{C}})$ implies that $(z-\lfloor z\rceil_0)/2\in\mathbb{N}_0(2^{-1}\varepsilon_{\mathrm{C}})$.

Proof of Lemma 5.6. The adjoint of V_α from Eq. (69) is

$$V_\alpha^\dagger = (U^{(1)})^\dagger \cdots (U^{(7)})^\dagger$$

where the unitaries $U^{(1)}, \ldots, U^{(7)}$ are defined in Eq. (70). The approximate action of the unitaries decomposing $V_\alpha^\dagger$ is given in Supplementary Figure 7. We will use Lemma 5.1 to derive the composed action. Let us write $(V')^{(T)} = (U^{(7-T+1)})^\dagger \cdots (U^{(7)})^\dagger$ for $T \in \{2, \ldots, 7\}$. Each $(V')^{(T)}$ is a unitary composed of the first T unitaries from the decomposition of $V_\alpha^\dagger$. The approximate action of each of them is given in Supplementary Figure 8. The claim follows from $(V')^{(7)} = V_\alpha^\dagger$ and by setting $\delta_{V_\alpha^\dagger}(\varepsilon_\mathrm{A}, \varepsilon_\mathrm{C}) = \delta_{U_\mathrm{C}^{\mathtt{LSB}}}(\varepsilon_\mathrm{C}) + \delta_{U_\mathrm{A}^{\mathtt{LSB}}}(2\varepsilon_\mathrm{A}) = 7(2\varepsilon_\mathrm{A})^{1/4} + 7\varepsilon_\mathrm{C}^{1/4}$. □

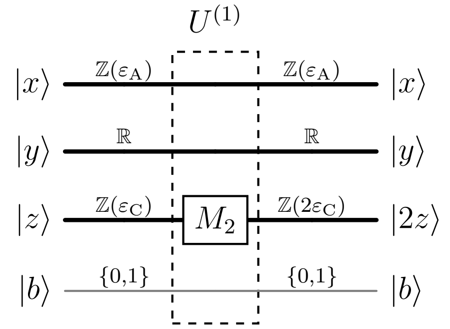

(a) Action of the unitary $U^{(1)}$.

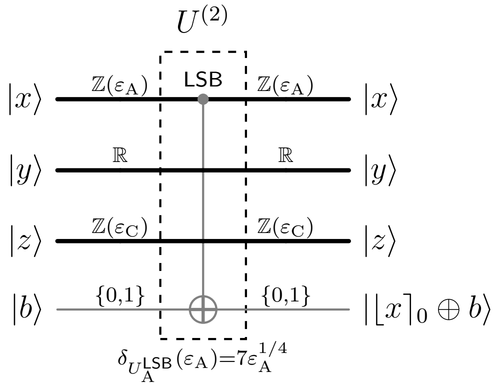

(b) Action of $U^{(2)}$ (obtained by Corollary 5.2).

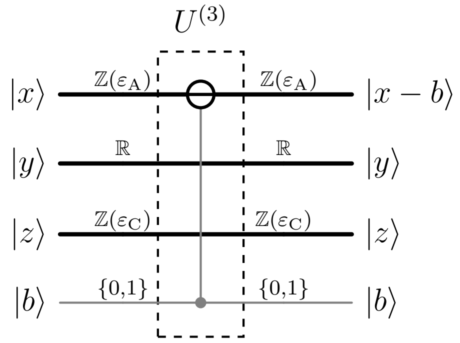

(c) Action of the unitary $U^{(3)}$.

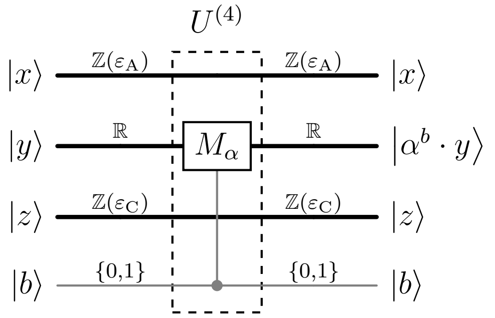

(d) Action of the unitary $U^{(4)}$.

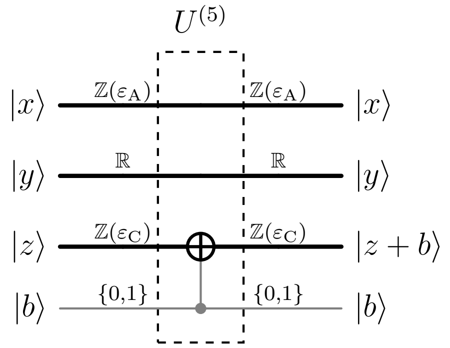

(e) Action of the unitary $U^{(5)}$.

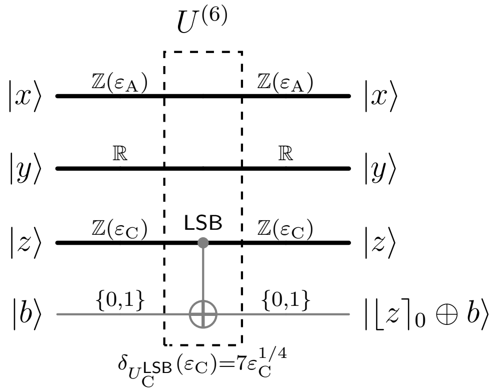

(f) Action of $U^{(6)}$ (obtained by Corollary 5.2).

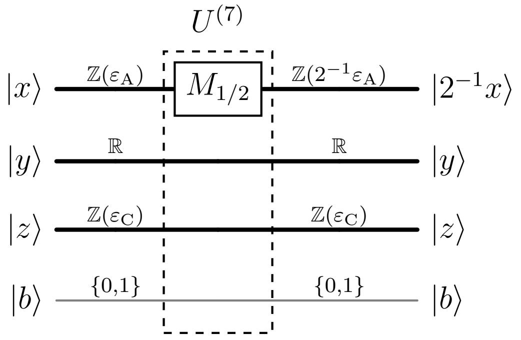

(g) Action of the unitary $U^{(7)}$.

Supplementary Figure 5. The approximate action of the unitaries decomposing $V_\alpha = U^{(7)}U^{(6)} \cdots U^{(1)}$ displayed in the diagramatic notation (58) of our approximate function evaluation framework described in Section 5.2.

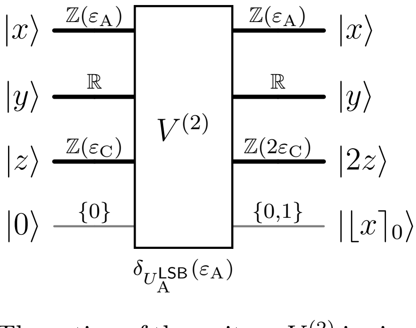

(a) The action of the unitary $V^{(2)}$ is given by composition of $U^{(1)}$ and $U^{(2)}$ from Figs. 5a and 5b, respectively.

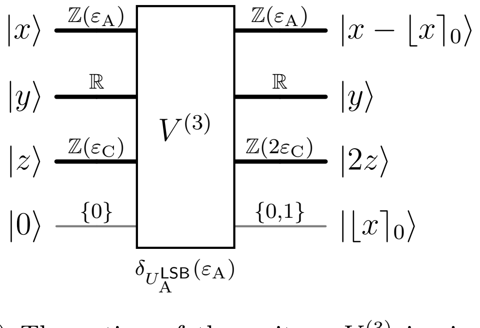

(b) The action of the unitary $V^{(3)}$ is given by composition of $V^{(2)}$ from Supplementary Figure 6a with $U^{(3)}$ from Supplementary Figure 5c.

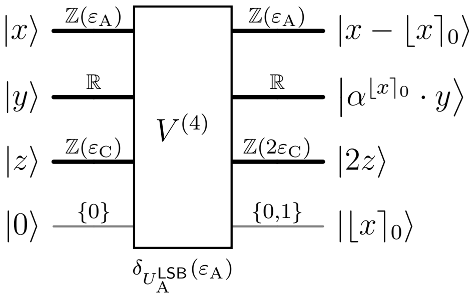

(c) The action of $V^{(4)}$ is given by composing $V^{(3)}$ from Figure 6b with $U^{(4)}$ from Supplementary Figure 5d.

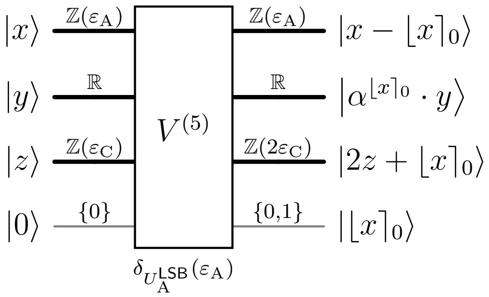

(d) The action of $V^{(5)}$ is given by composing $V^{(4)}$ from Supplementary Figure 6c with $U^{(5)}$ from Supplementary Figure 5e.

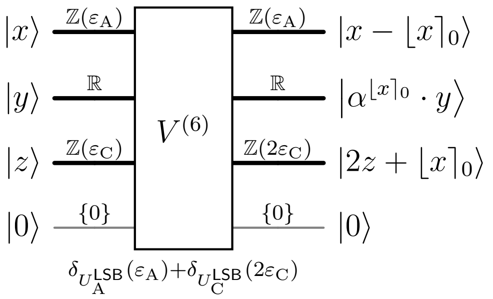

(e) The action of $V^{(6)}$ is given by composing the unitary $V^{(5)}$ from Supplementary Figure 6d with the unitary $U^{(6)}$ from Supplementary Figure 5f and by noting that $\lfloor 2z + \lfloor x \rceil_0 \rceil_0 = \lfloor 2z \rceil_0 \oplus \lfloor x \rceil_0 = \lfloor x \rceil_0$ for any $z \in \mathbb{Z}(\varepsilon_{\mathrm{C}})$ such that $2\varepsilon_{\mathrm{C}} < 1/2$.

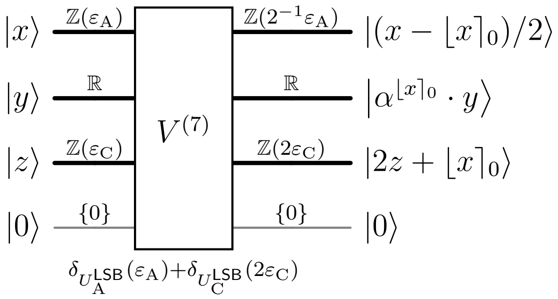

(f) The action of the unitary $V^{(7)}$ is given by composing $V^{(6)}$ from Supplementary Figure 6e with $U^{(7)}$ from Supplementary Figure 5g.

Supplementary Figure 6. Action of the unitaries $V^{(1)}, \ldots, V^{(7)}$ from the decomposition of the unitary V_α obtained by repeated application of Lemma 5.1.

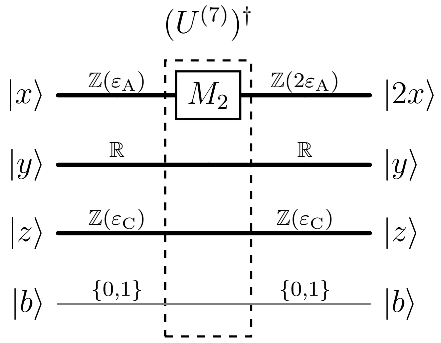

(a) Action of the unitary $(U^{(7)})^\dagger$.

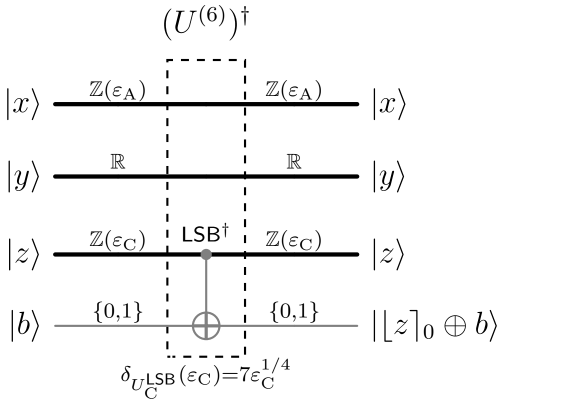

(b) Action of $(U^{(6)})^\dagger$ (by Corollary 5.2).

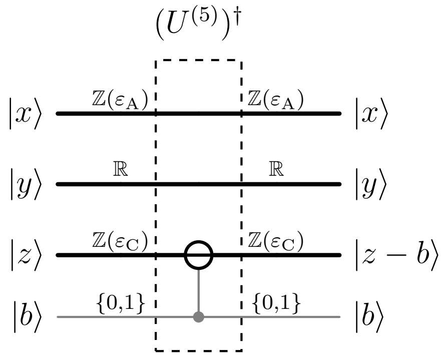

(c) Action of the unitary $(U^{(5)})^\dagger$.

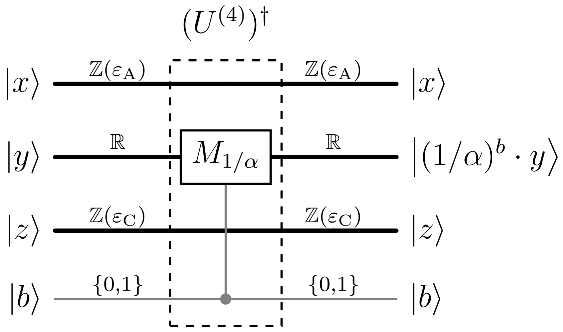

(d) Action of the unitary $(U^{(4)})^\dagger$.

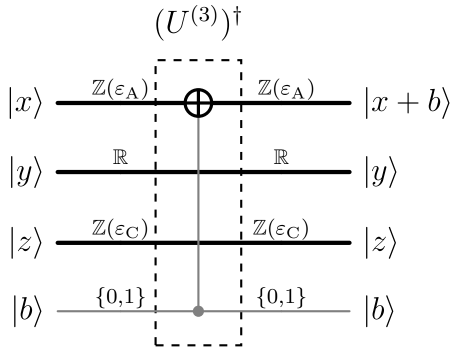

(e) Action of the unitary $(U^{(3)})^\dagger$.

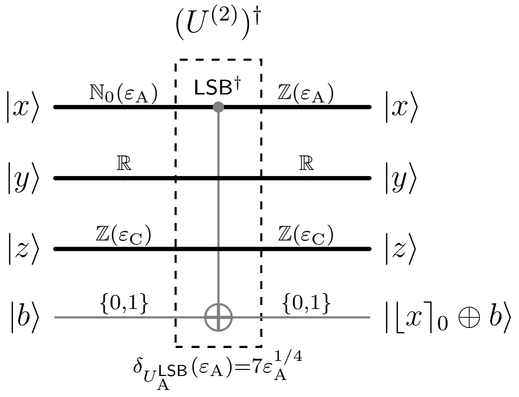

(f) Action of $(U^{(2)})^\dagger$ (by Corollary 5.2).

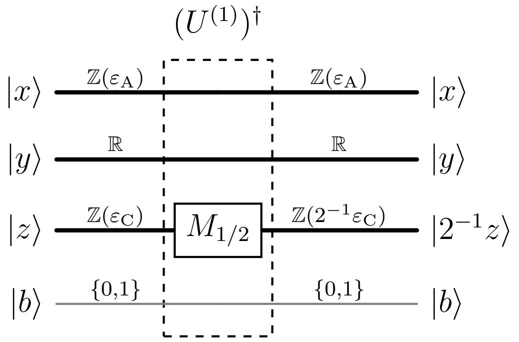

(g) Action of the unitary $(U^{(1)})^\dagger$.

Supplementary Figure 7. The approximate action of the unitaries decomposing $V_\alpha^\dagger = (U^{(1)})^\dagger (U^{(2)})^\dagger \cdots (U^{(7)})^\dagger$ in the diagramatic notation (58). The unitaries are ordered in subfigures in the order they are applied in the circuit $V_\alpha^\dagger$.

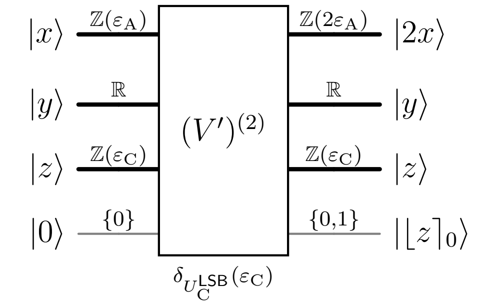

(a) The action of the unitary $(V')^{(2)}$ is a composition of $(U^{(7)})^\dagger$ with $(U^{(6)})^\dagger$ from Figs. 7a and 7b, respectively.

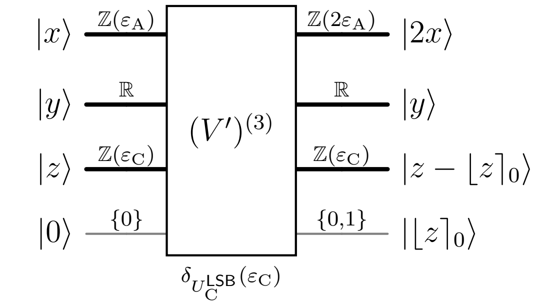

(b) The action of the unitary $(V')^{(3)}$ is given by composing $(V')^{(2)}$ from Supplementary Figure 8a with $(U^{(5)})^\dagger$ from Supplementary Figure 7c.

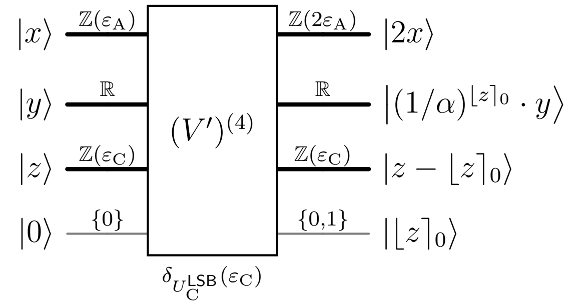

(c) The action of $(V')^{(4)}$ is given by composing $(V')^{(3)}$ from Supplementary Figure 8b with $(U^{(4)})^\dagger$ from Supplementary Figure 7d.

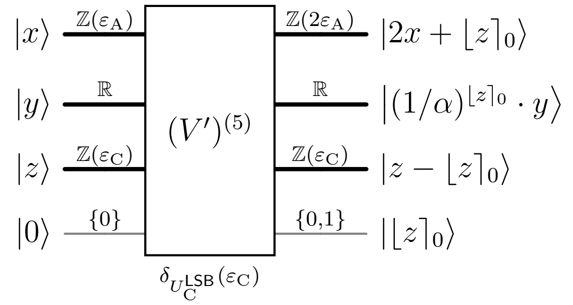

(d) The action of $(V')^{(5)}$ is given by composing $(V')^{(4)}$ from Supplementary Figure 8c with $(U^{(3)})^\dagger$ from Supplementary Figure 7e.

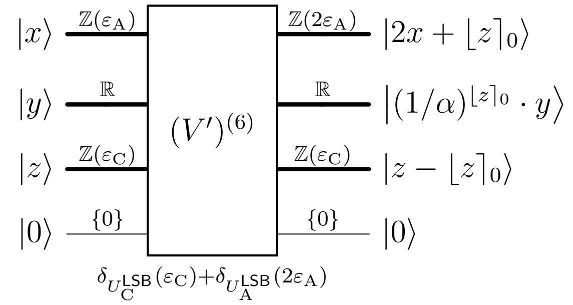

(e) The action of $(V')^{(6)}$ is given by composing $(V')^{(5)}$ from Supplementary Figure 8d with the unitary $(U^{(2)})^\dagger$ from Supplementary Figure 7f and by the fact that $\lfloor 2x + \lfloor z \rceil_0 \rceil_0 = \lfloor 2x \rceil_0 \oplus \lfloor z \rceil_0 = \lfloor z \rceil_0$ for any $x \in \mathbb{Z}(\varepsilon_{\mathrm{C}})$ with $2\varepsilon_{\mathrm{C}} < 1/2$.

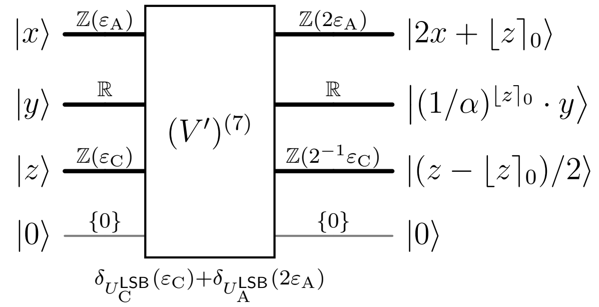

(f) The action of $(V')^{(7)}$ is given by composing $(V')^{(6)}$ from Supplementary Figure 8e with $(U^{(1)})^\dagger$ from Supplementary Figure 7g.

Supplementary Figure 8. Actions of the unitaries $(V')^{(1)}, \ldots, (V')^{(7)}$ from the decomposition of $V_\alpha^\dagger$. These actions were obtained in a similar way as $V^{(1)}, \ldots, V^{(7)}$ from the decomposition of V_α in Supplementary Figure 6 (i.e., by Lemma 5.1).

To analyze the unitary $V_{a,N,m}$ and its adjoint it is useful to first prove the following statement.

Lemma 5.7. *Let $m \in \mathbb{N}$ and $\vec{\alpha} = (\alpha_1, \ldots, \alpha_m) \in (0,\infty)^m$ and $\vec{\beta} = (\beta_1, \ldots, \beta_m) \in (0,\infty)^m$. Define the unitary*

$$V(\vec{\beta}, \vec{\alpha}) = V_{\beta_m}^\dagger \cdots V_{\beta_1}^\dagger \cdot V_{\alpha_m} \cdots V_{\alpha_1}$$

and the functions

$$f_{\vec{\alpha}}: \begin{array}{ccc} \mathbb{N}_0 & \to & \mathbb{R} \\ x & \mapsto & \prod_{i=1}^{m} \alpha_i^{x_{i-1}} \end{array} \quad \text{and} \quad g_{\vec{\beta}}: \begin{array}{ccc} \mathbb{N}_0 & \to & \mathbb{R} \\ x & \mapsto & \prod_{i=1}^{m} (1/\beta_i)^{x_{m-i}} \end{array} ,$$

where $x = \sum_{i=0} 2^i x_i$*. Let* $\varepsilon_{\mathrm{A}} \in (0, 1/4)$ *and let* $\varepsilon_{\mathrm{C}} \in (0, 2^{-(m+2)})$*. Then*

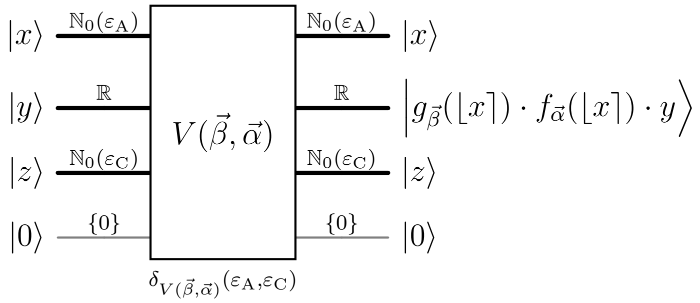

where $\delta_{V(\vec{\beta},\vec{\alpha})}(\varepsilon_{\mathrm{A}}, \varepsilon_{\mathrm{C}}) = 88 \cdot \varepsilon_{\mathrm{A}}^{1/4} + 88 \cdot (2^m \varepsilon_{\mathrm{C}})^{1/4}$*.*

Proof. We will use Lemma 5.1 to obtain the approximate action of $V(\vec{\beta}, \vec{\alpha})$ from the approximate actions of its constituent unitaries. Set

$$(x^{(0)}, y^{(0)}, z^{(0)}, \varepsilon_{\mathrm{A}}^{(0)}, \varepsilon_{\mathrm{C}}^{(0)}) = (x, y, z, \varepsilon_{\mathrm{A}}, \varepsilon_{\mathrm{C}}) \ .$$

By Lemma 5.5 (that is, Eq. (71)) we have

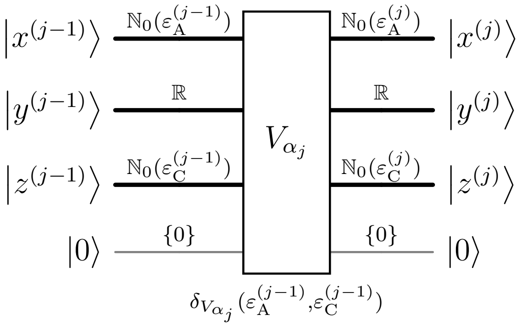

where

$$\begin{array}{rcl} x^{(j)} & = & (x^{(j-1)} - \lfloor x^{(j-1)} \rceil_0)/2 \\ y^{(j)} & = & \alpha_j^{\lfloor x^{(j-1)} \rceil_0} \cdot y^{(j-1)} \\ z^{(j)} & = & 2z^{(j-1)} + \lfloor x^{(j-1)} \rceil_0 \end{array} \quad \text{and} \quad \begin{array}{rcl} \varepsilon_{\mathrm{A}}^{(j)} & = & 2^{-1}\varepsilon_{\mathrm{A}}^{(j-1)} \\ \varepsilon_{\mathrm{C}}^{(j)} & = & 2\varepsilon_{\mathrm{C}}^{(j-1)} \end{array} \tag{73}$$

for any $j \in \{1, \ldots, m\}$. Note that Lemma 5.5 is applicable here since $\varepsilon_{\mathrm{A}}^{(j)} = 2^{-j}\varepsilon_{\mathrm{A}}$, $\varepsilon_{\mathrm{C}}^{(j)} = 2^j \varepsilon_{\mathrm{C}}$ and $2^{-j}\varepsilon_{\mathrm{A}} < 1/4$, $2^j \varepsilon_{\mathrm{C}} < 1/4$ by the assumptions on ε_{A} and ε_{C}, for all $j \in \{1, \ldots, m\}$. It follows inductively that

$$\begin{array}{rcl} x^{(m)} & = & 2^{-m}x - \sum_{j=0}^{m-1} 2^{-m+j} \lfloor x \rceil_j \\ y^{(m)} & = & \left(\prod_{j=1}^{m} \alpha_j^{\lfloor x \rceil_{j-1}} \right) \cdot y = f_{\vec{\alpha}}(\lfloor x \rceil) \cdot y \\ z^{(m)} & = & 2^m z + \sum_{j=0}^{m-1} 2^{m-1-j} \lfloor x \rceil_j \end{array} \quad \text{and} \quad \begin{array}{rcl} \varepsilon_{\mathrm{A}}^{(m)} & = & 2^{-m}\varepsilon_{\mathrm{A}} \\ \varepsilon_{\mathrm{C}}^{(m)} & = & 2^m \varepsilon_{\mathrm{C}} \end{array} \tag{74}$$

Let us set

$$(\tilde{x}^{(0)}, \tilde{y}^{(0)}, \tilde{z}^{(0)}, \tilde{\varepsilon}_{\mathrm{A}}^{(0)}, \tilde{\varepsilon}_{\mathrm{C}}^{(0)}) = (x^{(m)}, y^{(m)}, z^{(m)}, \varepsilon_{\mathrm{A}}^{(m)}, \varepsilon_{\mathrm{C}}^{(m)}) \ . \tag{75}$$

By Lemma 5.6 (more precisely, Eq. (72)), we have

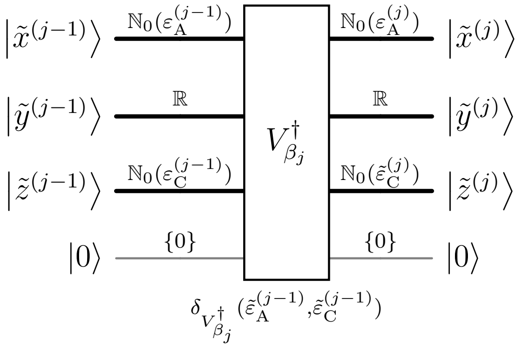

where

$$\begin{aligned}\tilde{x}^{(j)} &= 2\tilde{x}^{(j-1)} + \lfloor \tilde{z}^{(j-1)} \rceil_0 \\ \tilde{y}^{(j)} &= (1/\beta_j)^{\lfloor \tilde{z}^{(j-1)} \rceil_0} \cdot \tilde{y}^{(j-1)} \\ \tilde{z}^{(j)} &= (\tilde{z}^{(j-1)} - \lfloor \tilde{z}^{(j-1)} \rceil_0)/2\end{aligned} \qquad \text{and} \qquad \begin{aligned}\tilde{\varepsilon}_{\mathrm{A}}^{(j)} &= 2\tilde{\varepsilon}_{\mathrm{A}}^{(j-1)} \\ \tilde{\varepsilon}_{\mathrm{C}}^{(j)} &= 2^{-1}\tilde{\varepsilon}_{\mathrm{C}}^{(j-1)}\end{aligned} \tag{76}$$

for any $j \in \{1, \ldots, m\}$. Inductively this implies that

$$\tilde{\varepsilon}_{\mathrm{A}}^{(j)} = 2^j \varepsilon_{\mathrm{A}}^{(m)} = 2^{-m+j}\varepsilon_{\mathrm{A}} \qquad \text{and} \qquad \tilde{\varepsilon}_{\mathrm{C}}^{(j)} = 2^{-j}\varepsilon_{\mathrm{C}}^{(m)} = 2^{m-j}\varepsilon_{\mathrm{C}} \qquad \text{for all } j \in \{1, \ldots, m\}$$

where we used (74). Thus we can apply Lemma 5.6 since by assumptions on ε_{A} and ε_{C} we have $\tilde{\varepsilon}_{\mathrm{A}}^{(j)} < 1/4$ and $\tilde{\varepsilon}_{\mathrm{C}}^{(j)} < 1/4$ for all $j \in \{1, \ldots, m\}$.

We claim that

$$\begin{aligned}\tilde{x}^{(j)} &= x^{(m-j)} \\ \tilde{z}^{(j)} &= z^{(m-j)}\end{aligned} \qquad \text{for all} \qquad j \in \{0, \ldots, m\} \; . \tag{77}$$

The claim is trivially true for $j = 0$ by definition (see Eq. (75)). Inductively, we have for $j \in \{1, \ldots, m\}$

$$\begin{aligned}\tilde{x}^{(j)} &= 2\tilde{x}^{(j-1)} + \lfloor \tilde{z}^{(j-1)} \rceil_0 && \text{by definition (Eq. (76))} \\ &= 2x^{(m-(j-1))} + \lfloor z^{(m-(j-1))} \rceil_0 && \text{by the induction hypothesis} \\ &= (x^{(m-(j-1)-1)} - \lfloor x^{(m-(j-1)-1)} \rceil_0) + \lfloor 2z^{(m-(j-1)-1)} + \lfloor x^{(m-(j-1)-1)} \rceil_0 \rceil_0 && \text{by definition (Eq. (73))} \\ &= (x^{(m-(j-1)-1)} - \lfloor x^{(m-(j-1)-1)} \rceil_0) + \lfloor x^{(m-(j-1)-1)} \rceil_0 \\ &= x^{(m-j)} \; .\end{aligned}$$

In the penultimate step, we used that $z^{(m-j)} \in \mathbb{N}_0(\varepsilon_{\mathrm{C}}^{(m-j)}) \subseteq \mathbb{N}_0(\varepsilon)$ for some $\varepsilon < 1/4$. Similarly, we have

$$\begin{aligned}\tilde{z}^{(j)} &= (\tilde{z}^{(j-1)} - \lfloor \tilde{z}^{(j-1)} \rceil_0)/2 && \text{by definition (Eq. (76))} \\ &= (z^{(m-(j-1))} - \lfloor z^{(m-(j-1))} \rceil_0)/2 && \text{by the induction hypothesis} \\ &= (2z^{(m-(j-1)-1)} + \lfloor x^{(m-(j-1)-1)} \rceil_0 - \lfloor 2z^{(m-(j-1)-1)} + \lfloor x^{(m-(j-1)-1)} \rceil_0 \rceil_0)/2 && \text{by definition (Eq. (73))} \\ &= (2z^{(m-j)} + \lfloor x^{(m-j)} \rceil_0 - \lfloor x^{(m-j)} \rceil_0)/2 \\ &= z^{(m-j)} \; .\end{aligned}$$

In the penultimate step, we used that $z^{(m-(j-1)-1)} = z^{(m-j)} \in \mathbb{N}_0(\varepsilon_{\mathrm{C}}^{(m-j)}) \subseteq \mathbb{N}_0(\varepsilon)$ for some $\varepsilon < 1/4$.

Next, we show that

$$\tilde{y}^{(j)} = \left(\prod_{i=1}^{j}(1/\beta_i)^{\lfloor x\rceil_{m-i}}\right)\cdot y^{(m)} \qquad \text{for all} \qquad j\in\{0,\ldots,m\}\ . \tag{78}$$

Eq. (78) is obtained inductively by using $\tilde{y}^{(j)} = (1/\beta_j)^{\lfloor x\rceil_{m-j}}\cdot\tilde{y}^{(j-1)}$, which can be shown as follows: We have

$$\begin{aligned}
\tilde{y}^{(j)} &= (1/\beta_j)^{\lfloor \tilde{z}^{(j-1)}\rceil_0}\cdot\tilde{y}^{(j-1)} && \text{by definition (Eq. (76))}\\
&= (1/\beta_j)^{\lfloor z^{(m-(j-1))}\rceil_0}\cdot\tilde{y}^{(j-1)} && \text{by (77)}\\
&= (1/\beta_j)^{\lfloor 2z^{(m-j)}+\lfloor x^{(m-j)}\rceil_0\rceil_0}\cdot\tilde{y}^{(j-1)}\\
&= (1/\beta_j)^{\lfloor x^{(m-j)}\rceil_0}\cdot\tilde{y}^{(j-1)} && (79)\\
&= (1/\beta_j)^{\lfloor x\rceil_{m-j}}\cdot\tilde{y}^{(j-1)} && (80)
\end{aligned}$$

where we obtained (79) from $z^{(m-j)}\in\mathbb{N}_0(\varepsilon_{\mathrm{C}}^{(m-j)})\subseteq\mathbb{N}_0(\varepsilon)$ for some $\varepsilon<1/4$ and Eq. (80) by

$$\lfloor x^{(j)}\rceil_0 = \lfloor x\rceil_j \qquad \text{for } j\in\{0,\ldots,m\}\ . \tag{81}$$

This claim can be seen as follows. We have

$$\begin{aligned}
\lfloor x^{(j)}\rceil_0 &= \lfloor (x^{(j-1)}-\lfloor x^{(j-1)}\rceil_0)/2\rceil_0 && \text{by definition (see (73))}\\
&= \lfloor x^{(j-1)}\rceil_1
\end{aligned}$$

where we used the identity $\lfloor (x-\lfloor x\rceil_0)/2\rceil_0 = \lfloor x\rceil_1$ for any $x\in\mathbb{R}$. We obtain (81) by induction.

In conclusion, we have shown the following action of the unitary $V(\vec{\beta},\vec{\alpha})$.

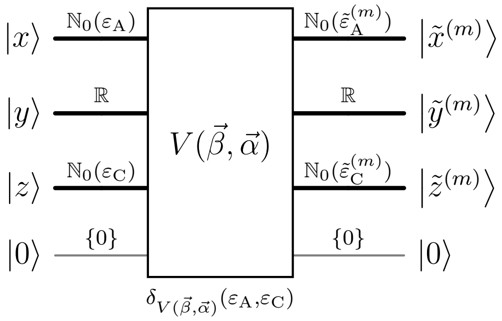

where

$$\begin{aligned}
\tilde{x}^{(m)} &= x^{(0)} = x\\
\tilde{y}^{(m)} &= \left(\textstyle\prod_{j=1}^{m}(1/\beta_j)^{\lfloor x\rceil_{m-j}}\right)\cdot y^{(m)} = g_{\vec{\beta}}(\lfloor x\rceil)\cdot f_{\vec{\alpha}}(\lfloor x\rceil)\cdot y \qquad \text{and} \qquad \begin{aligned}\tilde{\varepsilon}_{\mathrm{A}}^{(m)} &= \varepsilon_{\mathrm{A}}\\ \tilde{\varepsilon}_{\mathrm{C}}^{(m)} &= \varepsilon_{\mathrm{C}}\end{aligned}\ .\\
\tilde{z}^{(m)} &= z^{(0)} = z
\end{aligned}$$

It remains to determine the error bound $\delta_{V(\vec{\beta},\vec{\alpha})}(\varepsilon_{\mathrm{A}},\varepsilon_{\mathrm{C}})$. By Lemma 5.5 we have the error bound

$$\delta_{V^{\dagger}_{\alpha_j}}(\varepsilon_{\mathrm{A}}^{(j-1)},\varepsilon_{\mathrm{C}}^{(j-1)}) = 7(\varepsilon_{\mathrm{A}}^{(j-1)})^{1/4} + 7\cdot(2\varepsilon_{\mathrm{C}}^{(j-1)})^{1/4}\ ,$$

and by Lemma 5.6 we have

$$\delta_{V^{\dagger}_{\beta_j}}(\tilde{\varepsilon}_{\mathrm{A}}^{(j-1)},\tilde{\varepsilon}_{\mathrm{C}}^{(j-1)}) = 7(\tilde{\varepsilon}_{\mathrm{A}}^{(j-1)})^{1/4} + 7\cdot(2\tilde{\varepsilon}_{\mathrm{C}}^{(j-1)})^{1/4}\ .$$

We obtain an error bound $\delta_{V_{\alpha_m}\cdots V_{\alpha_1}}$ on the approximate action of the composition of unitaries $V_{\alpha_m}\cdots V_{\alpha_1}$ from Lemma 5.1, that is, we can use

$$\begin{aligned}
\delta_{V_{\alpha_m}\cdots V_{\alpha_1}} &= \sum_{j=1}^{m} \delta_{V_{\alpha_j}}(\varepsilon_{\mathrm{A}}^{(j-1)}, \varepsilon_{\mathrm{C}}^{(j-1)}) \\
&= \sum_{j=1}^{m} \Big(7(\varepsilon_{\mathrm{A}}^{(j-1)})^{1/4} + 7(2\varepsilon_{\mathrm{C}}^{(j-1)})^{1/4}\Big) \\
&= \sum_{j=1}^{m} \Big(7(2^{-(j-1)}\varepsilon_{\mathrm{A}})^{1/4} + 7(2\cdot 2^{j-1}\varepsilon_{\mathrm{C}})^{1/4}\Big) \\
&= 7\varepsilon_{\mathrm{A}}^{1/4}\sum_{j=0}^{m-1}\left(\frac{1}{2^{1/4}}\right)^{j} + 7(2\varepsilon_{\mathrm{C}})^{1/4}\sum_{j=0}^{m-1}\left(2^{1/4}\right)^{j} \\
&= 7\varepsilon_{\mathrm{A}}^{1/4}\frac{1-2^{-m/4}}{1-2^{-1/4}} + 7(2\varepsilon_{\mathrm{C}})^{1/4}\frac{1-2^{m/4}}{1-2^{1/4}} \\
&= 7\varepsilon_{\mathrm{A}}^{1/4}\frac{1-2^{-m/4}}{1-2^{-1/4}} + 7\varepsilon_{\mathrm{C}}^{1/4}\frac{2^{m/4}-1}{1-2^{-1/4}} \\
&\leq 44\cdot\varepsilon_{\mathrm{A}}^{1/4} + 44\cdot(2^{m}\varepsilon_{\mathrm{C}})^{1/4}\ . \qquad (82)
\end{aligned}$$

An error bound on the composition of unitaries $V^{\dagger}_{\beta_m}\cdots V^{\dagger}_{\beta_1}$ is obtained analogously as

$$\begin{aligned}
\delta_{V^{\dagger}_{\beta_m}\cdots V^{\dagger}_{\beta_1}} &= \sum_{j=1}^{m} \delta_{V^{\dagger}_{\beta_j}}(\varepsilon_{\mathrm{A}}^{(m+j)}, \varepsilon_{\mathrm{C}}^{(m+j)}) \\
&= \sum_{j=1}^{m} \Big(7(2\cdot\varepsilon_{\mathrm{A}}^{(m+j)})^{1/4} + 7(\varepsilon_{\mathrm{C}}^{(m+j)})^{1/4}\Big) \\
&= \sum_{j=1}^{m} \Big(7(2\cdot 2^{-m+(j-1)}\varepsilon_{\mathrm{A}})^{1/4} + 7(2^{m-(j-1)}\varepsilon_{\mathrm{C}})^{1/4}\Big) \\
&= 7\cdot 2^{-(m-1)/4}\varepsilon_{\mathrm{A}}^{1/4}\sum_{j=0}^{m-1}(2^{1/4})^{j} + 7\cdot 2^{m/4}\varepsilon_{\mathrm{C}}^{1/4}\sum_{j=0}^{m-1}\left(\frac{1}{2^{1/4}}\right)^{j} \\
&= 7\cdot 2^{-(m-1)/4}\varepsilon_{\mathrm{A}}^{1/4}\frac{1-2^{m/4}}{1-2^{1/4}} + 7\cdot 2^{m/4}\varepsilon_{\mathrm{C}}^{1/4}\frac{1-2^{-m/4}}{1-2^{-1/4}} \\
&= 7\cdot\varepsilon_{\mathrm{A}}^{1/4}\frac{1-2^{-m/4}}{1-2^{-1/4}} + 7\cdot\varepsilon_{\mathrm{C}}^{1/4}\frac{2^{m/4}-1}{1-2^{-1/4}} \\
&\leq 44\cdot\varepsilon_{A}^{1/4} + 44\cdot(2^{m}\varepsilon_{\mathrm{C}})^{1/4}\ . \qquad (83)
\end{aligned}$$

Combining (82) and (83) yields

$$\delta_{V_{\alpha_m}\cdots V_{\alpha_1}} + \delta_{V^{\dagger}_{\beta_m}\cdots V^{\dagger}_{\beta_1}} \leq 88\cdot\varepsilon_{\mathrm{A}}^{1/4} + 88\cdot(2^{m}\varepsilon_{\mathrm{C}})^{1/4}\ .$$

We set $\delta_{V(\vec{\beta},\vec{\alpha})}(\varepsilon_{\mathrm{A}}, \varepsilon_{\mathrm{C}}) := 88\cdot\varepsilon_{\mathrm{A}}^{1/4} + 88\cdot(2^{m}\varepsilon_{\mathrm{C}})^{1/4}$. This completes the proof. □

We show the properties of the unitary $V_{a,N,m}$ defined in Figure 2 of the main text, whose

definition we recall here:

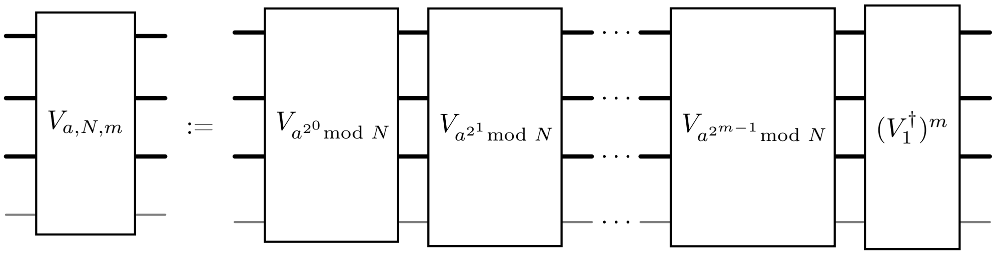

(84)

where we used the mth power of the unitary $(V_1^\dagger)^m$ to indicate the m-fold application of the unitary $V_1^\dagger$.

Lemma 5.8. *Let $\varepsilon_\mathrm{A} \in (0, 1/4)$ and let $\varepsilon_\mathrm{C} \in (0, 2^{-(m+2)})$. Consider the unitary $V_{a,N,m}$ described by the circuit in Figure 2 of the main text. Then*

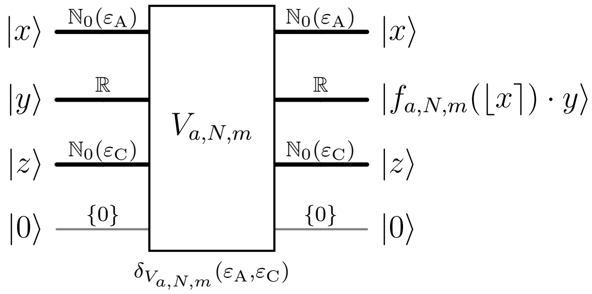

where the function $f_{a,N,m}(\lfloor x \rceil) = \prod_{i=0}^{m-1} \left(a^{2^i} \bmod N\right)^{\lfloor x \rceil_i}$ *is the pseudomodular power and where* $\delta_{V_{a,N,m}}(\varepsilon_\mathrm{A}, \varepsilon_\mathrm{C}) = 88 \cdot \varepsilon_\mathrm{A}^{1/4} + 88 \cdot (2^m \varepsilon_\mathrm{C})^{1/4}$.

Proof. We apply Lemma 5.7 with

$$\vec{\alpha} = (\alpha_1, \ldots, \alpha_m) \qquad \text{where } \alpha_j = a^{2^{j-1}} \bmod N\,,$$
$$\vec{\beta} = (\beta_1, \ldots, \beta_m) \qquad \text{where } \beta_j = 1$$

for all $j \in \{1, \ldots m\}$. The claim follows since $V_{a,N,m} = V(\vec{\beta}, \vec{\alpha})$ by definition and since

$$f_{\vec{\alpha}}(\lfloor x \rceil) = \prod_{i=1}^{m} \left(a^{2^{i-1}} \bmod N\right)^{\lfloor x \rceil_{i-1}} = f_{a,N,m}(\lfloor x \rceil) \qquad \text{and}$$
$$g_{\vec{\beta}}(\lfloor x \rceil) = 1\ .$$

□

We will need the following statement about the inverse $V_{a,N,m}^\dagger$.

Lemma 5.9. *Let $\varepsilon_\mathrm{A} \in (0, 1/4)$ and let $\varepsilon_\mathrm{C} \in (0, 2^{-(m+2)})$. Consider the unitary $V_{a,N,m}$ described by the circuit in Figure 2 of the main text. Then the action of its inverse $V_{a,N,m}^\dagger$ is*

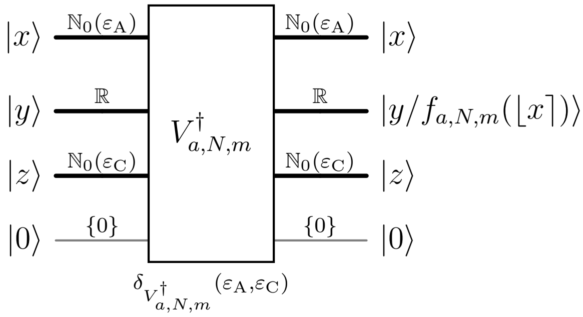

,

where $\delta_{V^\dagger_{a,N,m}}(\varepsilon_{\rm A},\varepsilon_{\rm C}) = \delta_{V_{a,N,m}}(\varepsilon_{\rm A},\varepsilon_{\rm C}) = 88\cdot\varepsilon_{\rm A}^{1/4} + 88\cdot(2^m\varepsilon_{\rm C})^{1/4}$

Proof. By taking the adjoint of $V_{a,N,m}$ in Eq. (84) we have

$$V^\dagger_{a,N,m} = \left((V_1^\dagger)^m \cdot V_{a^{2^{m-1}} \bmod N}\cdots V_{a^{2^0} \bmod N}\right)^\dagger = V^\dagger_{a^{2^0} \bmod N}\cdots V^\dagger_{a^{2^{m-1}} \bmod N}\cdot (V_1)^m \ .$$

Thus we can apply Lemma 5.7 with

$$\begin{aligned}\vec\alpha &= (\alpha_1,\ldots,\alpha_m) \qquad \text{where } \alpha_j = 1\ ,\\ \vec\beta &= (\beta_1,\ldots,\beta_m) \qquad \text{where } \beta_j = a^{2^{m-j}} \mod N\end{aligned}$$

for all $j\in\{0,1\}$. The claim follows since

$$\begin{aligned} f_{\vec\alpha}(\lfloor x\rceil) &= 1 \qquad \text{and}\\ g_{\vec\beta}(\lfloor x\rceil) &= \prod_{i=1}^m 1/\left(a^{2^{m-i}} \mod N\right)^{\lfloor x\rceil_{m-i}} = 1/f_{a,N,m}(\lfloor x\rceil)\ .\end{aligned}$$

□

We now analyse the gate $U_{a,N,m}$ implementing the controlled translation by a pseudomodular power (see Figure 2 of the main text) which we recall here.

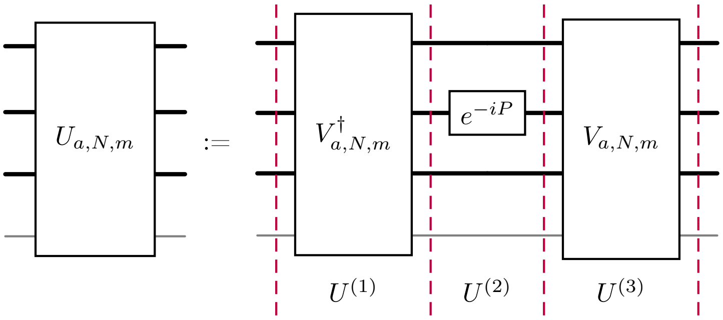

This circuit is composed of the unitaries

$$U_{a,N,m} = U^{(3)}U^{(2)}U^{(1)} \qquad \text{where} \qquad \begin{aligned} U^{(1)} &= V^\dagger_{a,N,m}\\ U^{(2)} &= I_{\rm A}\otimes e^{-iP_{\rm B}}\otimes I_{\rm C}\otimes I_{\rm Q}\ .\\ U^{(3)} &= V_{a,N,m}\end{aligned}$$

The approximate actions of these unitaries on inputs which are close to integers on modes A and C are given in Supplementary Figure 9.

Lemma 5.10. *Let* $\varepsilon_{\rm A}\in(0,1/4)$ *and* $\varepsilon_{\rm C}\in(0,2^{-(m+2)})$. *Consider the unitary* $U_{a,N,m}$ *described by the circuit in Figure 2 of the main text. Then*

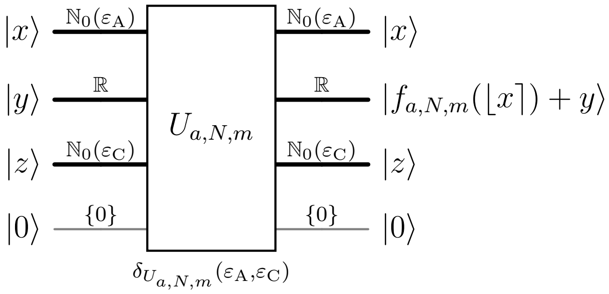

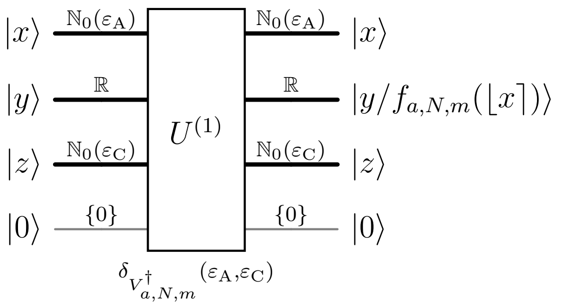

(a) The action of the unitary $U^{(1)}$ is given by Lemma 5.9. Here $\delta_{V^\dagger_{a,N,m}}(\varepsilon_\mathrm{A},\varepsilon_\mathrm{C}) = \delta_{V_{a,N,m}}(\varepsilon_\mathrm{A},\varepsilon_\mathrm{C}) = 88\cdot\varepsilon_\mathrm{A}^{1/4} + 88\cdot(2^m\varepsilon_\mathrm{C})^{1/4}$.

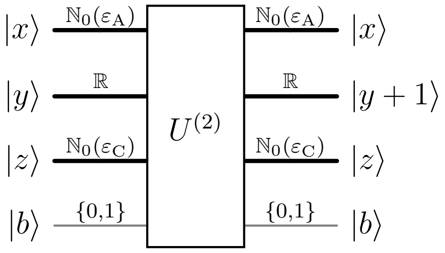

(b) The action of the unitary $U^{(2)} = I_\mathrm{A}\otimes e^{-iP_\mathrm{B}}\otimes I_\mathrm{C}\otimes I_\mathrm{Q}$. This unitary is implemented exactly.

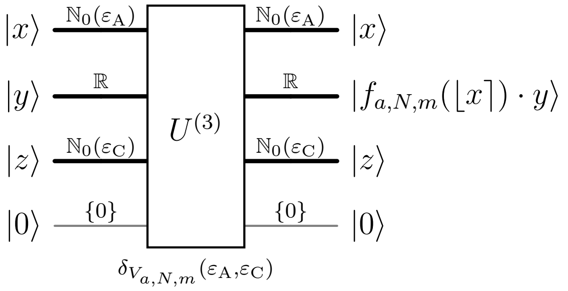

(c) The action of the unitary $U^{(3)}$ given by Lemma 5.8 with $\delta_{V_{a,N,m}}(\varepsilon_\mathrm{A},\varepsilon_\mathrm{C}) = 88\cdot\varepsilon_\mathrm{A}^{1/4} + 88\cdot(2^m\varepsilon_\mathrm{C})^{1/4}$.

Supplementary Figure 9. The approximate actions of the unitaries $U^{(1)}$, $U^{(2)}$ and $U^{(3)}$ constituting the unitary $U_{a,N,m} = U^{(3)}U^{(2)}U^{(1)}$.

where $\delta_{U_{a,N,m}} = 176\cdot\varepsilon_\mathrm{A}^{1/4} + 176\cdot(2^m\varepsilon_\mathrm{C})^{1/4}$.

Proof. We use Lemma 5.1 to obtain the approximate action of the combined unitaries $U^{(2)}U^{(1)}$ and $U^{(3)}U^{(2)}U^{(1)}$ from the decomposition of unitary $U_{a,N,m}$. Those are displayed in Supplementary Figure 10. The claim follows from the action of the unitary $U^{(3)}U^{(2)}U^{(1)} = U_{a,N,m}$ shown in Supplementary Figure 10b and by the identity $\delta_{V^\dagger_{a,N,m}}(\varepsilon_\mathrm{A},\varepsilon_\mathrm{C}) = \delta_{V_{a,N,m}}(\varepsilon_\mathrm{A},\varepsilon_\mathrm{C}) = 88\cdot\varepsilon_\mathrm{A}^{1/4} + 88\cdot(2^m\varepsilon_\mathrm{C})^{1/4}$, see Supplementary Figure 9a. □

5.5 Implications for the algorithm

Finally, we can give a bound on the distance $\left\|\Psi^{(1)} - \Psi^{(2)}\right\|_1$, the last remaining component of the proof of Proposition 2 (see Section 4).

Lemma 5.11. *Let $N\in\mathbb{N}$ be an n-bit integer. Let $(m, R, \kappa_\mathrm{A}, \Delta_\mathrm{A}, \kappa_\mathrm{B}, \Delta_\mathrm{B}, \Delta_\mathrm{C})$ be as specified in Supplementary Table 1 and let $\varepsilon_\mathrm{A} = \sqrt{\Delta_\mathrm{A}}$, $\varepsilon_\mathrm{B} = \sqrt{\Delta_\mathrm{B}}$ and $\varepsilon_\mathrm{C} = \sqrt{\Delta_\mathrm{C}}$. Consider the two states*

$$\left|\Psi^{(1)}\right\rangle = U_{a,N,m}\big(c_1\cdot\Pi_{[-1/2,2R-1/2]}e^{-iRP}\left|\mathsf{GKP}^{\varepsilon_\mathrm{A}}_{\kappa_\mathrm{A},\Delta_\mathrm{A}}\right\rangle\otimes M_N\left|\mathsf{GKP}^{\varepsilon_\mathrm{B}}_{\kappa_\mathrm{B},\Delta_\mathrm{B}}\right\rangle\otimes\left|\Psi^{\varepsilon_\mathrm{C}}_{\Delta_\mathrm{C}}\right\rangle\otimes|0\rangle\big)$$

and

$$\left|\Psi^{(2)}\right\rangle = c_2\sum_{z=0}^{2R-1}\eta_{\kappa_\mathrm{A}}(z-R)\left|\chi^{\varepsilon_\mathrm{A}}_{\Delta_\mathrm{A}}(z)\right\rangle\otimes e^{-if_{a,N,m}(z)P}M_N\left|\mathsf{GKP}^{\varepsilon_\mathrm{B}}_{\kappa_\mathrm{B},\Delta_\mathrm{B}}\right\rangle\otimes\left|\Psi^{\varepsilon_\mathrm{C}}_{\Delta_\mathrm{C}}\right\rangle\otimes|0\rangle\,.$$

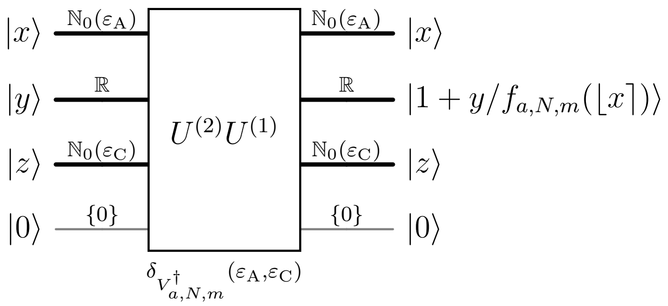

(a) The action of the composed unitary $U^{(2)}U^{(1)}$ given by composing the action of $U^{(1)}$ from Supplementary Figure 9a with the action of $U^{(2)}$ from Supplementary Figure 9b.

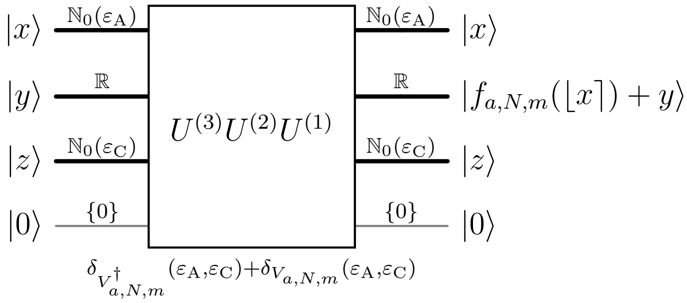

(b) The action of the composed unitary $U^{(3)}U^{(2)}U^{(1)} = U_{a,N,m}$ obtained by composing the action of the unitary $U^{(2)}U^{(1)}$ from Supplementary Figure 10a with the action of the unitary $U^{(3)}$ in Supplementary Figure 9c.

Supplementary Figure 10. Combined action of the unitaries $U^{(1)}$, $U^{(2)}$ and $U^{(3)}$ from Supplementary Figure 9 composing $U_{a,N,m}$ obtained by repeated application of Lemma 5.1.

Then

$$\left\| \Psi^{(1)} - \Psi^{(2)} \right\|_1 \leq 352 \cdot 2^{-2n} =: \varepsilon^{(2)} \ .$$

Proof. Since $\varepsilon_{\rm A} < 1/2$ can write

$$\left|\Psi^{(1)}\right\rangle = U_{a,N,m} \left(c_1 \sum_{z=0}^{2R-1} \eta_{\kappa_{\rm A}}(z-R) \left|\chi^{\varepsilon_{\rm A}}_{\Delta_{\rm A}}(z)\right\rangle \otimes M_N \left|\mathsf{GKP}^{\varepsilon_{\rm B}}_{\kappa_{\rm B},\Delta_{\rm B}}\right\rangle \otimes \left|\Psi^{\varepsilon_{\rm C}}_{\Delta_{\rm C}}\right\rangle \otimes |0\rangle \right) \ .$$

In Lemma 5.10 we showed that the approximate action of the unitary $U_{a,N,m}$ is

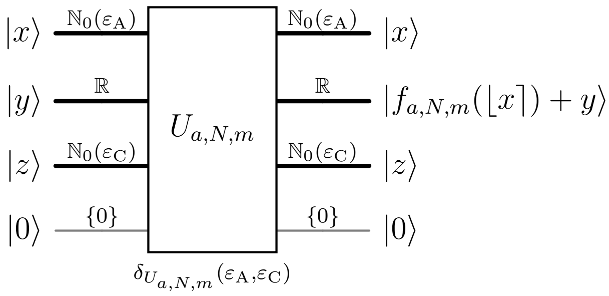

Since $|\lambda + y\rangle = e^{-i\lambda P}|y\rangle$ for any $\lambda, y \in \mathbb{R}$ we have that $|f_{a,N,m}(\lfloor x \rceil) + y\rangle = e^{-i f_{a,N,m}(\lfloor x \rceil)P}|y\rangle$.

Therefore by Definition 5.1, we have

$$\begin{aligned}\left\|\Psi^{(1)} - \Psi^{(2)}\right\|_1 &\leq \delta_{U_{a,N,m}(\varepsilon_{\mathrm{A}},\varepsilon_{\mathrm{C}})} \\ &= 176 \cdot \varepsilon_{\mathrm{A}}^{1/4} + 176 \cdot (2^m \varepsilon_{\mathrm{C}})^{1/4} \\ &= 176 \cdot \varepsilon_{\mathrm{A}}^{1/4} + 176 \cdot (2^m (2^{-m} \varepsilon_{\mathrm{A}}))^{1/4} \\ &= 352 \cdot \varepsilon_{\mathrm{A}}^{1/4} \\ &= 352 \cdot 2^{-2n} \ ,\end{aligned}$$

where we noted that $\varepsilon_{\mathrm{C}} = 2^{-m}\varepsilon_{\mathrm{A}}$ and $\varepsilon_{\mathrm{A}} = \sqrt{\Delta_{\mathrm{A}}}$ and used the parameters $(m, R, \kappa_{\mathrm{A}}, \Delta_{\mathrm{A}}, \kappa_{\mathrm{B}}, \Delta_{\mathrm{B}}, \Delta_{\mathrm{C}})$ given in Supplementary Table 1. □

6 Proof of Proposition 3: Distribution of measurement outcomes

The goal of this section is to prove Proposition 3. This proposition centers around the distribution p_{Φ_a} on $\mathbb{R}$ obtained by performing a homodyne P-quadrature measurement on mode A of the state $\Phi_a \in L^2(\mathbb{R})^{\otimes 3} \otimes \mathbb{C}^2$ defined in Eq. (25) of Proposition 2 which we repeat here

$$|\Phi_a\rangle = c_{\Phi_a} \sum_{z\in\mathbb{Z}} \eta_{\kappa_\mathrm{A}}(z-R) \, |\chi_{\Delta_\mathrm{A}}(z)\rangle \otimes e^{-i(a^z \mod N)P_\mathrm{B}} M_N \left|\mathsf{GKP}^{\varepsilon_\mathrm{B}}_{\kappa_\mathrm{B},\Delta_\mathrm{B}}\right\rangle \otimes \left|\Psi^{\varepsilon_\mathrm{C}}_{\Delta_\mathrm{C}}\right\rangle \otimes |0\rangle \tag{85}$$

where $c_{\Phi_a} > 0$ is a normalization constant. Recall that by Proposition 2, this state is close to the output state of the circuit $\mathcal{Q}_{a,N}$. In particular, the following property of p_{Φ_a} immediately implies an analogous property for the actual output distribution of the circuit (see Eq. (26)).

The property of interest — established by Proposition 3 — is that the family of distributions $\{p_{\Phi_a}\}_{a\in\mathbb{Z}_N^*}$ is suitable (for factoring) in the sense of Definition 2.1, i.e., it satisfies

$$\min_{d\in\mathbb{Z}_{r(a)}} \int_{\Gamma_d(a)} p_{\Phi_a}(w)dw \geq \Omega(1)\cdot \frac{1}{r(a)} \qquad \text{for all} \qquad a\in\mathbb{Z}_N^* \ ,$$

where $r(a)$ is the multiplicative order of a in $\mathbb{Z}_N^*$. For convenience, let us restate this claim.

Proposition 3 (Distribution of measurement outcomes)**.** *Let $N \geq 2$. For $a \in \mathbb{Z}_N^*$ let $\Phi_a \in L^2(\mathbb{R})^{\otimes 3}\otimes\mathbb{C}^2$ be the state defined by Eq.* (25) *of Proposition 2, with the parameters* $(m, R, \kappa_\mathrm{A}, \Delta_\mathrm{A}, \kappa_\mathrm{B}, \Delta_\mathrm{B}, \Delta_\mathrm{C})$ *specified by Supplementary Table 1 and with $\varepsilon_\mathrm{B} = \sqrt{\Delta_\mathrm{B}}$ and $\varepsilon_\mathrm{C} = \sqrt{\Delta_\mathrm{C}}$. Let $p_{\Phi_a} : \mathbb{R} \to [0,\infty)$ be the probability density function of the distribution of outcomes when performing a P-quadrature measurement on mode* A *of Φ_a. Then the family of distributions $\{p_{\Phi_a}\}_{a\in\mathbb{Z}_N^*}$ is suitable.*

In order to compute the output distribution when measuring the momentum (i.e., P-quadrature) of mode A of the state Φ_a, we proceed as follows: In Section 6.1 we compute an expression of Φ_a in momentum space (i.e., after a Fourier transform on $L^2(\mathbb{R})$). We will then analyze the Fourier-transform using Poisson's formula, and establish lower bounds on its modulus in certain intervals of interest, i.e., $\Gamma_d(a)$ for $d \in \mathbb{Z}_{r(a)}$, see Section 6.2. Finally, in Section 6.3, we conclude the proof by analyzing the probability density function p_{Φ_a} given by Born's rule when measuring the P-quadrature of mode A.

6.1 The Fourier transform of Φ_a

In this section, we compute the Fourier transform of Φ_a given in (85). For $a \in \mathbb{Z}_N^*$, it will be convenient to define the set

$$\mathrm{Rem}_{a,N} := \{a^z \mod N \,|\, z\in\mathbb{Z}\} \ . \tag{86}$$

That is, $\mathrm{Rem}_{a,N} \subseteq \mathbb{Z}_N^*$ is the subgroup generated by the element a. Observe that the set $\mathrm{Rem}_{a,N}$ has cardinality $|\mathrm{Rem}_{a,N}| = r(a)$ because $a \in \mathbb{Z}_N^*$ has order $r(a)$.

By straightforward computation, we show the following:

Lemma 6.1. *We have*

$$|\Phi_a\rangle = \sum_{k\in Rem_{a,N}} |\Theta_k\rangle \otimes e^{-ikP_\mathrm{B}} M_N \left|\mathsf{GKP}^{\varepsilon_\mathrm{B}}_{\kappa_\mathrm{B},\Delta_\mathrm{B}}\right\rangle \otimes \left|\Psi^{\varepsilon_\mathrm{C}}_{\Delta_\mathrm{C}}\right\rangle \otimes |0\rangle \tag{87}$$

where for each $k \in Rem_{a,N}$, the function Θ_k has the Fourier transform

$$\widehat{\Theta}_k(w) = c_{\Phi_a} \sum_{\substack{z\in\mathbb{Z}\\ a^z \mod N=k}} \eta_{\kappa_\mathrm{A}}(z-R)\widehat{\Psi}_{\Delta_\mathrm{A}}(w)e^{2\pi izw} \ . \tag{88}$$

Furthermore, the reduced density operator $\rho_{\mathrm{A}} = \mathrm{tr}_{\mathrm{BCQ}} |\Phi\rangle\langle\Phi|$ *after tracing out modes* B,C *and the qubit* Q *is equal to*

$$\rho_{\mathrm{A}} = \sum_{k \in Rem_{a,N}} |\Theta_k\rangle\langle\Theta_k| \ . \tag{89}$$

We note that the vectors $\{|\Theta_k\rangle\}_{k\in\mathrm{Rem}_{a,N}}$ in this expression are not normalized.

Proof. It is straightforward to verify that Φ_a in (85) can be written in the form of Eq. (87) with

$$|\Theta_k\rangle = c_{\Phi_a} \sum_{\substack{z\in\mathbb{Z} \\ a^z \mod N=k}} \eta_{\kappa_{\mathrm{A}}}(z-R)\, |\chi_{\Delta_{\mathrm{A}}}(z)\rangle \qquad \text{for} \qquad k \in \mathrm{Rem}_{a,N} \ .$$

By definition of the Fourier transform and $\chi_{\Delta_{\mathrm{A}}}(\cdot)$, we have

$$\widehat{\Theta}_k(w) = c_{\Phi_a} \sum_{\substack{z\in\mathbb{Z} \\ a^z \mod N=k}} \eta_{\kappa_{\mathrm{A}}}(z-R) \int \Psi_{\Delta_{\mathrm{A}}}(x-z) e^{2\pi i x w} dx \ .$$

Expression (88) follows from the fact that

$$\int f(x-z) e^{2\pi i x w} dx = \widehat{f}(w) e^{2\pi i z w}$$

for any $f \in L^1(\mathbb{R}) \cap L^2(\mathbb{R})$.

The claim (89) about the reduced density operator ρ_{A} follows immediately from the pairwise orthogonality of the states $\left\{ e^{-ikP_{\mathrm{B}}} M_N \left| \mathsf{GKP}^{\varepsilon_{\mathrm{B}}}_{\kappa_{\mathrm{B}},\Delta_{\mathrm{B}}} \right\rangle \right\}_{k\in\mathrm{Rem}_{a,N}}$ which we argue in the following. For $k \neq k' \in \mathrm{Rem}_{a,N}$ we have

$$\begin{aligned}
\langle e^{-ikP} M_N \mathsf{GKP}^{\varepsilon_{\mathrm{B}}}_{\kappa_{\mathrm{B}},\Delta_{\mathrm{B}}}, e^{-ik'P} M_N \mathsf{GKP}^{\varepsilon_{\mathrm{B}}}_{\kappa_{\mathrm{B}},\Delta_{\mathrm{B}}} \rangle &= \langle \mathsf{GKP}^{\varepsilon_{\mathrm{B}}}_{\kappa_{\mathrm{B}},\Delta_{\mathrm{B}}}, M_N^\dagger e^{i(k'-k')P} M_N \mathsf{GKP}^{\varepsilon_{\mathrm{B}}}_{\kappa_{\mathrm{B}},\Delta_{\mathrm{B}}} \rangle \\
&= \langle \mathsf{GKP}^{\varepsilon_{\mathrm{B}}}_{\kappa_{\mathrm{B}},\Delta_{\mathrm{B}}}, M_N^\dagger e^{i(k-k')P/N} M_N \mathsf{GKP}^{\varepsilon_{\mathrm{B}}}_{\kappa_{\mathrm{B}},\Delta_{\mathrm{B}}} \rangle \ .
\end{aligned}$$

It is easy to check that for $k \neq k'$ the real number $d = (k-k')/N$ is at distance at least $1/N$ from the closest integer. Since $\varepsilon_{\mathrm{B}} = 2^{-9n^2} < 2^{-(n+1)} \leq 1/(2N)$ by the choice of parameters (see Supplementary Table 1), it follows from Lemma 7.6 that

$$\langle \mathsf{GKP}^{\varepsilon_{\mathrm{B}}}_{\kappa_{\mathrm{B}},\Delta_{\mathrm{B}}}, M_N^\dagger e^{idP} M_N \mathsf{GKP}^{\varepsilon_{\mathrm{B}}}_{\kappa_{\mathrm{B}},\Delta_{\mathrm{B}}} \rangle = 0 \ . \tag{90}$$

□

For later reference, we observe the following: Because of the pairwise orthogonality of the states $\left\{ e^{-ikP_{\mathrm{B}}} M_N \left| \mathsf{GKP}^{\varepsilon_{\mathrm{B}}}_{\kappa_{\mathrm{B}},\Delta_{\mathrm{B}}} \right\rangle \right\}_{k\in\mathrm{Rem}_{a,N}}$, Lemma 6.1 implies that the probability density function $p_{\Phi_a} : \mathbb{R} \to \mathbb{R}$ of obtaining an outcome $w \in \mathbb{R}$ when measuring the P-quadrature of mode A in the state Φ_a is given by

$$p_{\Phi_a}(w) = \sum_{k\in\mathrm{Rem}_{a,N}} \left| \widehat{\Theta}_k(w) \right|^2 \ . \tag{91}$$

6.2 Bounds on the Fourier transform

We show that $|\widehat{\Theta}_k(w)|$ constituting the probability density function in Eq. (91) can be rewritten as follows.

Lemma 6.2. *Let $N \in \mathbb{N}$, $a \in \mathbb{Z}_N^*$, and $k \in Rem_{a,N}$ be arbitrary. Let $r = r(a)$ be the order of a in $\mathbb{Z}_N^*$. Then $\widehat{\Theta}_k(w)$ (see Eq. (88)) satisfies*

$$\left|\widehat{\Theta}_k(w)\right| = |c_{\Phi_a}|\frac{|\widehat{\Psi}_{\Delta_A}(w)|}{r}\left|\sum_{z\in\mathbb{Z}} e^{2\pi i(R-\mathsf{ind}_a(k))z/r}\widehat{\eta}_{\kappa_A}(w+z/r)\right| \qquad \text{for any } w \in \mathbb{R} \ , \tag{92}$$

where for $k \in Rem_{a,N}$ we denote the index of k to the base a modulo N by $\mathsf{ind}_a(k) \in \mathbb{Z}_r$, i.e., $\mathsf{ind}_a(k) \in \mathbb{Z}_r$ is the smallest number in $\{0, \ldots, r-1\}$ satisfying $a^{\mathsf{ind}_a(k)} \equiv k \pmod N$.

Proof. Let $k \in \mathrm{Rem}_{a,N}$. Let $\mathcal{L}$ be the lattice defined as

$$\mathcal{L} = \{z \in \mathbb{Z} \mid a^z \mod N = 1\} \ .$$

Since r is the order of a in $\mathbb{Z}_N^*$, we have $\mathcal{L} = r\mathbb{Z}$. The corresponding dual lattice $\mathcal{L}^* = \{y \in \mathbb{R} : y \cdot z \in \mathbb{Z} \text{ for all } z \in \mathcal{L}\}$ is $\mathcal{L}^* = \frac{1}{r}\mathbb{Z}$, which has lattice determinant (see [9])

$$\det \mathcal{L}^* = \frac{1}{r} \ . \tag{93}$$

We also have that the translated lattice $\mathsf{ind}_a(k) + \mathcal{L}$ satisfies

$$\mathsf{ind}_a(k) + \mathcal{L} = \{z \in \mathbb{Z} \mid a^z \mod N = k\} \tag{94}$$

by definition of $\mathsf{ind}_a(k)$.

Using (94), we can rewrite $\widehat{\Theta}_k$ given in Eq. (88) as

$$\begin{aligned}\widehat{\Theta}_k(w) &= c_{\Phi_a}\sum_{z\in\mathsf{ind}_a(k)+\mathcal{L}} \eta_{\kappa_A}(z-R)\widehat{\Psi}_{\Delta_A}(w)e^{2\pi i z w}\\ &= c_{\Phi_a}e^{2\pi i w\cdot\mathsf{ind}_a(k)}\widehat{\Psi}_{\Delta_A}(w)\sum_{y\in\mathcal{L}} f(y) \ ,\end{aligned} \tag{95}$$

i.e., as a sum over lattice points $y \in \mathcal{L}$. Here we introduced the function

$$\begin{aligned}f(y) &= \eta_{\kappa_A}(y+\mathsf{ind}_a(k)-R)e^{2\pi i y w}\\ &= \eta_{\kappa_A}(y+J)e^{2\pi i y w} \qquad \text{where} \qquad J := \mathsf{ind}_a(k) - R \ .\end{aligned} \tag{96}$$

The Fourier transform of f is

$$\begin{aligned}\widehat{f}(v) &= \int f(y)e^{2\pi i y v}dy\\ &= \int \eta_{\kappa_A}(y+J)e^{2\pi i y w}e^{2\pi i y v}dy\\ &= \int \eta_{\kappa_A}(z)e^{2\pi i(z-J)w}e^{2\pi i(z-J)v}dz\\ &= e^{-2\pi i J(v+w)}\int \eta_{\kappa_A}(z)e^{2\pi i z(v+w)}dz\\ &= e^{-2\pi i J(v+w)}\widehat{\eta}_{\kappa_A}(v+w) \ .\end{aligned}$$

where we used the variable substitution $z = y + J$ to obtain the third line. With the Poisson summation formula (see e.g., [9])

$$\sum_{y\in\mathcal{L}} f(y) = (\det \mathcal{L}^*)\sum_{v\in\mathcal{L}^*}\widehat{f}(v)$$

and (95) in combination with the definition of dual lattice $\mathcal{L}^* = \frac{1}{r}\mathbb{Z}$ and its determinant from with (93), we thus obtain the expression

$$\begin{aligned}
\widehat{\Theta}_k(w) &= c_{\Phi_a} e^{2\pi i w\cdot \mathsf{ind}_a(k)} \widehat{\Psi}_{\Delta_{\mathrm{A}}}(w) \frac{1}{r} \sum_{y\in\frac{1}{r}\mathbb{Z}} e^{-2\pi i J(y+w)} \widehat{\eta}_{\kappa_{\mathrm{A}}}(y+w)\\
&= c_{\Phi_a} e^{2\pi i w\cdot \mathsf{ind}_a(k)} \widehat{\Psi}_{\Delta_{\mathrm{A}}}(w) \frac{1}{r} \sum_{z\in\mathbb{Z}} e^{-2\pi i J(z/r+w)} \widehat{\eta}_{\kappa_{\mathrm{A}}}(z/r+w)\\
&= c_{\Phi_a} e^{2\pi i (w\cdot \mathsf{ind}_a(k) - Jw)} \widehat{\Psi}_{\Delta_{\mathrm{A}}}(w) \frac{1}{r} \sum_{z\in\mathbb{Z}} e^{-2\pi i Jz/r} \widehat{\eta}_{\kappa_{\mathrm{A}}}(z/r+w)
\end{aligned}$$

where we substituted $y = z/r$ to obtain the second line. With the definition of J in (96), it follows that

$$\left|\widehat{\Theta}_k(w)\right| = |c_{\Phi_a}| \cdot \frac{|\widehat{\Psi}_{\Delta_{\mathrm{A}}}(w)|}{r} \cdot \left|\sum_{z\in\mathbb{Z}} e^{2\pi i (R-\mathsf{ind}_a(k))z/r} \widehat{\eta}_{\kappa_{\mathrm{A}}}(w+z/r)\right| .$$

□

Using the expression (92) from Lemma 6.2, we can lower bound $|\widehat{\Theta}_k(w)|$ for very specific values of w as follows.

Lemma 6.3. *Let $N \geq 2$, $a \in \mathbb{Z}_N^*$ and $k \in Rem_{a,N}$ be arbitrary. Let $r = r(a)$ be the order of a in $\mathbb{Z}_N^*$. Let the parameter κ_A be defined by Supplementary Table 1. Then*

$$\left|\widehat{\Theta}_k(\omega + m/r)\right| \geq \frac{e^{-\pi^2/2}}{\sqrt{\kappa_{\mathrm{A}}}} \cdot |c_{\Phi_a}| \cdot \frac{|\widehat{\Psi}_{\Delta_{\mathrm{A}}}(\omega+m/r)|}{r} \qquad \text{for all} \quad \omega \in \left[\frac{\kappa_{\mathrm{A}}}{4}, \frac{\kappa_{\mathrm{A}}}{2}\right] \ \text{ and } m \in \mathbb{Z} .$$

Proof. Let $\varphi \in \mathbb{R}$ be arbitrary. Then we have for $\omega \in \left[\frac{\kappa_{\mathrm{A}}}{4}, \frac{\kappa_{\mathrm{A}}}{2}\right]$ and $m \in \mathbb{Z}$

$$\begin{aligned}
\sum_{z\in\mathbb{Z}} e^{i\varphi z} \widehat{\eta}_{\kappa_{\mathrm{A}}}((\omega+m/r)+z/r) &= e^{-im\varphi} \sum_{y\in\mathbb{Z}} e^{i\varphi y} \widehat{\eta}_{\kappa_{\mathrm{A}}}(\omega+y/r)\\
&= e^{-im\varphi} \frac{\sqrt{2}\pi^{1/4}}{\sqrt{\kappa_{\mathrm{A}}}} \sum_{y\in\mathbb{Z}} e^{i\varphi y} e^{-2\pi^2(\omega+y/r)^2/\kappa_{\mathrm{A}}^2} \qquad (97)
\end{aligned}$$

where we used the variable substitution $y = m + z$ in the first identity, and the fact that the Fourier transform of $\eta_{\kappa_{\mathrm{A}}}$ is $\widehat{\eta}_{\kappa_{\mathrm{A}}}(w) = \frac{\sqrt{2}\pi^{1/4}}{\sqrt{\kappa_{\mathrm{A}}}} e^{-2\pi^2 w^2/\kappa_{\mathrm{A}}^2}$ in the second identity. Defining

$$\begin{aligned}
\xi &= e^{i\varphi} e^{-4\pi^2\omega/(\kappa_{\mathrm{A}}^2 r)}\\
b &= e^{-2\pi^2/(\kappa_{\mathrm{A}}^2 r^2)} ,
\end{aligned}$$

Eq. (97) can be rewritten as

$$\sum_{z\in\mathbb{Z}} e^{i\varphi z} \widehat{\eta}_{\kappa_{\mathrm{A}}}((\omega+m/r)+z/r) = e^{-im\varphi} \frac{\sqrt{2}\pi^{1/4} e^{-2\pi^2\omega^2/\kappa_{\mathrm{A}}^2}}{\sqrt{\kappa_{\mathrm{A}}}} \sum_{y\in\mathbb{Z}} \xi^y b^{y^2} . \qquad (98)$$

For our choice of parameters we have $\frac{\omega}{\kappa_{\mathrm{A}}^2 r} \geq \frac{1}{4\kappa_{\mathrm{A}} r} \geq \frac{N}{4}$ (because $\frac{1}{\kappa_{\mathrm{A}}} > N^2$ and $r \leq N$). It follows that $\max\{|\xi|, |\xi|^{-1}\} = |\xi^{-1}| \geq e^{\pi^2 N} \geq \sqrt{2}+1$. Furthermore, we have $b < 1$ by definition. This means that we can apply Lemma 8.5, which gives a lower bound on a sum of the form $\sum_{y\in\mathbb{Z}} \xi^y b^{y^2}$. Applied to (98) this gives

$$\begin{aligned}
\left|\sum_{z\in\mathbb{Z}} e^{i\varphi z} \widehat{\eta}_{\kappa_{\mathrm{A}}}((\omega+m/r)+z/r)\right| &\geq \frac{\sqrt{2}\pi^{1/4} e^{-2\pi^2\omega^2/\kappa_{\mathrm{A}}^2}}{\sqrt{\kappa_{\mathrm{A}}}} \left(1 - 2e^{-2\pi^2/(\kappa_{\mathrm{A}}^2 r^2)} \cdot e^{4\pi^2\omega/(\kappa_{\mathrm{A}}^2 r)}\right)\\
&\geq \frac{\sqrt{2}\pi^{1/4} e^{-2\pi^2\omega^2/\kappa_{\mathrm{A}}^2}}{\sqrt{\kappa_{\mathrm{A}}}} \left(1 - 2e^{-\pi^2 N^2}\right) , \qquad (99)
\end{aligned}$$

where we used that

$$\begin{aligned} e^{-2\pi^2/(\kappa_{\rm A}^2 r^2)} e^{4\pi^2\omega/(\kappa_{\rm A}^2 r)} &\leq e^{-2\pi^2/(\kappa_{\rm A}^2 r^2)} e^{2\pi^2/(\kappa_{\rm A} r)} \qquad \text{since } \omega \leq \kappa_{\rm A}/2 \\ &= e^{-2\pi^2 x(x-1)} \qquad \text{with } x = 1/(\kappa_{\rm A} r) \geq N \\ &\leq e^{-2\pi^2 N(N-1)} \qquad \text{as } x \mapsto x(x-1) \text{ increases monotonically} \\ &\leq e^{-\pi^2 N^2} \qquad \text{for } N \geq 2 \ . \end{aligned}$$

With (98) and (99), as well as the choice $\varphi = 2\pi(R - \mathsf{ind}_a(k))/r$, we conclude that for $w = \omega + m/r$, we have

$$\left| \sum_{z \in \mathbb{Z}} e^{2\pi i (R - \mathsf{ind}_a(k)) z/r} \widehat{\eta}_{\kappa_{\rm A}}(w + z/r) \right| = \frac{\sqrt{2}\pi^{1/4} e^{-2\pi^2\omega^2/\kappa_{\rm A}^2}}{\sqrt{\kappa_{\rm A}}} \left(1 - 2e^{-\pi^2 N^2}\right) \geq \frac{e^{-2\pi^2\omega^2/\kappa_{\rm A}^2}}{\sqrt{\kappa_{\rm A}}}$$

where we used that $\sqrt{2}\pi^{1/4}(1 - 2e^{-\pi^2 N^2}) \geq \sqrt{2}\pi^{1/4}(1 - 2e^{-\pi^2}) \geq 1$. Because of Eq. (92), this means that

$$\left|\widehat{\Theta}_k(w)\right| \geq \frac{e^{-2\pi^2\omega^2/\kappa_{\rm A}^2}}{\sqrt{\kappa_{\rm A}}} \cdot |c_{\Phi_a}| \cdot \frac{|\widehat{\Psi}_{\Delta_{\rm A}}(w)|}{r} \qquad \text{for} \qquad w = \omega + m/r \ .$$

The claim follows from the fact that the function $\omega \mapsto e^{-2\pi^2\omega^2/\kappa_{\rm A}^2}$ is monotonically decreasing for $\omega \geq 0$ combined with the assumption $\omega \in [\frac{\kappa_A}{4}, \frac{\kappa_A}{2}]$. □

6.3 Bounds on the probability distribution of outcomes

Recall the definition (see Eq. (24)) of the set

$$\Gamma_d(a) := \bigcup_{j \in \mathbb{Z}} \left[j + \frac{d}{r} - \frac{1}{2q}, j + \frac{d}{r} + \frac{1}{2q} \right] \ , \tag{100}$$

for $a \in \mathbb{Z}_N^*$ and $d \in \mathbb{Z}_r$, where $r = r(a)$ is the order of a and $q = \min\{2^k \mid k \in \mathbb{N}, 2^k > N^2\}$ is the smallest power of 2 greater than N^2. Here we use Lemma 6.3 to establish a lower bound on the probability density function p_{Φ_a} integrated over the set $\Gamma_d(a)$. We show that this quantity is lower bounded by a constant multiple of $1/r$ independent of $a \in \mathbb{Z}_N^*$ and $d \in \mathbb{Z}_r$. This will completes the proof of Proposition 3.

Lemma 6.4. *Let $(m, R, \kappa_{\rm A}, \Delta_{\rm A}, \kappa_{\rm B}, \Delta_{\rm B}, \Delta_{\rm C})$ be defined by Supplementary Table 1. Let $\varepsilon_{\rm B} = \sqrt{\Delta_{\rm B}}$ and $\varepsilon_{\rm C} = \sqrt{\Delta_{\rm C}}$. Let $N \geq 4$ and $a \in \mathbb{Z}_N^*$ be arbitrary. Let $r = r(a)$ be the order of a. Then*

$$\int_{\Gamma_d(a)} p_{\Phi_a}(w) dw \geq \frac{e^{-\pi^2}}{16} \cdot \frac{1}{r} \qquad \textit{for any} \qquad d \in \mathbb{Z}_r \ . \tag{101}$$

Proof. Let $a \in \mathbb{Z}_N^*$ be arbitrary. Fix an arbitrary $d \in \mathbb{Z}_r$, and an arbitrary $k \in \mathrm{Rem}_{a,N}$ (cf. Eq. (86)). To lower bound each integral in (101) we will replace the set $\Gamma_d(a)$ by a suitable subset. Since $q > 1$, the intervals in the definition (100) of $\Gamma_d(a)$ are pairwise disjoint, i.e., we have

$$\left[j + \frac{d}{r} - \frac{1}{2q}, j + \frac{d}{r} + \frac{1}{2q} \right] \cap \left[j' + \frac{d}{r} - \frac{1}{2q}, j' + \frac{d}{r} + \frac{1}{2q} \right] = \emptyset \qquad \text{for} \qquad j \neq j' \ .$$

Since $\left[\frac{\kappa_{\rm A}}{4}, \frac{\kappa_{\rm A}}{2}\right] \subseteq \left[-\frac{1}{2q}, \frac{1}{2q}\right]$ as $\kappa_{\rm A} \leq q^{-1}$ by the choice of parameters from Supplementary Table 1, we have

$$\bigcup_{j \in \mathbb{Z}} \left[j + \frac{d}{r} + \frac{\kappa_{\rm A}}{4}, j + \frac{d}{r} + \frac{\kappa_{\rm A}}{2} \right] \subseteq \Gamma_d(a) \ .$$

It follows that

$$
\begin{aligned}
\int_{\Gamma_d(a)} |\widehat{\Theta}_k(w)|^2 dw &\geq \sum_{j\in\mathbb{Z}} \int_{j+\frac{d}{r}+\frac{\kappa_A}{4}}^{j+\frac{d}{r}+\frac{\kappa_A}{2}} \left|\widehat{\Theta}_k(w)\right|^2 dw \\
&= \sum_{j\in\mathbb{Z}} \int_{\frac{\kappa_A}{4}}^{\frac{\kappa_A}{2}} \left|\widehat{\Theta}_k\left(j+\frac{d}{r}+\omega\right)\right|^2 d\omega \qquad \text{by substitution } w = j+\frac{d}{r}+\omega \\
&\geq \frac{e^{-\pi^2}}{\kappa_A}\cdot\frac{|c_{\Phi_a}|^2}{r^2}\sum_{j\in\mathbb{Z}} \int_{\frac{\kappa_A}{4}}^{\frac{\kappa_A}{2}} \left|\widehat{\Psi}_{\Delta_A}\left(j+\frac{d}{r}+\omega\right)\right|^2 d\omega \qquad \text{by Lemma 6.3} \\
&\geq \frac{e^{-\pi^2}}{\kappa_A}\cdot\frac{|c_{\Phi_a}|^2}{r^2}\sum_{j\in\mathbb{Z}} \frac{\kappa_A}{4} \min_{\omega\in[\frac{\kappa_A}{4},\frac{\kappa_A}{2}]} \left|\widehat{\Psi}_{\Delta_A}\left(j+\frac{d}{r}+\omega\right)\right|^2 \\
&= \frac{e^{-\pi^2}}{4}\cdot\frac{|c_{\Phi_a}|^2}{r^2}\sum_{j\in\mathbb{Z}} \min_{\omega\in[\frac{\kappa_A}{4},\frac{\kappa_A}{2}]} \left|\widehat{\Psi}_{\Delta_A}\left(j+\frac{d}{r}+\omega\right)\right|^2 . \qquad (102)
\end{aligned}
$$

We have

$$
\begin{aligned}
\left|j+\frac{d}{r}+\omega\right| &\leq |j| + \frac{d}{r} + |\omega| \\
&\leq |j| + \frac{d}{r} + \frac{\kappa_A}{2} \qquad \text{for} \qquad \omega\in[\kappa_A/4, \kappa_A/2] \\
&\leq |j| + \frac{r-1}{r} + \frac{\kappa_A}{2} \qquad \text{because } d\in\mathbb{Z}_r \\
&\leq |j| + 1 , \qquad (103)
\end{aligned}
$$

where in the last step, we used that $\kappa_A/2 \leq 1/r$ (by the choice of the parameters in Supplementary Table 1). Because $\widehat{\Psi}_{\Delta_A}(w) = \sqrt{2}\pi^{1/4}\sqrt{\Delta_A}e^{-2\pi^2 w^2\Delta_A^2}$ is symmetric and monotonically decreasing for $w \geq 0$, it follows from (103) that for any $j\in\mathbb{Z}$ and $d\in\mathbb{Z}_r$ we have

$$
\begin{aligned}
\min_{\omega\in[\frac{\kappa_A}{4},\frac{\kappa_A}{2}]} \left|\widehat{\Psi}_{\Delta_A}\left(j+\frac{d}{r}+\omega\right)\right|^2 &\geq |\widehat{\Psi}_{\Delta_A}(|j|+1)|^2 \\
&= 2\sqrt{\pi}\Delta_A e^{-4\pi^2\Delta_A^2(|j|+1)^2} . \qquad (104)
\end{aligned}
$$

Inserting (104) into (102) we conclude that

$$
\begin{aligned}
\int_{\Gamma_d(a)} |\widehat{\Theta}_k(w)|^2 dw &\geq \frac{e^{-\pi^2}\sqrt{\pi}}{2}\cdot\frac{\Delta_A\cdot|c_{\Phi_a}|^2}{r^2}\sum_{j\in\mathbb{Z}} e^{-4\pi^2\Delta_A^2(|j|+1)^2} \\
&= \frac{e^{-\pi^2}\sqrt{\pi}}{2}\cdot\frac{\Delta_A\cdot|c_{\Phi_a}|^2}{r^2}\sum_{j\in\mathbb{Z}} e^{-c(|j|+1)^2} \qquad \text{where} \qquad c = 4\pi^2\Delta_A^2 \\
&\geq \frac{e^{-\pi^2}}{16}\cdot\frac{|c_{\Phi_a}|^2}{r^2} ,
\end{aligned}
$$

where we used Lemma 8.1 to obtain the last inequality. The latter can be applied because of our choice of parameters, which implies that $c < \pi/16$. Using the lower bound on $|c_{\Phi_a}|^2$ given in Lemma 6.5 below we obtain

$$
\int_{\Gamma_d(a)} |\widehat{\Theta}_k(w)|^2 dw \geq \frac{e^{-\pi^2}}{64}\cdot\frac{1}{r^2} \qquad \text{for all} \qquad d\in\mathbb{Z}_r \text{ and } k\in\mathrm{Rem}_{a,N} . \qquad (105)
$$

Combining Eq. (91), i.e., the expression

$$
p_{\Phi_a}(w) = \sum_{k\in\mathrm{Rem}_{a,N}} \left|\widehat{\Theta}_k(w)\right|^2 .
$$

with (105) implies (by the linearity of the integral) that

$$\int_{\Gamma_d(a)} p_{\Phi_a}(w)dw = \sum_{k\in \mathrm{Rem}_{a,N}} \int_{\Gamma_d(a)} \left|\widehat{\Theta}_k(w)\right|^2 dw$$
$$\geq \frac{e^{-\pi^2}}{64}\cdot\frac{1}{r}$$

where we used that the set $\mathrm{Rem}_{a,N}$ consists of $|\mathrm{Rem}_{a,N}| = r$ elements. This is the claim. □

It remains to establish a bound on the normalization constant c_{Φ_a} in the definition (85) of the state Φ_a.

Lemma 6.5. *Let* $(m, R, \kappa_{\mathrm{A}}, \Delta_{\mathrm{A}}, \kappa_{\mathrm{B}}, \Delta_{\mathrm{B}}, \Delta_{\mathrm{C}})$ *be as specified in Supplementary Table 1. Let* $\varepsilon_{\mathrm{B}} = \sqrt{\Delta_{\mathrm{B}}}$ *and* $\varepsilon_{\mathrm{C}} = \sqrt{\Delta_{\mathrm{C}}}$*. Then*

$$|c_{\Phi_a}|^2 \geq 1/4\ .$$

Proof. By definition of the state Φ_a (cf. (85)) and the pairwise orthogonality of the family of states $\left\{e^{-ikP_{\mathrm{B}}}M_N\left|\mathsf{GKP}^{\varepsilon_{\mathrm{B}}}_{\kappa_{\mathrm{B}},\Delta_{\mathrm{B}}}\right\rangle\right\}_{k\in\mathrm{Rem}_{a,N}}$ (see (90)) we have

$$|c_{\Phi_a}|^{-2} = \sum_{y,z\in\mathbb{Z}} \eta_{\kappa_{\mathrm{A}}}(y-R)\eta_{\kappa_{\mathrm{A}}}(z-R)\langle\chi_{\Delta_{\mathrm{A}}}(y),\chi_{\Delta_{\mathrm{A}}}(z)\rangle\cdot\delta_{a^y \bmod N, a^z \bmod N}\ . \tag{106}$$

Since

$$\eta_{\kappa_{\mathrm{A}}}(y-R)\eta_{\kappa_{\mathrm{A}}}(z-R)\langle\chi_{\Delta_{\mathrm{A}}}(y),\chi_{\Delta_{\mathrm{A}}}(z)\rangle \geq 0 \qquad \text{for all} \qquad y,z\in\mathbb{Z}\ ,$$

we can omit the term $\delta_{a^y \bmod N, a^z \bmod N}$ in Eq. (106) to get the upper bound

$$|c_{\Phi_a}|^{-2} \leq \sum_{y,z\in\mathbb{Z}} \eta_{\kappa_{\mathrm{A}}}(y-R)\eta_{\kappa_{\mathrm{A}}}(z-R)\langle\chi_{\Delta_{\mathrm{A}}}(y),\chi_{\Delta_{\mathrm{A}}}(z)\rangle\ .$$

Recalling that R is an integer, we can use a substitution to obtain

$$\begin{aligned}|c_{\Phi_a}|^{-2} &\leq \sum_{y,z\in\mathbb{Z}} \eta_{\kappa_{\mathrm{A}}}(y)\eta_{\kappa_{\mathrm{A}}}(z)\langle\chi_{\Delta_{\kappa_{\mathrm{A}}}}(y+R),\chi_{\Delta_{\kappa_{\mathrm{A}}}}(z+R)\rangle\\ &= \sum_{y,z\in\mathbb{Z}} \eta_{\kappa_{\mathrm{A}}}(y)\eta_{\kappa_{\mathrm{A}}}(z)\langle\chi_{\Delta_{\kappa_{\mathrm{A}}}}(y),\chi_{\Delta_{\kappa_{\mathrm{A}}}}(z)\rangle\end{aligned} \tag{107}$$

where we used that $\langle\chi_{\Delta_{\mathrm{A}}}(y+R),\chi_{\Delta_{\mathrm{A}}}(z+R)\rangle = \langle e^{-iRP}\chi_{\Delta_{\mathrm{A}}}(y), e^{-iRP}\chi_{\Delta_{\mathrm{A}}}(z)\rangle = \langle\chi_{\Delta_{\mathrm{A}}}(y),\chi_{\Delta_{\mathrm{A}}}(z)\rangle$ because e^{-iRP} is unitary. Comparing (107) with the definition of the approximate GKP state $|\mathsf{GKP}_{\kappa,\Delta}\rangle$ (cf. (21)), we obtain

$$|c_{\Phi_a}|^{-2} \leq C^{-2}_{\kappa_{\mathrm{A}},\Delta_{\mathrm{A}}}\ . \tag{108}$$

Our choice of parameters $(m, R, \kappa_{\mathrm{A}}, \Delta_{\mathrm{A}}, \kappa_{\mathrm{B}}, \Delta_{\mathrm{B}}, \Delta_{\mathrm{C}})$ (see Supplementary Table 1) guarantees that $\Delta_{\mathrm{A}} < 1/8$ and $\kappa_{\mathrm{A}} < 1/8$. Thus Lemma 7.2 can be applied, giving the bound

$$C^2_{\kappa_{\mathrm{A}},\Delta_{\mathrm{A}}} \geq 1/4\ . \tag{109}$$

Combining (108) and (109) gives the claim. □

7 Properties of approximate GKP states

In this section, we state some approximation results on Gaussians and their truncated versions, as well as analogous results for GKP-states. In separate work [2], we have used identical definitions, and established a few of the corresponding results. We refer to [2] for proofs in these cases.

First, recall that the for $\Delta > 0$, we denote by $\Psi_\Delta(x) = \frac{1}{(\pi\Delta^2)^{1/4}} e^{-x^2/(2\Delta^2)}$ a centered Gaussian (corresponding to a squeezed vacuum state $|\Psi_\Delta\rangle = M_\Delta |\mathrm{vac}\rangle$). The following lemma shows that it is well approximated by its truncated version $\Psi_\Delta^\varepsilon = \frac{\Pi_{[-\varepsilon,\varepsilon]}\Psi_\Delta}{\|\Pi_{[-\varepsilon,\varepsilon]}\Psi_\Delta\|}$ obtained by restricting its support to the interval $[-\varepsilon, \varepsilon]$, and renormalizing.

Lemma 7.1. *[2, Lemma A.2 and Corollary A.3] Let $\Delta > 0$ and $\varepsilon \in [\sqrt{\Delta}, 1/2)$. Then*

$$|\langle \Psi_\Delta, \Psi_\Delta^\varepsilon \rangle|^2 \geq 1 - 2\Delta$$

and

$$\|\Psi_\Delta - \Psi_\Delta^\varepsilon\|_1 \leq 3\sqrt{\Delta} \ .$$

Recall the definitions of the approximate GKP state (defined in (21))

$$|\mathsf{GKP}_{\kappa,\Delta}\rangle = C_{\kappa,\Delta} \sum_{z\in\mathbb{Z}} \eta_\kappa(z) |\chi_\Delta(z)\rangle \ ,$$

(with $\chi_\Delta(z) = \Psi_\Delta(x - z)$) as well as its truncated variant with $\varepsilon < 1/2$ (defined in (23))

$$|\mathsf{GKP}^\varepsilon_{\kappa,\Delta}\rangle = C_\kappa \sum_{z\in\mathbb{Z}} \eta_\kappa(z) |\chi^\varepsilon_\Delta(z)\rangle \ .$$

We use the following estimates on the normalization constants $C_{\kappa,\Delta}$ and C_κ.

Lemma 7.2. *[2, Lemma A.8 (specialized)] We have*

$$C^2_{\kappa,\Delta} \geq 1/4 \qquad \textit{for} \qquad \kappa, \Delta \in (0, 1/8) \tag{110}$$

and

$$\frac{C^2_{\kappa,\Delta}}{C^2_\kappa} \geq 1 - 7\Delta \qquad \textit{for} \qquad \kappa, \Delta \in (0, 1/4) \ . \tag{111}$$

Proof. By [2, Lemma A.8], we have

$$C^{-2}_{\kappa,\Delta} \leq 1 + \frac{\kappa}{\sqrt{\pi}} + 2(\sqrt{2\pi} + \kappa)\Delta \qquad \text{for } \kappa, \Delta > 0 \ ,$$

hence

$$C^2_{\kappa,\Delta} \geq 1 - \frac{\kappa}{\sqrt{\pi}} - 2(\sqrt{2\pi} + \kappa)\Delta \qquad \text{for } \kappa, \Delta > 0$$

by the inequality $1/(1 + x) \geq 1 - x$ for all $x > 0$. The claim (110) follows.

Furthermore, [2, Lemma A.8] shows that

$$\frac{C^2_{\kappa,\Delta}}{C^2_\kappa} \geq 1 - \frac{2(\sqrt{2\pi} + \kappa)}{1 - \kappa/\sqrt{\pi}}\Delta \qquad \text{for } \kappa > 0 \ ,$$

which implies the claim (111) for $\kappa < 1/8$. □

The fact that the states $\mathsf{GKP}_{\kappa,\Delta}$ and its truncated version $\mathsf{GKP}^\varepsilon_{\kappa,\Delta}$ are close (for suitably chosen parameters) is expressed by the following statement.

Lemma 7.3. *[2, Lemma A.9 (specialized)] Let* $\kappa \in (0, 1/4)$, $\Delta > 0$ *and* $\varepsilon \in [\sqrt{\Delta}, 1/2)$. *Then*

$$\left|\left\langle \mathsf{GKP}_{\kappa,\Delta}, \mathsf{GKP}^{\varepsilon}_{\kappa,\Delta}\right\rangle\right|^2 \geq 1 - 9\Delta$$

Proof. [2, Lemma A.9] shows that

$$\left|\left\langle \mathsf{GKP}_{\kappa,\Delta}, \mathsf{GKP}^{\varepsilon}_{\kappa,\Delta}\right\rangle\right|^2 \geq 1 - 7\Delta - 2e^{-(\varepsilon/\Delta)^2} \qquad \text{for } \kappa \in (0, 1/4),\ \Delta > 0 \text{ and } \varepsilon \in (0, 1/2) \tag{112}$$

The inequality $e^{-x} \leq x^{-1}$ for $x > 0$ together with the assumption on $\varepsilon \in [\sqrt{\Delta}, 1/2)$ imply the claim. □

We can show that the function $\mathsf{GKP}^{\varepsilon}_{\kappa,\Delta}$ has most of its support on the interval $[-r, r]$ for $r \gg 1/\kappa$, as expressed by the following lemma.

Lemma 7.4. *Let* $r \geq 4$ *and* $\kappa > 0$. *Let* $\varepsilon \in (0, 1/2)$. *Let* $\Pi_{[-r,r]}$ *be the projection onto the subspace of* $L^2(\mathbb{R})$ *of functions having support on* $[-r, r]$. *Then*

$$\left\|\Pi_{[-r,r]}\mathsf{GKP}^{\varepsilon}_{\kappa,\Delta}\right\|^2 \geq 1 - 2e^{-(\kappa r)^2} \ .$$

Proof. Recall the definition (112) of the state $\left|\mathsf{GKP}^{\varepsilon}_{\kappa,\Delta}\right\rangle$. Because of the pairwise orthogonality of the states $\{|\chi^{\varepsilon}_{\Delta}(z)\rangle\}_{z\in\mathbb{Z}}$, the normalization constant C_κ is given by

$$C_\kappa^{-2} = \sum_{z\in\mathbb{Z}} \eta_\kappa(z)^2 \ .$$

On the other hand, we have

$$\begin{aligned}
\left\|\Pi_{[-r,r]}\mathsf{GKP}^{\varepsilon}_{\kappa,\Delta}\right\|^2 &= C_\kappa^2 \sum_{z,z'\in\mathbb{Z}} \eta_\kappa(z)\eta_\kappa(z')\langle\chi^{\varepsilon}_{\Delta}(z), \Pi_{[-r,r]}\chi^{\varepsilon}_{\Delta}(z')\rangle \\
&\geq C_\kappa^2 \sum_{z=-t}^{t} \eta_\kappa(z)^2 \qquad \text{where } t := \lfloor r \rfloor - 1 \ .
\end{aligned}$$

where we used that each function $\chi^{\varepsilon}_{\Delta}(z)$ is supported on $[z-\varepsilon, z+\varepsilon]$ (implying that $\langle\chi^{\varepsilon}_{\Delta}(z), \Pi_{[-r,r]}\chi^{\varepsilon}_{\Delta}(z')\rangle = 0$ unless $z = z'$), and the fact that each such function is non-negative (which implies that $\langle\chi^{\varepsilon}_{\Delta}(z), \Pi_{[-r,r]}\chi^{\varepsilon}_{\Delta}(z)\rangle \leq \langle\chi^{\varepsilon}_{\Delta}(z), \chi^{\varepsilon}_{\Delta}(z)\rangle = \|\chi^{\varepsilon}_{\Delta}(z)\|^2 = 1$). It follows that

$$\begin{aligned}
\left\|\Pi_{[-r,r]}\mathsf{GKP}^{\varepsilon}_{\kappa,\Delta}\right\|^2 &\geq \frac{\sum_{z=-t}^{t}\eta_\kappa(z)^2}{\sum_{z\in\mathbb{Z}}\eta_\kappa(z)^2} \\
&= \frac{\sum_{z=-t}^{t}\rho_s(z)}{\sum_{z\in\mathbb{Z}}\rho_s(z)} \qquad \text{with} \qquad s = 1/\kappa \ ,
\end{aligned}$$

where we introduced the Gaussian function $\rho_s : \mathbb{R} \to \mathbb{R}$ for $s > 0$ as

$$\rho_s(z) = e^{-\pi z^2/s^2} \ .$$

A random variable X_s on $\mathbb{Z}$ sampled according to discrete Gaussian distribution with (width) parameter s (see e.g., [10]) is defined by

$$\Pr\left[X_s = x\right] = \frac{\rho_s(x)}{\rho_s(\mathbb{Z})} \qquad \text{where} \qquad \rho_s(\mathbb{Z}) = \sum_{z\in\mathbb{Z}} \rho_s(z) \ .$$

We thus have (by inserting $s = 1/\kappa$ and $t = \lfloor r \rceil - 1$)

$$
\begin{aligned}
\left\|\Pi_{[-r,r]}\mathsf{GKP}^{\varepsilon}_{\kappa,\Delta}\right\|^2 &\geq \Pr\left[|X_{1/\kappa}| \leq t\right] \\
&= \Pr\left[|X_{1/\kappa}| \leq \lfloor r \rceil - 1\right] \\
&= \Pr\left[|X_{1/\kappa}| \leq r - 1\right] \qquad (113) \\
&\geq \Pr\left[|X_{1/\kappa}| < r - 1\right] \\
&= 1 - \Pr\left[|X_{1/\kappa}| \geq r - 1\right] \ ,
\end{aligned}
$$

where we obtained (113) by the fact that the random variable $X_{1/\kappa}$ has support on $\mathbb{Z}$.

From Lemma 8.4 below we obtain

$$
\begin{aligned}
\left\|\Pi_{[-r,r]}\mathsf{GKP}^{\varepsilon}_{\kappa,\Delta}\right\|^2 &\geq 1 - 2e^{-\pi(\kappa(r-1))^2} \ . \\
&\geq 1 - 2e^{-\frac{\pi}{2}(\kappa r)^2/2} \qquad \text{whenever } r \geq 4 \\
&\geq 1 - 2e^{-(\kappa r)^2} \qquad \text{since } \pi/2 \geq 1.
\end{aligned}
$$

where we used that $(x-1)^2 \geq x^2/2$ for all $x \geq 4$ to obtain the inequality in the second step. This is the claim. □

Ideal GKP states were originally introduced because of there invariance under shifts. Here we show that approximate GKP states are approximately invariant under integer shifts that are small compared to $1/\kappa$. That is, we have:

Lemma 7.5 (Approximate shift-invariance of approximate GKP states)**.** *Let $\varepsilon \in (0, 1/2)$ and $\kappa, \Delta \in (0, 1/4)$. Then we have*

$$
\langle \mathsf{GKP}^{\varepsilon}_{\kappa,\Delta}, e^{idP}\mathsf{GKP}^{\varepsilon}_{\kappa,\Delta}\rangle \geq 1 - \frac{\kappa^2 d^2}{2} \qquad \textit{for any} \qquad d \in \mathbb{Z} \ .
$$

Proof. Using that $e^{idP}|\chi^{\varepsilon}_{\Delta}(z)\rangle = |\chi^{\varepsilon}_{\Delta}(z+d)\rangle$ we have, by the orthogonality of the states $\{|\chi^{\varepsilon}_{\Delta}(z)\rangle\}_{z\in\mathbb{Z}}$ and the assumption that d is an integer, that

$$
\begin{aligned}
\langle \mathsf{GKP}^{\varepsilon}_{\kappa,\Delta}, e^{idP}\mathsf{GKP}^{\varepsilon}_{\kappa,\Delta}\rangle &= C_\kappa^2 \sum_{z,z'\in\mathbb{Z}} \eta_\kappa(z)\eta_\kappa(z')\langle \chi^{\varepsilon}_{\Delta}(z), \chi^{\varepsilon}_{\Delta}(z'+d)\rangle \\
&= C_\kappa^2 \sum_{z\in\mathbb{Z}} \eta_\kappa(z)\eta_\kappa(z-d) \ ,
\end{aligned}
$$

where we used a variable substitution. Since $C_\kappa^{-2} = \sum_{z\in\mathbb{Z}} \eta_\kappa(z)^2$ it follows that

$$
\begin{aligned}
\langle \mathsf{GKP}^{\varepsilon}_{\kappa,\Delta}, e^{idP}\mathsf{GKP}^{\varepsilon}_{\kappa,\Delta}\rangle &= \frac{\sum_{z\in\mathbb{Z}} \eta_\kappa(z)\eta_\kappa(z-d)}{\sum_{z\in\mathbb{Z}} \eta_\kappa(z)^2} \\
&= \frac{\sum_{z\in\mathbb{Z}} e^{-\kappa^2 z^2/2} e^{-\kappa^2 (z-d)^2/2}}{\sum_{z\in\mathbb{Z}} (e^{-\kappa^2 z^2/2})^2} \qquad \text{by the definition of } \eta_\kappa(\cdot)\text{, see (22)}
\end{aligned}
$$

Hence the claim follows from Lemma 8.3 (with $c = \kappa^2/2$ and $\delta = d$). □

We also note that these states are orthogonal if shifted by a real number with a large distance from the set of integers compared to the parameter ε. That is, we have

Lemma 7.6 (Orthogonality of displaced GKP states)**.** *Let $\kappa, \Delta > 0$, $\varepsilon \in (0, 1/2)$ and $d \in \mathbb{R}$ be such that*

$$
|d - \lfloor d \rceil| > 2\varepsilon \ ,
$$

i.e., the distance of d to $\mathbb{Z}$ is at least ε. Then

$$
\langle \mathsf{GKP}^{\varepsilon}_{\kappa,\Delta}, e^{-idP}\mathsf{GKP}^{\varepsilon}_{\kappa,\Delta}\rangle = 0 \ .
$$

Proof. This follows immediately from the fact that $\mathsf{GKP}^{\varepsilon}_{\kappa,\Delta}$ is supported on $\mathbb{Z}(\varepsilon)$, whereas $e^{-idP}\mathsf{GKP}^{\varepsilon}_{\kappa,\Delta}$ has support on the translated set $(\mathbb{Z}+\{d\})(\varepsilon)$. If the deviation of real d from the closest integer satisfies $|d-\lfloor d\rceil|>2\varepsilon$, these sets are disjoint and the claim follows. $\square$

8 Combinatorial bounds

In this section, we state various bounds on sums of Gaussians. Some of these are derived by elementary means (e.g., using monotonicity of the exponential function and Gaussian integrals). Others are more involved and based on more sophisticated results about lattices, including Poisson summation formula, or the Jacobi triple product identity. We refer to [9–15] for more details.

8.1 Bounds on sums of Gaussians

Lemma 8.1. *Let $c \in (0, \pi/16)$. Then*

$$\sum_{z\in\mathbb{Z}} e^{-c(|z|+1)^2} \geq \frac{\sqrt{\pi}}{4\sqrt{c}} .$$

Proof. We show that for $c > 0$

$$\sum_{z\in\mathbb{Z}} e^{-c(|z|+1)^2} \geq \sqrt{\frac{\pi}{c}} - 3 . \tag{114}$$

The claim is a simple corollary for $c \in (0, \pi/16)$.

We have

$$\sum_{z\in\mathbb{Z}} e^{-c(|z|+1)^2} = \left(\sum_{z\in\mathbb{Z}} e^{-cz^2}\right) - 1 - e^{-c} \tag{115}$$

Since $x \mapsto e^{-cx^2}$ with $c > 0$ is monotonously decreasing for $x \geq 0$ we have

$$e^{-cz^2} \geq \int_z^{z+1} e^{-cx^2} dx \qquad \text{for any } z \geq 0 .$$

Since $x \mapsto e^{-cx^2}$ is even we have

$$\sum_{z\in\mathbb{Z}} e^{-cz^2} = 2\sum_{z\in\mathbb{N}_0} e^{-cz^2} - 1 \geq \int e^{-cx^2} dx - 1 \geq \sqrt{\frac{\pi}{c}} - 1 .$$

We obtain (114) by inserting this bound into (115) and by noting that $e^{-c} \leq 1$. □

Lemma 8.2. *For $s > 0$ and $x \in \mathbb{R}$ let $\rho_s(x) = e^{-\pi x^2/s^2}$ be a Gaussian function. Let*

$$\rho_s(\mathbb{Z} + t) := \sum_{z\in\mathbb{Z}} \rho_s(z + t) \qquad \textit{for } t \in \mathbb{R} .$$

Then for all $t \in \mathbb{R}$ the following holds:

(i) $\rho_s(\mathbb{Z} + t) \geq \rho_s(\mathbb{Z}) \cdot e^{-\pi t^2/s^2}$

(ii) $\rho_s(\mathbb{Z} + t) \leq \rho_s(\mathbb{Z})$.

Proof. We can prove Claim (i) as follows. We have

$$\begin{aligned}\rho_s(\mathbb{Z} + t) &= \sum_{z\in\mathbb{Z}} \rho_s(z + t) \\ &= \sum_{z\in\mathbb{Z}} e^{-\pi((z+t)/s)^2} \\ &= \sum_{z\in\mathbb{Z}} \frac{1}{2}\left(e^{-\pi((z+t)/s)^2} + e^{-\pi((t-z)/s)^2}\right) .\end{aligned} \tag{116}$$

But

$$\frac{1}{2}\left(e^{-\pi((z+t)/s)^2}+e^{-\pi((t-z)/s)^2}\right) = e^{-\pi(t/s)^2}e^{-\pi(z/s)^2}\cdot\frac{e^{-2\pi zt/s^2}+e^{2\pi zt/s^2}}{2}$$
$$= e^{-\pi(t/s)^2}e^{-\pi(z/s)^2}\cosh 2\pi zt/s^2$$
$$\geq e^{-\pi(t/s)^2}e^{-\pi(z/s)^2}\ , \tag{117}$$

where we used that $\frac{1}{2}(e^x+e^{-x})=\cosh x\geq 1$ for all $x\in\mathbb{R}$. Inserting (117) into (116) yields

$$\rho_s(\mathbb{Z}+t)\geq e^{-\pi t^2/s^2}\sum_{z\in\mathbb{Z}}e^{-\pi z^2/s^2}\ ,$$

which is the claim.

Claim (ii) follows as a direct consequence of the Poisson summation formula on $\mathbb{Z}$, see [9]. The latter states that for a Schwartz function $f:\mathbb{R}\to\mathbb{R}$, we have

$$\sum_{y\in\mathbb{Z}}f(y)=\sum_{v\in\mathbb{Z}}\widehat{f}(v)\qquad\text{where}\qquad\widehat{f}(k)=\int f(x)e^{2\pi ikx}dx\ .$$

Furthermore,

$$\sum_{y\in\mathbb{Z}}f(y+t)=\sum_{v\in\mathbb{Z}}\widehat{f}(v)e^{2\pi ivt}\ .$$

In particular, since the function $f=\rho_s$ has Fourier transform $\widehat{f}=s\rho_{1/s}$, we obtain by the Poisson summation formula that

$$\rho_s(\mathbb{Z}+t)=|\rho_s(\mathbb{Z}+t)|$$
$$=\left|\sum_{z\in\mathbb{Z}}\rho_s(z+t)\right|$$
$$=\left|s\sum_{v\in\mathbb{Z}}\rho_{1/s}(v)e^{2\pi ivt}\right|$$
$$\leq s\sum_{v\in\mathbb{Z}}\rho_{1/s}(v)\cdot|e^{2\pi ivt}| \tag{118}$$
$$=s\sum_{v\in\mathbb{Z}}\rho_{1/s}(v)\ ,$$

where we used the triangle inequality to obtain (118). Applying the Poisson summation formula a second time gives the claim. □

As an immediate application of part (i) of this lemma, we have the following bound on the ratio of two sums of Gaussians.

Lemma 8.3. *Let $c>0$ and $\delta>0$. Then*

$$\frac{\sum_{z\in\mathbb{Z}}e^{-cz^2}e^{-c(z-\delta)^2}}{\sum_{z\in\mathbb{Z}}\left(e^{-cz^2}\right)^2}\geq e^{-c\delta^2}\geq 1-c\delta^2\ .$$

Proof. We have

$$\frac{\sum_{z\in\mathbb{Z}}e^{-cz^2}e^{-c(z-\delta)^2}}{\sum_{z\in\mathbb{Z}}\left(e^{-cz^2}\right)^2}=e^{-c\delta^2/2}\cdot\frac{\sum_{z\in\mathbb{Z}}e^{-2c(z-\delta/2)^2}}{\sum_{z\in\mathbb{Z}}e^{-2cz^2}}$$
$$=e^{-c\delta^2/2}\cdot\frac{\rho_s(\mathbb{Z}-\delta/2)}{\rho_s(\mathbb{Z})}\qquad\text{with } s=\sqrt{\pi/(2c)}\ , \tag{119}$$

Application of Lemma 8.2 (i) gives the lower bound

$$\frac{\rho_s(\mathbb{Z}-\delta/2)}{\rho_s(\mathbb{Z})} \geq e^{-\pi(\delta/2)^2/s^2} = e^{-c\delta^2/2} \ .$$

The claim follows by inserting this bound to (119) and by the inequality $e^{-x} \geq 1-x$ for all $x \in \mathbb{R}$. □

8.2 A tail bound for discrete Gaussian distributions

We need a tail bound for discrete Gaussian distribution on $\mathbb{Z}$. This is a specializations of standard results of Refs. [12,13] that we reproduce here for completeness. We refer to [10,14,15] for more general results.

Lemma 8.4 ([13] Lemma 2.4 and [12] Lemma 1.5, specialized)**.** *Let $s > 0$. Set $\rho_s(z) = \exp\left(-\pi z^2/s^2\right)$. Define a random variable X_s on $\mathbb{Z}$ by*

$$\Pr\left[X_s = x\right] = \frac{\rho_s(x)}{\rho_s(\mathbb{Z})} \ ,$$

where $\rho_s(\mathbb{Z}) = \sum_{z\in\mathbb{Z}} \rho_s(z)$. Then we have

$$\Pr\left[|X_s| \geq r\right] \leq 2 \cdot e^{-\pi(r/s)^2}$$

for $r > 0$.

Proof. We can obtain the claim using the Chernoff tail bound, which states that for any random variable Y symmetric around zero and any $r > 0$, we have

$$\Pr\left[|Y| \geq r\right] \leq 2 \cdot \frac{\mathbb{E}e^{\lambda Y}}{e^{\lambda r}} \qquad \text{for all } \lambda > 0 \ . \tag{120}$$

Since ρ_s is an even function, X_s is symmetric around zero. We can use Lemma 8.2 (ii) to bound the moment generating function of X by

$$\begin{aligned}
\mathbb{E}e^{\lambda X_s} &= \frac{\sum_{z\in\mathbb{Z}} e^{\lambda z} e^{-\pi z^2/s^2}}{\rho_s(\mathbb{Z})} \\
&= \frac{e^{\lambda^2 s^2/(4\pi)} \sum_{z\in\mathbb{Z}} e^{-\pi(z-\frac{\lambda s^2}{2\pi})^2}}{\rho_s(\mathbb{Z})} \\
&= e^{\lambda^2 s^2/(4\pi)} \cdot \frac{\rho_s\left(\mathbb{Z}-\frac{\lambda s^2}{2\pi}\right)}{\rho_s(\mathbb{Z})} \\
&\leq e^{\lambda^2 s^2/(4\pi)} \ ,
\end{aligned} \tag{121}$$

where we used Lemma 8.2 (ii) to obtain the last line. Inserting (121) into (120) and setting $\lambda = \frac{2\pi r}{s^2} > 0$ yields the claim

$$\Pr\left[|X_s| \geq r\right] \leq 2 \cdot e^{-\pi(r/s)^2} \ .$$ □

8.3 A bound derived from Jacobi's triple product identity

We will also use the following lemma to lower bound sums of the form $\sum_{k\in\mathbb{Z}} \xi^k b^{k^2}$ when $\xi \in \mathbb{C}\backslash\{0\}$ and $b \in [0,1)$.

Lemma 8.5. *For $b \in [0,1)$ and $\xi \in \mathbb{C}\backslash\{0\}$ such that*

$$\max\{|\xi|, |\xi|^{-1}\} \geq \sqrt{2} + 1\,. \tag{122}$$

we have

$$\begin{aligned} \left|\sum_{k\in\mathbb{Z}} \xi^k b^{k^2}\right| &\geq \left(1 - 2b\max\{|\xi|, |\xi|^{-1}\}\right) \sum_{k=0}^{\infty} (b^4)^{k^2} \\ &\geq \left(1 - 2b\max\{|\xi|, |\xi|^{-1}\}\right)\,. \end{aligned}$$

Proof. Jacobi's triple product identity [11] states that for any $0 \leq b < 1$ and any $\xi \in \mathbb{C}\backslash\{0\}$, we have

$$\sum_{k\in\mathbb{Z}} \xi^k b^{k^2} = \prod_{k=1}^{\infty} \left(1 - b^{2k}\right)\left(1 + \xi b^{2k-1}\right)\left(1 + \xi^{-1} b^{2k-1}\right)\,. \tag{123}$$

We note that

$$\left(1 + \xi b^{2k-1}\right)\left(1 + \xi^{-1} b^{2k-1}\right) = 1 + (\xi + \xi^{-1}) b^{2k-1} + b^{2(2k-1)}$$

and thus

$$\left|\left(1 + \xi b^{2k-1}\right)\left(1 + \xi^{-1} b^{2k-1}\right)\right| \geq 1 - |\xi + \xi^{-1}| b^{2k-1} + b^{2(2k-1)}\,. \tag{124}$$

Let $r := \max\{|\xi|, |\xi|^{-1}\}$. It is easy to check that

$$|\xi + \xi^{-1}| \in [r - r^{-1}, r + r^{-1}]\,. \tag{125}$$

In particular, the assumption (122) implies that

$$|\xi + \xi^{-1}| \geq \left|r - r^{-1}\right| \geq 2\,. \tag{126}$$

Since $|\xi + \xi^{-1}| \geq 2$, there is some $x \geq 1$ such that $x + x^{-1} = |\xi + \xi^{-1}|$. Indeed, we can use the inverse

$$\begin{array}{rccc} f^{-1} & : [2,\infty) & \to & [1,\infty) \\ & z & \mapsto & f^{-1}(z) = \frac{1}{2}(z + \sqrt{z^2 - 4}) \end{array}$$

of the function

$$\begin{array}{rccc} f & : [1,\infty) & \to & [2,\infty) \\ & y & \mapsto & f(y) := y + y^{-1}\,, \end{array}$$

and set

$$x := f^{-1}(|\xi + \xi^{-1}|)\,.$$

Since f^{-1} is monotonically increasing in z, we have by (125) that

$$x \leq f^{-1}(r + r^{-1}) = r\,,$$

as well as

$$f^{-1}(r - r^{-1}) \leq x\,.$$

By a simple calculation, we also have

$$f^{-1}(r - r^{-1}) \geq f^{-1}(2) = 1\,,$$

where we used Eq. (126) and the fact that f^{-1} is monotonically increasing. In conclusion, we have shown that

$$1 \leq x \leq r\,. \tag{127}$$

By definition, we have

$$(-x) + (-x)^{-1} = -(x + x^{-1}) = -|\xi + \xi^{-1}|\,. \tag{128}$$

It follows that

$$\begin{aligned}
\left|\sum_{k\in\mathbb{Z}} \xi^k b^{k^2}\right| &= \prod_{k\geq 1}(1-b^{2k})\left|\left(1+\xi b^{2k-1}\right)\left(1+\xi^{-1}b^{2k-1}\right)\right| \qquad \text{by (123)}\\
&\geq \prod_{k=1}^{\infty}(1-b^{2k})\left(1-|\xi+\xi^{-1}|b^{2k-1}+b^{2(2k-1)}\right) \qquad \text{by (124)}\\
&= \prod_{k=1}^{\infty}(1-b^{2k})\left(1+((-x)+(-x)^{-1})b^{2k-1}+b^{2(2k-1)}\right) \qquad \text{by (128)}\\
&= \prod_{k=1}^{\infty}(1-b^{2k})\left(1+(-x)b^{2k-1}\right)\left(1+(-x)^{-1}b^{2k-1}\right)\\
&= \sum_{k\in\mathbb{Z}}(-x)^k b^{k^2} \qquad \text{by Jacobi's triple product identity (123)}\\
&= \sum_{k\in\mathbb{Z}} x^{2k}b^{(2k)^2} - \sum_{k\in\mathbb{Z}} x^{2k+1}b^{(2k+1)^2}\,.
\end{aligned} \tag{129}$$

We have (using that $x \geq 1$ by (127) and thus $x^{-2k} \geq 0$) that

$$\begin{aligned}
\sum_{k\in\mathbb{Z}} x^{2k}b^{(2k)^2} &= 1 + \sum_{k=1}^{\infty}(x^{2k}+x^{-2k})b^{(2k)^2}\\
&\geq 1 + \sum_{k=1}^{\infty} x^{2k}b^{(2k)^2}\\
&= \sum_{k=0}^{\infty} x^{2k}b^{(2k)^2}\,,
\end{aligned} \tag{130}$$

and

$$\begin{aligned}
\sum_{k\in\mathbb{Z}} x^{2k+1}b^{(2k+1)^2} &= \sum_{k=0}^{\infty}\left(x^{2k+1}+x^{-(2k+1)}\right)b^{(2k+1)^2}\\
&\leq 2\sum_{k=0}^{\infty} x^{2k+1}b^{(2k+1)^2}\\
&\leq 2\sum_{k=0}^{\infty}(b^{4k+1}x)x^{2k}b^{(2k)^2}\\
&\leq 2bx\sum_{k=0}^{\infty} x^{2k}b^{(2k)^2}\,.
\end{aligned} \tag{131}$$

By combining (129) with (130) and (131), we obtain

$$\begin{aligned}\left|\sum_{k\in\mathbb{Z}} \xi^k b^{k^2}\right| &\geq (1-2bx)\sum_{k=0}^{\infty} x^{2k} b^{(2k)^2}\\ &\geq (1-2bx)\sum_{k=0}^{\infty} (b^4)^{k^2}\end{aligned}$$

where we used that $x \geq 1$, see (127). Since we also have (also by (127)) that $x \leq \max\{|\xi|, |\xi|^{-1}\}$, the claim follows. $\square$

Supplementary References

[1] Ronald N. Bracewell. *The Fourier Transform and Its Applications.* Circuits Syst. McGraw-Hill, 2000.

[2] Lukas Brenner, Libor Caha, Xavier Coiteux-Roy, and Robert Koenig. Complexity of gottesman-kitaev-preskill states. *Phys. Rev. X*, 15:031073, Sep 2025.

[3] Daniel Gottesman, Alexei Kitaev, and John Preskill. Encoding a qubit in an oscillator. *Phys. Rev. A*, 64:012310, Jun 2001.

[4] Barbara M. Terhal and Daniel J. Weigand. Encoding a qubit into a cavity mode in circuit QED using phase estimation. *Phys. Rev. A*, 93(1), Jan 2016.

[5] Daniel J. Weigand and Barbara M. Terhal. Generating grid states from Schrödinger-cat states without postselection. *Phys. Rev. A*, 97(2), Feb 2018.

[6] Yunong Shi, Christopher Chamberland, and Andrew Cross. Fault-tolerant preparation of approximate GKP states. *New J. of Phys.*, 21(9):093007, Sep 2019.

[7] Jacob Hastrup, Kimin Park, Jonatan Bohr Brask, Radim Filip, and Ulrik Lund Andersen. Measurement-free preparation of grid states. *npj Quantum Inf.*, 7(1), Jan 2021.

[8] Peter W. Shor. Polynomial-time algorithms for prime factorization and discrete logarithms on a quantum computer. *SIAM J. Comput.*, 26(5):1484–1509, 1997.

[9] Antoni Zygmund. *Trigonometric Series.* Cambridge Math. Lib. Cambridge Univ. Press, 3rd edition, 2003.

[10] Daniele Micciancio and Oded Regev. Worst-case to average-case reductions based on Gaussian measures. *SIAM J. Comput.*, 37(1):267–302, 2007.

[11] Carl G.J. Jacobi. *Fundamenta nova theoriae functionum ellipticarum.* Sumtibus fratrum, 1829.

[12] Wojciech Banaszczyk. New bounds in some transference theorems in the geometry of numbers. *Math. Ann.*, 296(4):625–636, Dec 1993.

[13] Wojciech Banaszczyk. Inequalities for convex bodies and polar reciprocal lattices in Rn. *Discrete Comput. Geom.*, 13(2):217–231, March 1995.

[14] Nicholas Genise, Daniele Micciancio, Chris Peikert, and Michael Walter. Improved discrete gaussian and subgaussian analysis for lattice cryptography. In *Public-Key Cryptography – PKC 2020*, volume 12111 of *Lect. Notes Comput. Sci.*, pages 623–651, Springer, 2020.

[15] Chris Peikert. A decade of lattice cryptography. *Found. Trends Theor. Comput. Sci.*, 10(4):283–424, 2016.
